# Mutational signatures are jointly shaped by DNA damage and repair

**DOI:** 10.1038/s41467-020-15912-7

**Published:** 2020-05-01

**Authors:** Nadezda V. Volkova, Bettina Meier, Víctor González-Huici, Simone Bertolini, Santiago Gonzalez, Harald Vöhringer, Federico Abascal, Iñigo Martincorena, Peter J. Campbell, Anton Gartner, Moritz Gerstung

**Affiliations:** 1 European Molecular Biology Laboratory, European Bioinformatics Institute (EMBL-EBI), Hinxton, CB10 1SD UK; 2 Centre for Gene Regulation and Expression, University of Dundee, Dundee, DD1 5EH Scotland; 3 Cancer, Ageing and Somatic Mutation, Wellcome Sanger Institute, Hinxton, CB10 1SA UK; 4 Department of Haematology, University of Cambridge, Cambridge, CB2 0XY UK; 5 Department of Haematology, Addenbrooke’s Hospital, Cambridge, CB2 0QQ UK; 6 Center for Genomic Integrity, Institute for Basic Science, Ulsan, 689-798 Republic of Korea; 7 Department of Biological Sciences, School of Life Sciences, Ulsan National Institute of Science and Technology, Ulsan, 689-798 Republic of Korea; 8 European Molecular Biology Laboratory, Genome Biology Unit, 69177 Heidelberg, Germany; 9 Present Address: Institute for Research in Biomedicine (IRB Barcelona), Parc Científic de Barcelona, 08028 Barcelona, Spain

**Keywords:** Cancer genomics, Computational models, DNA damage and repair, Nucleotide excision repair, Translesion synthesis

## Abstract

Cells possess an armamentarium of DNA repair pathways to counter DNA damage and prevent mutation. Here we use *C. elegans* whole genome sequencing to systematically quantify the contributions of these factors to mutational signatures. We analyse 2,717 genomes from wild-type and 53 DNA repair defective backgrounds, exposed to 11 genotoxins, including UV-B and ionizing radiation, alkylating compounds, aristolochic acid, aflatoxin B1, and cisplatin. Combined genotoxic exposure and DNA repair deficiency alters mutation rates or signatures in 41% of experiments, revealing how different DNA alterations induced by the same genotoxin are mended by separate repair pathways. Error-prone translesion synthesis causes the majority of genotoxin-induced base substitutions, but averts larger deletions. Nucleotide excision repair prevents up to 99% of point mutations, almost uniformly across the mutation spectrum. Our data show that mutational signatures are joint products of DNA damage and repair and suggest that multiple factors underlie signatures observed in cancer genomes.

## Abstract (web summary)

Recent research has shown that mutational signatures reflective of the history of a cancer can be detected in a cancer genome. Here, using whole genome sequencing of DNA repair deficient and proficient nematodes exposed to genotoxins, the authors show that these mutational signatures reflect both the initial DNA damage that was inflicted and the repair processes that ensue.

## Introduction

A cell’s DNA is constantly altered by a multitude of genotoxic stresses including environmental toxins and radiation, DNA replication errors and endogenous metabolites, all rendering the maintenance of the genome a titanic challenge. Organisms thus evolved diverse DNA repair mechanisms to detect and mend DNA damage, and to eliminate or permanently halt the progression of genetically compromised cells. Nevertheless, some DNA lesions escape detection and repair or are processed by error-prone pathways, leading to mutagenesis—the process that drives evolution but also inheritable disease, ageing and cancer.

The multifaceted nature of mutagenesis results in distinct mutational spectra, characterised by the specific distribution of single- and multi-nucleotide variants (SNVs and MNVs), small insertions and deletions (indels), large structural variants (SVs), and copy number alterations. Studying mutational patterns yields insights into the nature of DNA damage and DNA repair processes. Indeed, the analysis of thousands of cancer genomes and exomes led to the discovery of more than 50 mutational signatures of base substitutions^1,2^. Some of these signatures, deduced by computational pattern recognition, have evident associations with exposure to known mutagens such as UV light, tobacco smoke, the food contaminants aristolochic acid (AA) and aflatoxins^3–5^, or with DNA repair deficiency syndromes and compromised DNA replication^1,6,7^. However, the aetiology of many computationally extracted cancer signatures still has to be established.

The association between mutational spectra and their underlying mutagenic processes is complicated as mutations arise from various DNA lesions, which are mended by numerous and partially redundant DNA repair pathways. Hence, there are at least two unknowns that contribute to a mutational spectrum: DNA damage and DNA repair. The fact that these counteracting processes are jointly shaping mutagenesis is perhaps best exemplified at the interplay of nucleotide misincorporation by replicative DNA polymerases and mismatch repair (MMR). MMR operates downstream of the replication fork and mends many mismatches caused by misincorporated nucleotides, often in a base-specific way. Thus, when polymerase fidelity is compromised, MMR provides a backstop and observed mutations stem from the subset of lesions that escaped MMR or were incorrectly repaired; the full spectrum of replication errors only becomes visible under MMR deficiency. Indeed, distinct mutational signatures have been observed in human cancers with concomitant POLE exonuclease mutations and MMR deficiency^8^ and also in *Caenorhabditis elegans* lines deficient for both the MMR factor *pms-2* and the polymerase epsilon subunit *pole-4*^9^.

Here, we experimentally investigate the counteracting roles of genotoxic processes and DNA repair. Using *C. elegans* whole-genome sequencing, we determined mutational spectra resulting from the exposure of wild-type and 53 DNA repair defective lines, encompassing most conserved DNA repair and DNA damage response pathways, to 11 genotoxic agents causing a diverse set of DNA alterations. Combining genotoxin exposure and DNA repair deficiency in many cases led to altered mutagenesis, signified by either higher or lower mutation rates or altered mutation spectra compared to DNA repair deficiency or wild-type genotoxin exposure alone. These interactions highlight how different DNA lesions induced by the same genotoxin are processed by a number of DNA repair pathways, often specific for a particular type of DNA damage, therefore changing mutation spectra usually in subtle but sometimes also dramatic ways. We confirm that similar interactions are also imprinted in cancer genomes and provide an explanation why such signatures are difficult to detect. Our data imply that mutational signatures derived from cancer genomes can be variable, and may not have a one-to-one relationship to distinct mutagenic processes.

## Results

### Experimental mutagenesis in *C. elegans*

**Fig. 1:**
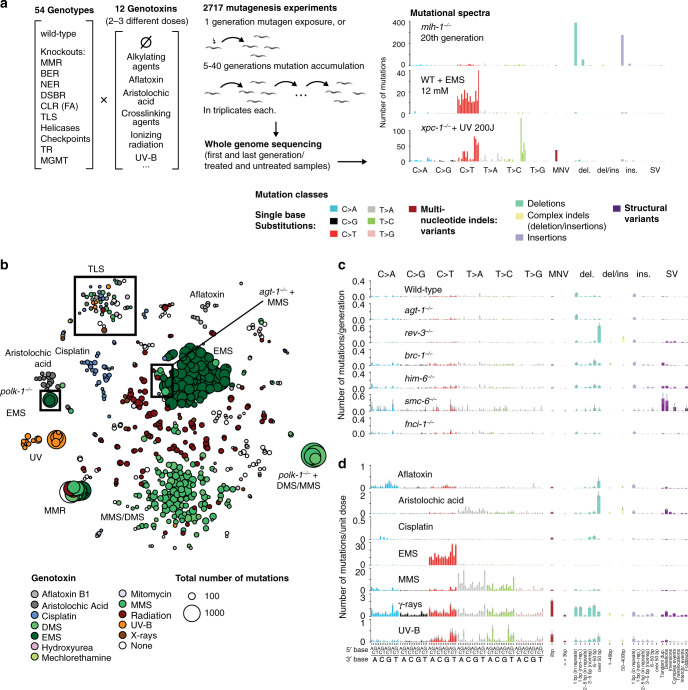
Experimental study design and data overview. **a** Left: *C. elegans* wild-type (WT) and mutants of selected genes from the indicated DNA repair pathways were propagated over several generations or exposed to increasing doses of 12 different genotoxins. Genomic DNA from samples before and after propagation or without and with treatment was sequenced to determine mutational spectra (Methods). Right: representative experimental mutational spectra across 119 mutation classes: 96 single base substitutions classified by the 6 possible base changes in their ‘5 and 3’ base context, di- and multi-nucleotide variants (MNV), 6 classes of deletions of different length and context, 2 classes of complex indels, 6 classes of insertions and 7 classes of structural variants (see panel **d** for the list of mutation classes). Individual bars represent the average number of mutations observed per mutation class. **b** 2D t-SNE representation of all *C. elegans* samples based on the cosine similarity between mutational profiles. Dots represent individual sequenced samples, with dot sizes proportional to the number of observed mutations. Colours depict genotoxin exposures, mutation accumulation samples are not couloured. Black boxes highlight selected genotoxins and DNA repair deficiencies. **c** Experimental mutational signatures of selected DNA repair deficiencies. Each bar corresponds to the estimated mean number of mutations of a particular mutation class per propagated generation. Error bars denote 95% credible intervals. See panel **d** for the list of mutation classes. **d** Experimental mutational signatures of selected genotoxin exposures in wild-type *C. elegans*. Each bar corresponds to the estimated mean number of mutations of a particular mutation class per generation per dose. Error bars denote 95% credible intervals.

To determine how the interplay between mutagenic processes and DNA repair status impacts mutational spectra, we used 54 *C. elegans* strains, including wild-type and 53 DNA repair and DNA damage sensing mutants (Fig. 1a). These cover all major DNA repair pathways including direct damage reversal (DR), base excision repair (BER), nucleotide excision repair (NER), MMR, DNA double-strand break repair (DSBR), translesion synthesis (TLS), DNA crosslink repair (CLR), and DNA damage sensing checkpoints (DS)^10–14^ (see Supplementary Table 1 for a full list). To inflict different types of DNA damage we used 12 genotoxic agents encompassing UV-B, X- and γ-radiation; the alkylating agents ethyl methanesulfonate (EMS), dimethyl sulfate (DMS) and methyl methanesulfonate (MMS); AA and aflatoxin B1, which form bulky DNA adducts; hydroxyurea as a replication fork stalling agent; and cisplatin, mechlorethamine (nitrogen mustard) and mitomycin C (which was only used in the wild-type) known to form DNA intra- and inter-strand crosslinks.

To investigate the level of mutagenesis for each genotype in the absence of exogenous mutagens, we clonally propagated wild-type and DNA repair defective *C. elegans* lines over 5–40 generations in mutation accumulation experiments (Methods, Fig. 1a)^9,15^. In addition, to measure the effects of genotoxic agents, nematodes were exposed to mutagenize germ cells and mutational spectra were analysed by sequencing clonally derived progeny^15^. Self-fertilising propagation through single zygotes provides clonally amplified DNA for subsequent whole-genome sequencing (Fig. 1a). Experiments were typically performed in triplicates, with dose escalation for genotoxin treatments.

Overall, we analysed a total of 2717 *C. elegans* genomes comprising 477 samples from mutation accumulation experiments, 234 samples from genotoxin-treated DNA repair proficient wild-type lines and 2006 samples from interaction experiments combining genotoxin treatment with DNA repair deficiency. The sample set harboured a total of 162,820 acquired mutations (average of ~60 mutations per sample) comprising 135,348 SNVs, 937 MNVs, 24,308 indels and 2,227 SVs. Attribution of observed mutations to each genotoxin, to distinct DNA repair deficiencies, and to their combined action was done using a negative binomial regression analysis leveraging the explicit knowledge of genotoxin dose, genotype, and the number of generations across all samples to obtain absolute mutation rates and signatures, measured in units of mutations/generation/dose (Methods).

### Mutation accumulation under DNA repair deficiencies

Comparing mutations in genomes of the first and final generations for the 477 mutation accumulation samples, we observed a generally low rate of mutagenesis in the range of 0.8–2 mutations per generation similar to previous reports on 17 DNA repair deficient *C. elegans* strains^15,16^ (Supplementary Note 1). Notable exceptions were lines carrying knockouts of genes affecting MMR, DSBR and TLS (Fig. 1c, Supplementary Note 1). MMR deficient *mlh-1* knockouts manifested in high base substitution rates combined with a pattern of 1 bp insertions and deletions at homopolymer repeats, similar to reports in human cancers (cosine similarity *c* = 0.85)^9,17^. DSBR deficient strains carrying a deletion in *brc-1*, the *C. elegans* ortholog of the *BRCA1* tumour suppressor, exhibited a uniform base substitution spectrum and increased rate of small deletions and tandem duplications (Fig. 1c), features also observed in *BRCA1* defective cancer genomes (*c* = 0.69 relative to SBS3)^18^. TLS polymerase knockouts of *polh-1* and *rev-3* yielded a mutational spectrum dominated by 50–400 bp deletions. We postulate that these deletions arise when replication over damaged bases is stalled or fails^19–21^ (Fig. 1c, Supplementary Note 1). Furthermore, *him-6* mutants, defective for the *C. elegans* orthologue of the BLM (Bloom syndrome) helicase, and *smc-6* mutants, defective for the Smc5/6 cohesin-like complex, showed an increased incidence of SVs (Fig. 1c). Experimentally derived mutational signatures for all 54 genetic backgrounds are summarised in Supplementary Note 1 and Supplementary Data 2. These findings are broadly consistent with data from human cancers, which exhibit hypermutation for deficiency of MMR, POLE exonuclease domain, and DSBR^1^, but barely for extremely common monoallelic or rarely occurring biallelic mutations in other DNA repair genes (Supplementary Fig. 1, Supplementary Note 3, Supplementary Data 3).

### Mutagenesis under genotoxic exposures

Genotoxins tended to have a stronger influence on the mutational spectrum compared to DNA repair deficient backgrounds (Fig. 1b; see Supplementary Fig. 2 and Supplementary Data 2 for a full list of spectra). The methylating agents MMS and DMS, produced very similar mutation spectra with high numbers of T > A and T > C substitutions (Fig. 1b, d).

Samples treated with the ethylating agent EMS were characterised by C > T mutations, somewhat similar to the mutational signature SBS11 observed in cancer genomes, which has been attributed to exposure to the alkylating agent temozolomide (cosine similarity *c* = 0.91)^2^. However, both signatures differ from the EMS spectrum reported in human iPS cells^22^ (Supplementary Fig. 2b), likely due to differential repair capacity.

Bulky adducts created by AA and aflatoxin B1 exposure resulted in mutational spectra with typical T > A and C > A mutations, respectively, similar to those observed in exposed human cancers and cell lines (*c* = 0.95 and *c* = 0.92, respectively)^4,23^. UV-B radiation induced characteristic C > T transitions in a YpCpH context (Y = C/T; H = A/C/T), similar to SBS7a/b (*c* = 0.95 for C > T alone), but with an unexpected rate of T > A transversions in a WpTpA context (W = A/T; *c* = 0.69 for all variants), which might be caused by the higher proportion of 6-4 photoproducts induced by UV-B compared to sunlight and possibly lower repair efficiency of these lesions in *C. elegans*^24^. Ionizing radiation (X- and ɣ-rays) caused single and multi-nucleotide substitutions, but also deletions and SVs, in agreement with the spectra of radiation-induced secondary malignancies^25^ (*c* = 0.89 for substitution spectra). Lastly, cisplatin exposure induced C > A transversions in a CpCpC and CpCpG context, deletions, and SVs in agreement with previous studies (Fig. 1b, d)^15,22,26,27^. Interestingly, the overall cisplatin-induced mutation spectrum seems strongly organism-specific (Supplementary Methods).

The general resemblance of genotoxin-derived mutational signatures across organisms (Supplementary Fig. 2, Supplementary Methods) reflects the high level of conservation of DNA repair pathways among eukaryotes. Interestingly, discrepancies observed between species or different cell lines derived from the same species may provide insights into differences in genotoxin metabolism or DNA repair capacity.

### Signatures of concomitant DNA damage and repair deficiency

The experimentally controlled exposure time and doses enabled us to quantify how strongly a DNA repair deficiency altered mutational effects of a genotoxin on a genome-wide scale. We considered a DNA repair deficiency and a genotoxin as interacting if the mutation rate of all or a type of mutations (e.g. substitutions, indels or SVs) changed relative to the expectation that the observed mutations can be expressed as the sum of the genotoxin spectrum in wild-type and the DNA repair deficiency spectrum without genotoxin exposure (Methods).

**Fig. 2:**
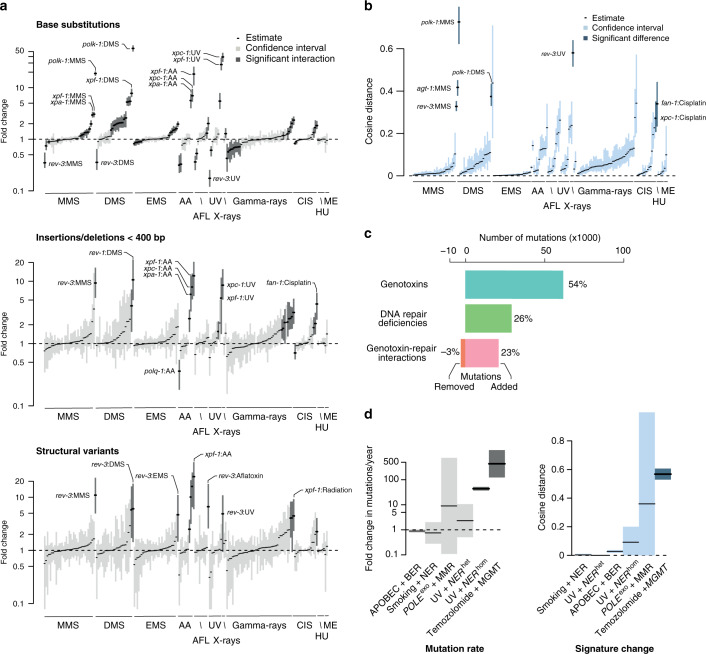
Widespread and diverse genotoxin-repair interactions in *C. elegans* and cancer. **a** Estimated fold changes between observed numbers relative to expected numbers of mutations based on additive genotoxin and DNA repair deficiency effects shown for base substitutions (upper panel), insertions/deletions (middle panel), and structural variants (lower panel) for all genotoxins. AFL - aflatoxin B1, CIS - cisplatin, HU - hydroxyurea, ME - mechlorethamine. A value of 1 indicates no change. Black lines denote point estimates. Interactions with fold changes significantly above or below 1 (as per testing whether the squared log ratio between mutation numbers induced by a genotoxin in mutants versus wild-type follows the $\chi$^2^ distribution at 5% FDR) are shown as dark grey, the rest as light grey bars with their width indicating confidence interval. **b** Changes in spectra of genotoxin-induced mutations. Black lines denote point estimates. Interactions with cosine distances larger than 0.2 are shown with dark blue confidence intervals, others are shown in light blue. **c** Numbers of mutations attributed to genotoxins (blue) and DNA repair deficiencies (green), as well as positive interactions (pink) and negative interactions (orange) between genotoxins and DNA repair deficiencies. **d** Summary of interaction effects between selected DNA repair pathway deficiencies and DNA damage-associated mutational signatures in human cancers. Left: Changes in age-adjusted mutation burden. Right: Change in mutational signatures. Black lines denote the average values and bars correspond to 95% confidence intervals. Significant interactions are shown in darker colour (as per testing whether the squared log ratio between mutation numbers expected in deficient versus proficient samples follows the $\chi$^2^ distribution at 5% FDR, or whether the cosine similarity between the spectra is lower than 0.8).

Genotoxin-repair deficiency interactions were very common: In total, 88/196 (41%, at false-discovery rate (FDR) < 5%) of the combinations we tested displayed an interaction between DNA repair status and genotoxin treatment involving 9/11 genotoxins and 32/53 genotypes (Fig. 2, for a comprehensive list of effects see Supplementary Note 2 and Supplementary Data 4). Most frequently interactions increased the numbers of mutations obtained for a given dose of mutagen, with the strongest increase of 50x observed in *polk-1* mutants under exposure to alkylating agents (Fig. 2a). Conversely, knockouts of TLS polymerases REV-3 and to a lower extent POLH-1 reduced the rate of point mutations for a range of genotoxins while showing increased numbers of indels and/or SVs (Fig. 2a). While some interactions left the mutational spectrum largely unchanged, such as MMS-exposure of *xpc-1* mutants, others, such as MMS- or DMS-exposure of *polk-1* mutants, had a profound impact on mutational spectra, indicating that this specific DNA repair pathway only mends a subset of lesions introduced by the genotoxin (Fig. 2b).

Overall, we estimate that of the 141,004 mutations observed in genotoxin exposure experiments in DNA repair deficient strains, 26% were attributed to the endogenous mutagenicity of DNA repair deficiency independent of genotoxic exposure and 54% were attributed to genotoxic exposures independent of the genetic background. In addition, 23% of mutations arose due to positive interactions between mutagen exposure and DNA repair deficiency leading to increased mutagenicity (Fig. 2c). Finally, we found that 3% of mutations were prevented by negative interactions between genotoxins and DNA repair deficiency leading to reduced mutagenesis.

Applying a similar approach to a range of DNA repair deficient and proficient cancers with suspected genotoxic exposures, we were able to characterise several cases of DNA damage-repair interactions leading to increased mutagenesis and/or altered signatures, such as POLE^EXO^ and MMR, tissue-specific changes of the MMR deficiency signature, UV damage and NER, or APOBEC-induced mutagenesis and TLS (Fig. 2d, Supplementary Fig. 3, Supplementary Note 4). However, we found that interactions were hard to detect due to the unknown history of exposure and DNA repair defects, and detectable effects were typically only moderate.

### Lesion-specific repair and mutagenicity of DNA alkylation

**Fig. 3:**
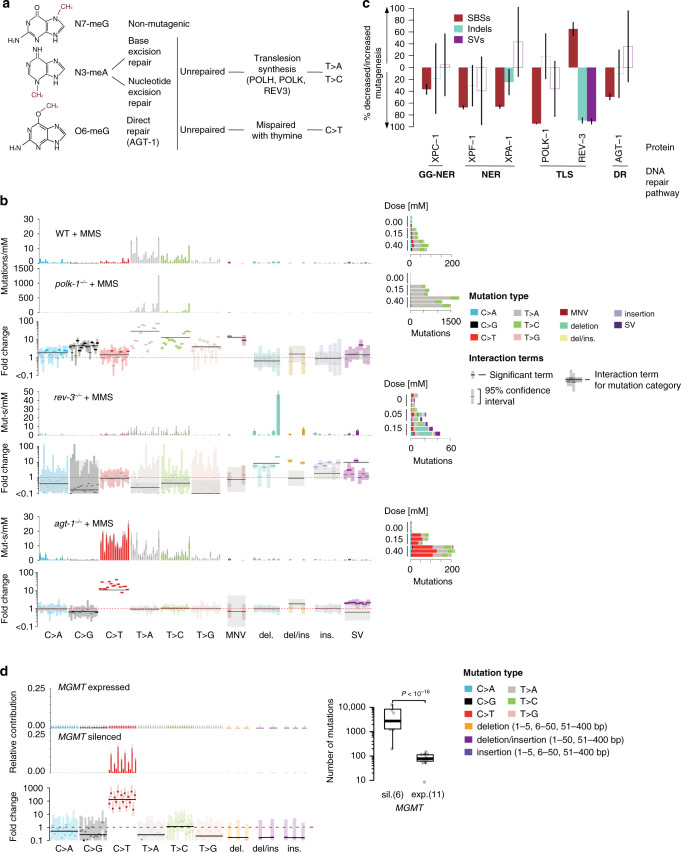
DNA repair mutant specific MMS-induced mutational signatures. **a** DNA repair and mutagenicity of different DNA lesions induced by MMS: chemical structures of non-mutagenic N7-methylguanine and mutagenic N3-methyladenine and O6-methylguanine are depicted. Methyl groups are indicated in red. **b** MMS-induced signatures in wild-type, *polk-1*, *rev-3* and *agt-1* deficient *C. elegans*. Top: Mean estimates of MMS-induced mutation spectra in wild-type and *polk-1*^−/−^. Error bars denote 95% credible intervals (CIs). Below: mutation rate fold changes per mutation class between the wild-type and interaction spectra with 95% CIs. Point estimates for significant fold changes are shown in darker colour (credible intervals do not cross 1, red dotted line). Centre: MMS-induced signature in *rev-3*^−/−^ shown with respective fold changes. Bottom: MMS-induced signature in *agt-1*^−/−^ shown with the respective fold changes. Right: Number of mutations per replicate shown across MMS doses in wild-type (upper part), *polk-1* mutants (centre part), and *agt-1* mutants (lower part). **c** Estimated mean fractions of mutations contributed or prevented by different DNA repair components compared to the mutations observed upon MMS exposure in wild-type. Bars with no fill colour reflect non-significant contributions (chi-squared test FDR 5% in each mutation category; SBSs - single base substitutions). Error bars (black) reflect 95% confidence intervals. **d** Mutational signatures of temozolomide in glioblastomas with expressed (top) and epigenetically silenced *MGMT* (centre) displayed as probability distributions across 104 mutation classes, coloured bars reflect the mean estimate of the probability of mutation for each mutation class, error bars represent 95% credible intervals. Bottom: mean mutation rate fold changes per mutation class with 95% confidence intervals, with significant fold changes marked by darker colour (if credible intervals do not cross 1, red dotted line). Right: Total mutation burden in temozolomide-treated samples with silenced (sil., *n* = 6) and expressed (exp., *n* = 11) *MGMT*. Boxes denote the interquartile range (IQR, 25–75% percentile), thick lines the median, whiskers - 1.5× the IQR below the first quartile and above the third quartile. Dots represent mutations in individual samples. MGMT silencing was significant as predictor of burden in a Poisson GLM (burden ~MGMT status, *p* value < 10^−16^).

Many genotoxins, such as the alkylating agent MMS, inflict DNA damage on different nucleotides and residues thereof (Fig. 3a). MMS induces several different base methylations, including non-mutagenic N7-meG, as well as mutagenic N3-meA and O6-meG^28^, which, taken together, produced a mutation rate of approximately 230 mutations/mM/generation in *C. elegans* wild-type (Fig. 3a). However, the underlying mutagenic and repair mechanisms for these DNA adducts are very different: O6-meG is subject to direct reversal by alkyl-guanine DNA alkyltransferases and tends to pair with thymine^29^, whereas N3-meA can be mended by BER or NER^30,31^. If unrepaired, N3-meA requires TLS polymerases for lesion bypass during replication, which can be error-free or result in the misincorporation of A or C opposite N3-meA^32^.

Indeed, our analysis showed diverse changes in the MMS-induced signature across DNA repair deficient backgrounds. In the absence of NER, the mutation rate of adenine was elevated 1.5× in *xpc-1* mutants and 3× in *xpf-1* and *xpa-1* mutants without a change in the mutational signature (Fig. 2a, b, Supplementary Fig. 4a), indicating that an increased proportion of N3-meA underwent error-prone TLS. These fold changes (FCs) correspond to about 30% and 60% of mutations being prevented by error-free XPC-1 and XPF-1/XPA-1 repair activity, respectively (Fig. 3c). A similar trend of 1.5–3-fold increase in T > M mutations was observed for EMS exposure under NER deficiency (Supplementary Fig. 4b). In contrast to MMS, EMS mostly induces O6-etG lesions with a small amount of N3-etA^29^. Consistent with this, we estimate that NER prevents about 25–40% of mutations upon EMS exposure (Supplementary Fig. 4c).

Deficiency of polymerase κ increased the overall mutation rate upon MMS treatment 17× with approximately 3,800 mutations/mM/generation (Fig. 3b). In line with the role of TLS in bypassing N3-meA during replication, polymerase κ deficiency yielded an approximately 10–100× higher rate of T > M (M = A/C) mutations, especially in TpTpN and CpTpN contexts (Fig. 3b). This increase also coincided with a distinct change in the mutational spectrum marked by a highly increased proportion of T > A transversions in a TpTpT context (Fig. 3b). These figures indicate that Pol κ dependent TLS is error-free and prevents 90–99% of DNA adducts caused by MMS exposure from becoming mutagenic (Fig. 3c). A similar increase of T > M substitutions (at the rate of 10×) was observed in Pol κ deficient mutants upon treatment with EMS, corresponding to 50% of EMS-induced mutations being prevented by POLK-1 mediated error-free TLS (Supplementary Fig. 4b, c). We postulate that in the absence of Pol κ, the bypass of alkylated adenines has to be achieved by other, error-prone TLS polymerases, leading to increased T > M mutagenesis, particularly in a TpTpT context.

One of the candidates for this error-prone TLS is *rev-3*, which encodes the catalytic subunit of TLS Pol ζ. In contrast to *polk-1* TLS deficiency where the mutation rate was increased, the knockout of *rev-3/*Pol ζ partially suppressed MMS-induced base substitutions but increased the number of small deletions (Fig. 3b), indicating that Pol ζ is an essential component for the bypass of alkylated bases. We estimated that about 60% of MMS-induced mutations in wild-type result from error-prone repair synthesis conferred by Pol ζ (Fig. 3c).

Combining MMS exposure with alkyl-transferase *agt-1* deficiency also led to a striking change in the MMS signature, increasing the C > T mutation rate from about 15 mutations/mM in the wild-type to over 200 in the mutant, while leaving the rate of T > M mutations unchanged (Fig. 3b). This demonstrates that AGT-1 specifically reverses most O^6^-methylguanine adducts in an error free manner, thus acting as the functional *C. elegans* ortholog of the human O^6^-methylguanine DNA methyltransferase *MGMT*. Thus, the repair activity of AGT-1 prevents 50% of mutations which would otherwise be induced by MMS (Fig. 3c). A similar, but weaker effect of *agt-1* deficiency was observed upon exposure to the ethylating agent EMS, leading to a 1.5-fold increase in C > T transitions, indicating that AGT-1 is also involved in, but less efficient at, removing ethyl groups, the latter result being consistent with reports measuring single locus reversion rates in *E. coli*^33^ (Supplementary Fig. 4b, c).

An even stronger interaction, which has already been therapeutically exploited, occurs between the human *agt-1* ortholog *MGMT* and temozolomide, a DNA alkylating chemotherapeutic, in temozolomide-treated glioblastomas (Fig. 3d)^34^. This is in good agreement with our experimental findings that the nature of mutation spectra detected upon EMS, DMS or MMS alkylation depends on the status of *agt-1* (Fig. 2a).

### TLS causes the majority of substitutions

**Fig. 4:**
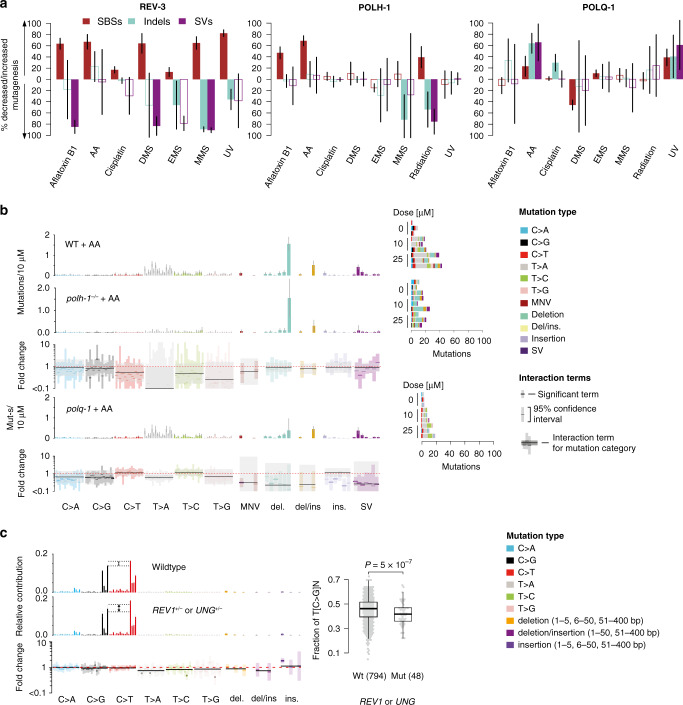
Error-prone TLS induces the majority of mutations observed upon genotoxin exposure. **a** Estimated mean fractions of mutations caused or prevented by three *C. elegans* TLS polymerases (REV-3, POLH-1 and POLQ-1) across genotoxin exposures along with 95% confidence intervals. Bars with no fill colour reflect non-significant contributions (chi-squared test FDR 5% in each mutation category). Layout as in Fig. 3c. **b** Aristolochic acid-induced signatures in wild-type and TLS polymerases η (*polh-1*) and θ (*polq-1*) deficient *C. elegans*. Layout as in Fig. 3b. **c** Interactions of APOBEC-induced DNA damage with deficiency in human REV1- or UNG-dependent BER. APOBEC mutational signatures of base substitutions in *REV1* and *UNG* wild-type (top) and deficient (centre) samples and signature fold changes per mutation class (bottom). Layout as in Fig. 3d. Right: Observed difference between the fractions of C > G and C > T mutations in a TCN context in samples with (mut) and without (wt) REV1 or UNG defects with sample sizes indicated in parentheses. Boxes denote the interquartile range (IQR, 25–75% percentile), thick lines the median, whiskers 1.5× the IQR below the first quartile and above the third quartile. Grey dots represent data from individual samples. Difference in the fraction of C > T mutations was significant between classes as assessed by a two-sided binomial test (*p* value ~5 × 10^−7^).

The contribution of certain low-fidelity TLS polymerases to genotoxin-induced mutagenesis is a wide-spread phenomenon^35^. Error-prone TLS is a key mechanism used to tolerate and bypass several types of DNA damage^19,36^, such as UV-induced cyclobutane pyrimidine dimers which stall replicative polymerases. Removal of polymerase ζ was shown to decrease the total frequency of mutations in yeast, mouse and human cells^37–39^. In *C. elegans*, exposing wild-type to UV-B led to approximately 10 mutations per 100 J m^−2^, comprised mostly of C > T and T > C transitions (Supplementary Fig. 5a). Paradoxically, the overall UV-B mutation rate was reduced 1.5-fold in *rev-3/*pol ζ mutants, mostly through a reduction in base substitutions, which was in contrast to a 4-fold increase of deletions longer than 50 bp (Supplementary Fig. 5a). To protect the genome from such deletions, which are likely to be deleterious, it is believed that TLS bypasses UV lesions at the cost of a higher base substitution rate^40^. Quantifying the amount of mutations resulting from the activity of TLS polymerases REV-3/Pol ζ, POLH-1/Pol η and POLQ-1/Pol θ, we observed that 60–80% of single base substitutions induced not only by UV, but also by aflatoxin B1, AA, DMS and MMS could be assigned to the activity of these polymerases (Fig. 4a, b). In contrast, REV-3/Pol ζ and POLH-1/Pol η also prevented 40–80% of indels and SVs following alkylating agents, UV and IR exposure (Fig. 4a). In contrast to previous reports^36,41,42^, only very small genomic interaction effects were observed in TLS mutants and cisplatin exposure, which was comparably low in this screen due to high lethality.

An inverse phenomenon is observed upon treating *polq-1*/Pol θ deficient mutants with AA. Indels, in particular deletions between 50 and 400 bp in length, induced by AA in wild-type were nearly absent in *polq-1* deficient mutants (Fig. 4b). Hence, we estimate that POLQ-1/Pol θ causes more than half of AA-induced indels and SVs (Fig. 4a, b). Breakpoint analysis of AA-induced deletions derived from all POLQ-1/Pol θ proficient lines demonstrated an excess of single base matches, indicative of *C. elegans* POLQ-1/Pol θ mediated end-joining^19,43^ (Supplementary Fig. 5b, c, Supplementary Methods). We speculate that in the absence of POLQ-1/Pol θ, replication-associated DSBs generated by persistent aristolactam adducts are likely to be repaired by HR, a slower but less error-prone pathway^44^.

A noteworthy example illustrating the role of TLS polymerases in cancer is conferred by APOBEC (apolipoprotein B mRNA editing enzyme, catalytic polypeptide-like) and error-prone, TLS-driven BER. APOBEC deaminates single-stranded cytosine to uracil, which pairs with adenine during replication, leading to C > T mutations. Uracil is thought to be removed by Uracil DNA Glycosylase UNG; subsequent synthesis by error-prone TLS REV1 leads to C > G mutations^45,46^. Indeed, a lack of C > G mutations has been observed in a cancer cell line with *UNG* silencing^47^. We found a relatively weak contribution of *REV1* and *UNG* mutation or silencing to APOBEC mutagenesis in human cancers, which nevertheless confirms the expected trend: on average, samples defective for *REV1* or *UNG* display an 8% decrease in C > G mutations (Fig. 4c). However, *REV1* and *UNG* status at diagnosis only explains a small fraction of APOBEC mutations with low rates of C > G transversion, suggesting that other processes contribute to these variants. Alternatively, the underlying signature change may not be detectable due to REV1/UNG mutation occurring late in cancer development compared to APOBEC overactivation.

### NER mends most genotoxic lesions

**Fig. 5:**
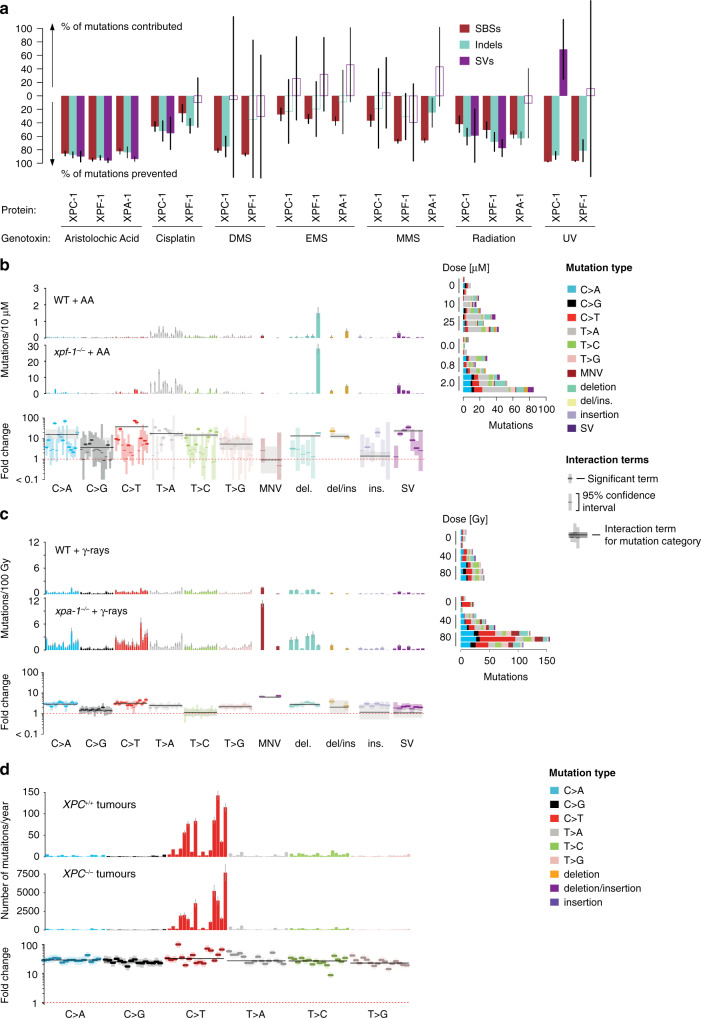
Nucleotide excision repair contributes to the repair of various genotoxin-induced damage. **a** Estimated mean fractions of mutations contributed and prevented by different NER components for various genotoxin treatments along with 95% confidence intervals. Bars with no fill colour reflect non-significant contributions (chi-squared test FDR 5% in each mutation category). Layout as described in Fig. 3c. **b** Signatures of aristolochic acid exposure in wild-type and *xpf-1* NER deficient *C. elegans*. Layout as described in Fig. 3b. *xpf-1* deficiency leads to a 10-fold increase in small deletions compared to the values expected without interaction. Note that scale bars are different between wild-type (WT) and *xpf-1*^−/−^ panels. c Signatures of γ-radiation in wild-type and *xpa-1* NER deficient *C. elegans*. Layout as described in Fig. 3b. *xpa-1* deficiency leads to a 2-fold overall increase in mutations, including a 5-fold increase in TCN > TTN changes and dinucleotide substitutions compared to the values expected without interaction. **d** Mutational signature of UV light in skin cutaneous squamous cell carcinoma (cSCC) samples from patients with functional (top panel) and mutated *XPC* (XP patients, central panel) expressed in mutations per year, and fold changes per mutation class (bottom panel). Note that scale bars are different between panels of XPC^+/+^ and XPC^−/−^ tumours with on average 30× more mutations in GG-NER deficient tumours.

NER acts by excising a large variety of damaged bases, preventing up to 90% of mutations induced by different genotoxins, particularly those which induce damage to adenine or thymine (Fig. 5a). After AA exposure, *C. elegans xpf-1* mutants showed a 5-fold increase in mutation rate with a 20-fold increase in the number of 50–400 bp deletions, confirming that NER is crucial for the repair of bulky DNA adducts (Fig. 5b). Aristolactam adducts occur on adenine^48^, and the failure to excise the modified base would lead to T > A changes or deletions. In contrast to previous reports on the lack of recognition of the aristolactam adducts by global genome NER (GG-NER)^49^, *xpc-1* mutants (which are defective for GG-NER) showed increased numbers of mutations upon AA exposure, especially deletions of 50–400 bps, similar to *xpf-1* mutants which are deficient in both GG-NER and transcription coupled NER (TC-NER) (Supplementary Fig. 6a). Interestingly, only *xpf-1* (which is defective for both TC-NER and GG-NER) but not *xpc-1* deficiency led to an increase in the number of C > A mutations, suggesting that TC-NER maybe be more crucial to the repair of dG aristolactam adducts, which tend to be mispaired with adenine during Pol η mediated TLS^50^.

In contrast to the previous examples in which DNA repair deficiencies changed the mutational spectra induced by mutagens, knockouts of the NER genes *xpf-1* and *xpc-1* did not alter mutation spectra but uniformly increased the rate of UV-B induced mutagenesis by a factor of 20 and 32, respectively (Fig. 2a, Supplementary Fig. 6b). This uniform increase indicates that NER is involved in repairing the majority of mutagenic UV-B DNA damage including both single- and multi-nucleotide lesions. Interestingly, *xpf-1* and *xpa-1* knockouts also uniformly increased the mutation burden resulting from EMS and MMS alkylation by a factor of 2, indicating that NER also contributes to error-free repair of alkylation damage (Supplementary Fig. 4). Further cases of genotoxin signatures being changed in NER mutants were observed following ionizing radiation and cisplatin exposure (Fig. 5c, Supplementary Fig. 6c). In IR treated samples, *xpa-1* deficiency led to a 10-fold increase in C > T changes as well as MNVs. Upon cisplatin exposure, NER defects increased the amount of C > A and T > A mutations, as well as dinucleotide substitutions likely introduced during the bypass of cisplatin-induced crosslinks^50,51^. In addition, in the *fan-1* mutant, defective for a conserved nuclease involved in ICL repair, cisplatin exposure led to a dramatic elevation of 50–400 base deletions without affecting base substitutions (Supplementary Fig. 6c).

In humans, xeroderma pigmentosum (XP), a hereditary syndrome characterised by an extreme sensitivity to UV exposure and high risk of skin and brain cancers during childhood, is often associated with biallelic inactivation of NER^52^. Investigating the changes in the UV signature between 8 adult skin tumours and 5 tumours from XPC defective XP patients^53^, we observed a relatively uniform 30-fold change in mutation rate per year in XP patients across base substitution types in line with findings in *C. elegans* (Fig. 5d). Moreover, there was a mild shift in certain base contexts, with nearly 3 times more mutations acquired in NpCpT, but also TpCpD context (D = A/G/T). XPC deficiency inactivates GG-NER, which is possibly compensated by transcription coupled NER^53^. It is thus possible that this shift in mutation spectrum reflects a sequence specificity of GG-NER repair efficiency.

Notably no effects were observed for NER variants in sporadic lung and skin cancers, although one might expect NER involvement in repairing bulky DNA adducts generated from tobacco smoke and UV light (Supplementary Fig. 3e–g). Of note *ERCC2/XPD* NER mutations are also relevant in urothelial cancers, where they have been reported to produce a mild increase in the number of mutations attributed to COSMIC signature 5^54^.

## Discussion

Taken together, our experimental screen and data analysis show that mutagenesis is fundamentally driven by the counteraction of DNA damage and repair. A consequence of this interplay is that the resulting mutation rates and signatures vary in a number of ways. The systematic nature of the screen with multiple known doses of different genotoxins applied across a broad range of genetic backgrounds enabled us to precisely characterise how mutation patterns of genotoxic treatments change under concomitant DNA repair deficiency.

Uniform mutation rate increases, without a change of mutational signature, suggest that a repair gene or pathway is involved in repairing all DNA lesions generated by the genotoxin. Examples of such interactions are NER deficiency combined with the exposure to UV-B damage, bulky DNA adducts, and alkylating agents. Conversely, mutational signatures change if divergent repair pathways are involved in repairing specific subsets of DNA lesions introduced by the same genotoxin. We illustrated this for alkylating agents, with the same adducts at different bases and residues being repaired by distinct pathways. In the case of DNA methylation, our data corroborate the notion that the mutagenicity of O6-methylguanine stems from mis-pairing with thymine, and is repaired by O6-alkylguanine DNA alkyltransferases, while N3-methyladenine stalls replication, which is resolved by TLS polymerases Pol κ and Rev-3/Pol ζ.

Our screen revealed that TLS plays an important and varied role in mutagenesis: perhaps counterintuitively, error-prone TLS by Pol η (POLH-1) and ζ (REV-3) was found to *cause* the majority of base substitutions resulting from bulky adducts, alkylated bases, UV-B-induced damage and to a small extent cisplatin. Thus, knockouts of *rev-3* and *polh-1* resulted in reduced base substitution rates, but increased rates of large deletions and SVs, presumably due to replication stalling and fork collapse in the absence of TLS. Conversely, polymerase κ (POLK-1) was found to prevent up to 99% of mutations induced by DNA alkylation, by performing largely error-free TLS across N3-meA and N3-etA. Lastly, Pol θ (POLQ-1) mediated deletions observed upon genotoxic exposures such as AA provide a repair mechanism (TMEJ) for replication-associated DSBs^19^. These findings imply that TLS—rather than the primary mutagenicity of genotoxic lesions—is an essential component of mutagenesis, and suggest a direction for further exploration in human tissue culture experiments.

With the exception of known hypermutator conditions, the association of mutations in DNA repair genes and mutational signatures in human cancer data proves challenging, due to the unknown dose of mutagene exposure and the unknown timing of both mutagen exposure and repair deficiency. The association is further complicated realising that many of the experimental genotoxin repair interactions described here had a moderate magnitude. Finally, while non-silent mutations in DNA repair genes are common, we found that bi-allelic inactivation of both copies generally required for loss of function is rare (Supplementary Note 3).

Yet to appreciate the implications of our experimental results for cancer development, it is worth reflecting on the fact that all human cells are subject to continuous, lifelong exposures to a variety of cell intrinsic and exogenous mutagenic processes^55^. Cancer is driven by this cumulative and lifelong action of mutational processes, which each incrementally add to cancer risk. Even more so, cancer transformation requires between 2 and 10 driver gene point mutations^56^, and therefore the relation between mutation rate and cancer development is not linear but polynomial, implying that even small changes in mutation rate exert large cancer-promoting effects^57^.

**Fig. 6:**
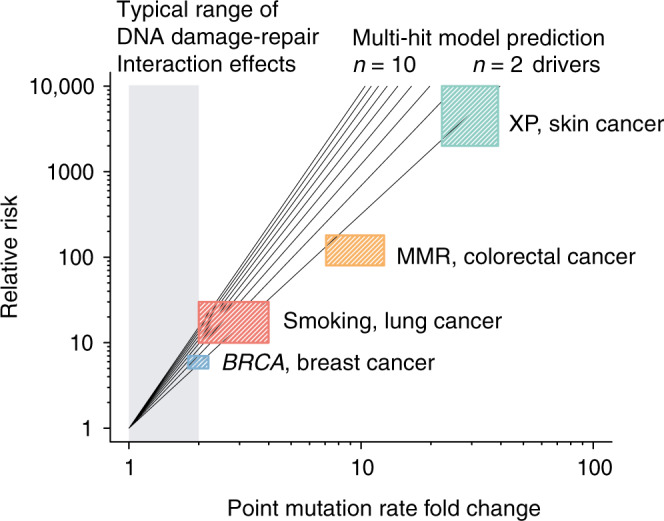
DNA repair deficiency drives cancer evolution. Relationship between relative cancer risk and point mutation rate change for different DNA repair pathway gene mutations. Black lines depict the expected power law depency in an evolutionary model with different numbers (from *n* = 2 to *n* = 10) of driver gene mutations^57^. Coloured boxes represent the ranges of incidence and mutation rate fold changes for indicated combination of cancer type and DNA repair deficiency or exposure. The grey shaded area denotes the range of less than 2-fold increase in mutation rate typically observed in our experimental screen, which may correspond to up to 10-fold increase in cancer incidence.

The fact that moderate changes in lifetime mutation burden cause large increases in cancer risk can be appreciated for a range of cancer predisposition syndromes, and smoking-related lung cancers (Fig. 6). While MMR-deficient colorectal cancers have an ~8–10-fold higher mutation burden than MMR-proficient carcinomas, inherited MMR deficiency increases colorectal cancer risk >115-fold^57^. Similarly, while HR-deficient breast cancers only display a ~3-fold higher mutation burden, this correlates with a 20–40-fold increased cancer risk for carriers of *BRCA1/2* mutations^58^. Even more so, XP patients display a 30-fold increased mutation rate^53^, but have an approximately 10,000-fold increased rate of skin cancers^52^. An implication of these data and models is that even fairly small changes in mutation rates have a large impact on cancer risk, and conversely noticeable risk factors may derive from rather moderate mutagenic effects. These findings offer an explanation why genes with no overt detectable mutator phenotype may be positively selected in cancers (Supplementary Fig. 3h, Supplementary Note 3). Furthermore, they underscore the need to accurately determine seemingly small and subtle mutagenic effects, which are challenging to detect in cancer genomes, but are often found in our experimental screen (Fig. 2a, b).

The analysis of mutational signatures derived from cancer genomes has gained much attention in recent years and dramatically deepened our understanding of the range and common types of mutation patterns observed in human cancer and normal tissues. However, our study suggests that not all genotoxins or DNA repair deficiencies yield unique mutational signatures due to mutual interactions, or simply manifest as an increased rate without a change in mutational signature as in the case of NER deficiency. It thus appears important to recognise the variable nature of mutagenesis due to the eternal struggle between DNA damage and repair. The high efficiency and redundancy of DNA repair processes removes a large fraction of genotoxic lesions and only damage left unrepaired results in mutations—either directly by mispairing or indirectly through error prone repair and tolerance mechanisms such as TLS. Mutation patterns observed in normal and cancer genomes represent the joint product of DNA damage, repair and tolerance.

## Methods

### Experimental design

*C. elegans* strains backcrossed 6× against the wild-type reference TG1813^15^ and mutagen doses are described in Supplementary Table 1.

### Mutation accumulation experiments

For mutation accumulation experiments 5–10 single L4 stage hermaphrodites (F1s) were randomly chosen from wild-type as well as each mutant (P0, 6× backcrossed against wild-type) and transferred to individual 1× NGM plates seeded with OP50 bacteria. Every 3–4 days, 1 single L4 hermaphrodite was randomly chosen among the progeny per plate and propagated further, a process repeated until the indicated generation (F5, F20 and F40). The final generation was then starved and used for DNA isolation and glycerol stocks. For each genotype, the P0 or F1 generation plate and at least three plates of the final generation lines were chunked to 9 cm 3× NGM plates and allowed to reach starvation. Worms were then washed off plates, washed 3× in M9 medium, pelleted and frozen in liquid nitrogen, before DNA extraction (below).

### Genotoxin treatment

Gravid adult worm populations were washed off 1× NGM plates with M9 medium, pelleted by centrifugation for 2 min at 400 × *g*, and washed with M9 to remove bacteria. Pelleted worms were then bleached and the released eggs resuspended and washed 3 times in M9 medium. Eggs were left in M9 for 24 h under gentle shaking to permit hatching and synchronisation at early L1 stage and were then transferred to OP50-seeded NGM plates. Once the population reached the young adult stage, 20 worms were picked for each genotype and genotoxin dose and incubated for 16 h at 20 °C in OP50-containing M9 medium with the desired concentration of EMS, DMS, MMS, aflatoxin B1 and AA. After the incubation, worms were centrifuged, transferred to OP50-seeded NGM plates and allowed to recover for 24 h. For cisplatin, mechlorethamine, and mitomycin C treatment 15–20 L4s were picked per genotype and genotoxin dose and were transferred as 24 h post L4 stage adults into OP50-containing M9 medium containing the indicated genotoxin concentrations. Genotoxins were diluted from freshly prepared stock solutions. Incubation was performed for 16 h at 20 °C under the hood in a shaking incubator. Worms were then transferred and left to recover for 24 h on OP50-seeded NGM plates. For X-ray exposure 15–20 L4 larvae were picked into M9 medium per genotype and dose. Irradiation was performed with the indicated radiation doses using a low energy photon radiosurgery system X-ray source. Worms were then transferred and allowed to recover for 24 h on OP50-seeded NGM plates. For γ-ray exposure 15–20 L4 larvae were picked onto OP50-containing NGM plates per genotype and dose. Worms were irradiated with the indicated radiation doses using a ^137^Cs source (2.14 Gy min^−1^, IBL 437C, CIS Bio International) and allowed to recover for 24 h. For UV treatment, early L1s, obtained after bleaching and synchronisation, were transferred to OP50-seeded NGM plates and irradiated at the indicated UV dose, in triplicate. Once grown to the young adult stage, 3 × 3 worms per genotype and dose were transferred to a fresh plate for progeny viability testing and propagation. For HU-treatments ~100 L1s were placed onto HU-containing OP50-seeded NGM plates and allowed to grow for 57 h. Worms were then transferred to new plates without HU and allowed to recover for 15 h.

### Progeny viability scoring and F1 isolation

Following genotoxin treatment and recovery 3× three worms/genotype and dose were transferred to new plates and allowed to lay eggs for 6 h. Adults were then removed. To determine progeny viability, the number of eggs laid as well as the number of unhatched eggs after 24 h was counted. The percentage of hatched eggs compared to the total number of eggs laid was calculated for each plate, and the mean and SD of each technical triplicate calculated. A minimum of three independent experiments (biological triplicate) was performed for each genotoxin. To obtain F1 lines for DNA sequencing, two F1 L4 larvae were picked from each of the three plates for each genotype and dose, transferred individually to a fresh plate and allowed to proliferate. These duplicates enhanced the probability to obtain one fertile line from each plate especially from animals treated with high genotoxin doses. From each duplicate, one line was selected for expansion (see MA experiment above), genomic DNA extraction and sequencing. The zygotes which lead to the F1 generation provide a single cell bottleneck where mutations of exposed male and female germ cells are fixed before being clonally amplified during *C. elegans* development and passed on to the next generation in a mendelian ratio.

### DNA extraction

Genomic DNA was isolated from frozen samples using ChargeSwitch^®^ gDNA Mini Tissue Kit (Invitrogen) following manufacturers instructions albeit with Proteinase K incubation O/N.

### Data acquisition

*C. elegans* genomic DNA was sequenced using Illumina HiSeq 2000 and X10 short read sequencing platforms at 100 bp paired-end reads with a mean coverage of 50×. Raw sequencing reads were aligned to the *C. elegans* reference genome (WBcel235/ce11 build) using BWA algorithm^59^. TCGA human cancer data was taken from NCI GDC (https://gdc.cancer.gov^60^), and respective studies^34,61–66^.

### Mutation calling, filtering, and classification

*C. elegans* aligned sequence reads were processed through the Sanger Cancer IT pipeline using CaVEMan for SNV calling^67^, Pindel for indel calling^68^, and DELLY^69^ for SV identification. Filtering of samples was performed using the same criteria as in ref. ^9^. Base substitutions were called using CAVEMAN^67^ with the following filtering criteria:

1. Coverage of the variant site in both the sample of interest (test sample) and reference (untreated F0 wild-type sample) did not exceed 150 reads or recede below 15 reads.
2. No reads reported the variant in the reference sample.
3. At least 20% of reads and at least 5 reads reporting the variants in test sample.
4. At least one read in the test sample reporting the variant in each read direction.
5. No indel was called at the same position (relevant for homopolymer junctions).

All variants were filtered against the normal panel of six untreated F1 wild-type samples to remove recurrent technical errors. Propagated samples (of generation > F1) of the same genotype from mutation accumulation experiments were not compared to each other to avoid overfiltering of related samples (e.g. when F40 lines were derived from the same F20 lines sequenced in the same study). Multiple substitutions which were found at adjacent sites in the same sample were classified as dinucleotide or MNVs, if their variant allele frequencies were similar (difference less than 5%). Overall, base substitutions were classified into 96 SNV classes (6 base substitution types per 16 trinucleotide contexts) and 2 MNV classes (dinucleotide variants and variants longer than 2 bp).

Small size insertions and deletions (1–400 bp) were called using PINDEL^68^ with the following filter criteria:

1. Coverage of the variant site in both the sample of interest and reference did not exceed 150 reads or recede below 10 reads.
2. No more than 1 read reporting the variant in the reference sample.
3. More than 20% of reads and at least 5 reads reporting the variants in test sample.
4. At least one read in test sample reporting the variant in each direction.
5. If the variant falls into a repetitive region, the region should not exceed 18 repeats.

All variants were filtered against the normal panel of six wild-type samples; samples of the same genotype and generation higher than one were not compared to each other to avoid overfiltering of related samples. Indels were classified into 14 classes based on indel type (deletions, insertions, and complex indels—named deletions-insertions (DI)), size (1, 2–5, 5–50 and 50–400 bp), and presence in repetitive or non-repetitive sequence contexts.

SVs were called using DELLY^69^ with the following filters:

1. Each variant was supported by more than ten high-quality reads in test sample and no reads were present in the reference sample.
2. Variant passed the default DELLY quality filter (paired-end support <5 for translocations or <3 for other SVs, or mapping quality <20).
3. Variants in telomeric regions were removed.
4. Duplicated SVs across unrelated samples were removed. Samples of the same genotype and generation higher than 1 were not compared to each other to avoid overfiltering of related samples.

The resulting sets of breakpoints for each sample were further classified in line with ref. ^70^ using clustering by proximity and simplified into a set of seven SVs: tandem duplications, deletions, inversions, intrachromosomal translocations, interchromosomal events, foldbacks (change of the orientation of sequence without a second breakpoint in close proximity) and complex variants (not falling in any of the previously described categories). Stand-alone deletions and tandem duplications were additionally tested for reliability by comparing the ratios of the coverages between the test and control samples outside the variant breakpoints and between them, to ensure that there is a drop in coverage for a deletion and an increase for a duplication.

Handling of variant calling results, filtering and classification were performed using R package VariantAnnotation-1.28.10^71^. Bam file statistics necessary to perform coverage comparison within and outside of deletions and tandem duplications was performed using samtools-0.1.18^72^. Mutation counts for all samples are listed in Supplementary Data 1. Data visualisation was performed using *t*-SNE^73^ based on the cosine distance of the 119-dimensional mutation spectra, averaged across replicates.

### Extraction of interaction effects from experimental data

For each sample *i* = 1, …, 2717 and mutation class *j* = 1, …, 119, we counted the respective number of mutations per sample *Y*_*i*_^*j*^. Using matrix notation, the counts **Y** $\in$ ${\Bbb N}_0^{2717 \times 119}$ (where ${\Bbb N}_0$ means the set of natural numbers and zero) were modelled by a negative binomial distribution

$${\mathbf{Y}}\sim {\mathrm{NB}}({\mathbf{\upmu}},\, {\varphi} = 100),$$

with scalar overdispersion parameter $\varphi$ = 100 selected based on the estimates of overdispersion in the dataset, and matrix-variate expectation ${\mathbf{\upmu }} \in {\Bbb R}_{ + 0}^{2717 \times 119}$, where ${\Bbb R}_{ + 0} = {\Bbb R}_{+} \cup \{ 0\}$.

The basic structure of the expectation is

1 $${\mathbf{\upmu }} = {\mathbf{\upmu }}_{\mathrm{G}}\left( {{\mathbf{g}} + {\mathbf{\upalpha }}_{\mathrm{I}}} \right) + {\mathbf{d}} {\cdot} {{\mathbf{\upmu}}_{\mathrm{M}}} \cdot {{\mathbf{\upbeta}}_{I}},$$

where **g** ∙ **μ**_G_ $\in {\Bbb R}_{ + 0}^{2717 \times 119}$ describes the expected mutations just due to the sample’s genotype in the absence of a genotoxin after **N** $\in {\Bbb Z}_0^{2717 \times 1}$ generations. The vector **g** $\in {\Bbb R}_{ + 0}^{2717 \times 1}$ denotes the generation number in every sample, ${\mathrm{g}}_{\mathrm{k}} = {\mathrm{g}}_{\mathrm{k}}\left( {{{N}}_{{k}}} \right) = \mathop {\sum}\nolimits_{i = 1}^{N_k} {2^{1 - i} + \frac{1}{4}\mathop {\sum}\nolimits_{i = 1}^{N_k - 1} {\mathop {\sum}\nolimits_{j = 1}^i {2^{1 - j}} } }$, *k* = 1, …, 2717, adjusted for the chances of fixation after *N*_*k*_ generations given the 25–50–25% probability of each mutation becoming homozygous, remaining heterozygous or being lost in each generation.

The matrix **d** ∙ **μ**_M_ $\in {\Bbb R}_{ + 0}^{2717 \times 119}$ denotes the expected mutation counts induced by mutagens at doses **d** $\in {\Bbb R}_{ + 0}^{2717 \times 1}$ in wild-type conditions. The terms **α**_I_ $\in {\Bbb R}_{ + 0}^{2717 \times 1}$ and **β**_I_ $\in {\Bbb R}_ + ^{2717 \times 119}$ model how these terms interact (more details follow below). Here, the dot product ‘∙’ is element-wise and we use the convention that a dimension of length 1 is extended by replication to match the length of the corresponding dimension of other factors in the product.

The expected number of mutations induced by genotypes, **μ**_G_, has the structure

2 $${\mathbf{\upmu }}_G = {\mathbf{G}}\, \times {\mathbf{S}}_{\mathrm{{G}}},$$

where **G** $\in {\Bbb Z}_2^{2717 \times 54}$ is the binary indicator matrix for denoting which one of the 54 genotypes a given sample has (*G*_*i*_^*j*^ = 1 if sample *i* has genotype *j*, otherwise *G*_*i*_^*j*^ = 0). The matrix

**S**_G_ $\in {\Bbb R}_ + ^{54 \times 119}$ denotes the mutation spectrum (i.e. signature) across the 119 mutation classes for each of the 54 genotypes in the absence of genotoxic exposures. To provide a more stable estimate for genotype signature, the prior distribution for **S**_G_ is derived from **S**_G_^*^ = **S**_G_ | **Y**_MA_, where **Y**_MA_ $\in {\Bbb Z}_0^{474 \times 119}$ is the matrix of counts coming from mutation accumulation samples only, and **S**_G_^*^ has a log-normal prior distribution **S**_G_^*^ ~logN(0, *σ*_G_^2^) iid with a scalar variance *σ*_G_^2^. Thus, we obtained priors for 51 genotypes. *agt-1*, *exo-1* and *rad-51* mutant strains did not have MA experiments, so they were assigned with **S**_N2_^*^, wild-type prior. The prior for **S**_G_ was defined as **S**_G_ ~ Г(**shape**_G_, **rate**_G_), where the matrix parameters **shape**_G_, **rate**_G_ were fitted to the posterior draws for **S**_G_^*^.

Similarly, the expected mutations contributed by mutagens at their unit doses, **μ**_M_, reads

3 $${\mathbf{\upmu }}_{\mathrm{M}} = {\mathbf{M}}\, \times {\mathbf{S}}_{\mathrm{M}},$$

with **M** $\in {\Bbb Z}_2^{2717 \times 12}$ being the indicator for the 12 mutagens (including Mitomycin C, which was eventually not used in the interaction experiments) and **S**_M_ $\in {\Bbb R}_ + ^{12 \times 119}$ being their signatures in wild-type, with per-column prior **S**_M_^(j)^ ~logN(0, **σ**_M*j*_^2^), *j* = 1, … 12, where the variances **σ**_M_^2^ = (σ_M1_^2^, …, σ_M12_^2^) have prior distributions σ_M*j*_^2^ ~ Г(1, 1) iid.

Thus, using the definitions (2) and (3), the matrix-variate expected number of mutations from (1) can be written as

4 $${\mathbf{\upmu }} = ({\mathbf{g}} + {\mathbf{\upalpha }}_{\mathrm{I}}) \cdot \left( {{\mathbf{G}} \, \times {\mathbf{S}}_{\mathrm{{G}}}} \right) + {\mathbf{d}} \cdot {\mathbf{\upbeta }}_I \cdot \left( {{\mathbf{M}}\, \times {\mathbf{S}}_{\mathrm{{M}}}} \right).$$

The vector-variate interaction term **α**_I_ measures how the mutations expected for genotypes G may uniformly increase under mutagen exposure and is taken to be linear with respect to the mutagen dose

5 $${\mathbf{\upalpha }}_{\mathrm{I}} = {\mathbf{d}} \cdot \left( {{\mathbf{I}}\, \times {\mathbf{b}}_{\mathrm{I}}} \right) + {\mathbf{\upvarepsilon }},$$

The symbol **I** $\in {\Bbb Z}_2^{2717 \times 196}$ is the binary indicator matrix for each one of the 196 interactions tested in the screen multiplied by the interaction rate **b**_I_ $\in {\Bbb R}_ + ^{196 \times 1}$, which measures how much the mutational signature of the genetic background increases upon genotoxic exposure with dose *d*. The interaction term is modelled to have a prior exponential distribution **b**_I_ ~ Exp(1) iid.

Lastly, ${\mathbf{\upvarepsilon }}$ is a random offset to allow for a possible divergence of the genotypes between experiments, adding ${\mathbf{\upvarepsilon }}$ ∙ ${\mathbf{\upmu }}$_G_ mutations with the same spectrum as in the absence of a mutagen in a dosage-independent fashion. The random offset is modelled as ${\mathbf{\upvarepsilon }}$ = (**J** × **a**_J_), where **J**$\,\, \in {\Bbb Z}_2^{2717 \times 208}$ is an extended binary indicator matrix for each one the 196 interactions and 12 wild-type exposure experiments, with value **a**_J_ $\in {\Bbb R}_{ + 0}^{2717 \times 1}$, **a**_J_ ~Exp(1) iid.

In addition to this scalar interaction, the wild-type spectrum of the mutagen **S**_M_ may change by the factors **β**_I_ $\in {\Bbb R}_ + ^{2717 \times 119}$ measuring the FC of each mutation class which is expressed as

6 $${\mathbf{\upbeta }}_{\mathrm{I}} = {\mathrm{exp}}({\mathbf{I}} \, \times {\mathbf{S}}_{\mathrm{I}}),$$

where **S**_I_ $\in {\Bbb R}^{196 \times 119}$ is the FC for each mutation class in a given interaction.

The prior distributions for each of the 119 subclasses of mutations in **S**_I_ are calculated in groups of 11 main mutation categories (6 types of SNVs, MNVs, 3 types of indels, SVs), since the numbers for individual mutation classes were sometimes very small. The prior was defined using **S**_I_ $\in {\Bbb R}^{196 \times 11}$ from an analogous model as described above, but applied only to observed mutation counts summed into 11 main mutation categories **Y** $\in {\Bbb N}_0^{2717 \times 11}$. We used a Laplace prior **S**^(j)^_I_ ~Laplace(0, *σ*^2^_Ij_), *j* = 1, …, 196, first to calculate **S**_I_|**Y**. The posterior expected value **S**_I_^*^ = E[**S**_I_|**Y**] was chosen as the prior expectation for the 119-dimensional mutation effects **S**_I_, **S**_I_^(j)^ ~ Laplace(**S**_I_^*^ × **C**, *σ*_Ij_^2^), *j* = 1, …, 196, where **C** is the matrix spreading mutation category value across the corresponding individual mutation classes, and the variances **σ**_I_^2^ = (*σ*_I1_^2^, …, *σ*_I196_^2^) and **σ**_I_^2^ = (*σ*_I1_^2^, …, *σ*_I196_^2^) were assumed to come from $\Gamma \left( {1,1} \right)$ iid.

Bayesian estimates of the parameters **S**_G_, **S**_M_, **S**_I_, **a**_J_, **b**_I_ and hyperparameters **σ**_I_^2^, **σ**_M_^2^ were calculated via Hamiltonian Monte Carlo sampling using the ‘greta’ R package (http://CRAN.R-project.org/package=greta) (Supplementary Methods). We used 2000 steps for warmup and 5000 steps over 4 chains to ensure convergence. Mean estimates along with their 95% credible intervals may be found in Supplementary Data 2 and 3.

Combined genotype-mutagen interactions were tested for effect in two settings: altering the total number of base substitutions, and changing the distribution of mutations. The FC in the number of single base substitutions was calculated as predicted with interactions versus the one predicted without interactions using 2000 out of 10,000 draws across 4 chains for all 196 interactions. The change in spectrum was quantified by calculating the cosine distance between the expected profiles with and without interactions, and those with distance higher than 0.2 were considered different. As all of the interactions which showed a change in signature appearance also came up in burden analysis, we only applied hypothesis testing (by testing the $\chi$^2^-statistic of the squared *z*-score of log(E[FC_*s*_]), *s* = 1, …, 196), and corrected for multiple testing using Benjamini–Hochberg FDR control procedure.

### Batch effect control for EMS exposure

EMS exposure was the only genotoxin for which we observed very different mutation counts across different experiments in wild-type. Respective samples were coming from 5 batches, which are stated in the ‘Comments’ section of the samples description table in Supplementary Table 1. In order to account for this effect, we introduced additional factor ${\mathbf{\upxi }}$, ${\mathbf{\upxi }}$ = {$\xi$_*i*_}, *i* = 1, …, 4 which accounted for the log-difference in real dose applied to the samples in batches 2 to 5 compared to batch 1 which was considered as reference. The prior distribution for these adjustments was taken as $\xi$_*i*_ ~*N*(0, 0.5) iid. The dose for these samples was then calculated as **d**’ = **d** ∙ exp(**batch** × ${\mathbf{\upxi }}$), where **batch** $\in {\Bbb Z}_2^{2717 \times 4}$ is a binary matrix reflecting if a sample belongs to any of the batches 2–5. These adjustments were estimated along with the rest of the coefficients.

### Estimation of mutational contributions of DNA repair components

Subsequent estimates of the fraction of mutations contributed or prevented by different DNA repair components upon genotoxin exposures were acquired as $\left( {1 - \frac{1}{x}} \right) \cdot 100\%$ for positive interactions and as $\left( {1 - x} \right) \cdot 100\%$ for negative interactions, where *x* is the FC in the mutation rate induced by the respective interaction compared to the exposure in wild-type.

### TCGA/PCAWG human cancer data analysis

The susceptibility of a DNA pathway to alteration was defined as having altered expression or high impact mutations in relevant genes (Supplementary Methods, Supplementary Data 3). The interaction effect was then estimated using Hamiltonian Monte Carlo sampling^74^ for the following model for matrix of mutation counts in *N* samples with *R* mutation classes **Y** $\in {\Bbb N}_0^{R \times N}$

$$\begin{array}{l}{\mathbf{Y}}\sim {\mathrm{NB}}({\mathbf{\upmu }},\,\varphi ),\\ {\mathbf{\upmu }} = {\mathbf{S}}^{( - K)} \times {\mathbf{\upalpha }}_{( - {\mathrm{K}})} + ({\mathbf{S}}^{({\mathrm{K}})} \times {\mathbf{\upalpha }}_{({\mathrm{K}})}) \cdot {\mathbf{f}}_{\mathrm{G}},\\ {\mathbf{f}}_{\mathrm{G}} = {\mathrm{exp}}({\mathbf{X}} \times {\mathbf{\upbeta }}),\\ {\mathbf{\upalpha }}\sim {\mathrm{Unif}}\left( {0,{\mathrm{M}}} \right)^{{\mathrm{K}} \times {\mathrm{N}}},\\ {\mathbf{\upbeta }}\sim {\mathrm{N}}\left( {0,\,0.5} \right)^{{\mathrm{M}} \times {\mathrm{R}}},\end{array}$$

where ${\mathbf{\upalpha }}$ is the matrix of exposures (number of mutations assigned to each signature), **S** is a *R* × *K* matrix of *K* signatures (each column is a signature, and is normalised to sum to 1), and **f**_G_ is a *R* × *N* matrix of interaction factors which alter the signature. It consists of **X** $\in {\Bbb Z}_2^{N \times M}$—a binary matrix of labels, and **β** which is a *M* × *R* matrix of spectra of the interaction effects with a i.i.d. *N*(0, 0.5) prior. *M* is the maximal number of mutations per sample in the dataset. The overdispersion parameter $\varphi$ = 50 was chosen based on variability estimates across all cancer samples. For details on sample selection, analysis of expression, mutations and selective pressure across DNA repair genes, see Supplementary Methods, Supplementary Notes 3 and 4 and Supplementary Data 3 and 5.

### Reporting summary

Further information on research design is available in the Nature Research Reporting Summary linked to this article.

## Supplementary information

Supplementary Information

Peer Review File

Description of Additional Supplementary Files

Supplementary Data 1

Supplementary Data 2

Supplementary Data 3

Supplementary Data 4

Supplementary Data 5

Supplementary Data 6

Reporting Summary

## Data Availability

Sequencing data are available under ENA Study Accession Numbers ERP000975 [https://www.ebi.ac.uk/ena/data/search?query=ERP000975] and ERP004086 [https://www.ebi.ac.uk/ena/data/search?query=ERP004086]. Mutation counts underlying the plots shown in Figs. 1a, b, 3b, 4b, 5b, c, Supplementary Figs. 4a, b, 5a, b, 6a, b are provided as Supplementary Data 1. Signature and interaction-related fold change estimates from the model (used in Figs. 1c, d, 2, 3b, 4b, 5b, c, Supplementary Figs. 2, 4–8) are supplied as Supplementary Data 2 and 4. Filtered VCF files for each sample are available in Supplementary Data 6. Annotated Mutect somatic variant calls, gene expression and DNA methylation data for TCGA samples are publicly available from NCI GDC (https://gdc.cancer.gov^60^) and supplementary materials from the respective studies^34,62–66^. A set of more accurately filtered SNV and indel calls for TCGA samples used for mutational signature analysis were used^56^. Please refer to Supplementary Table 2 for the links to all sources used for human data analysis. For more details on sample selection, see Supplementary Methods. Whole genome sequencing data of cSCCs from XP and non-XP patients^53^ is stored in dbGaP database as controlled access data under accession code phs000830.v1.p1 [https://www.ncbi.nlm.nih.gov/projects/gap/cgi-bin/study.cgi?study_id=phs000830.v1.p1]. The remaining data are available in the Article, Supplementary files or available from the author upon reasonable request.

## Code availability

Codes for most downstream analyses and figures can be accessed from http://github.com/gerstung-lab/signature-interactions. Raw data processing and variant calling was performed via Sanger Institute Cancer IT pipeline using bwa v. 0.5.9, samtools v. 0.1.18, CaVEMan v. 1.5.3, PINDEL v. 0.3.5, DELLY v. 0.7.8^59,67–69,72^.
