# Supplementary material for: Mutational signatures are jointly shaped by DNA damage and repair
Source: Nat Commun. 2020 May 1;11:2169. doi: 10.1038/s41467-020-15912-7 (PMC7195458; doi:10.1038/s41467-020-15912-7)
Supplement: Supplementary file 1 — Supplementary Information [file 41467_2020_15912_MOESM1_ESM.pdf]

# Supplementary Information

## Mutational signatures are jointly shaped by DNA damage and repair

Nadezda V Volkova, Bettina Meier, Víctor González-Huici, Simone Bertolini, Santiago Gonzalez, Harald Voeringer, Federico Abascal, Iñigo Martincorena, Peter J Campbell, Anton Gartner and Moritz Gerstung

# Table of Contents

**Supplementary Figures** 4

Supplementary Figure 1. Somatic mutations and silencing of DNA repair pathway genes in human cancers. 5

Supplementary Figure 2. Experimental mutagen signatures in C. elegans and their comparison to human signatures. 6

Supplementary Figure 3. Interaction between DNA damage and repair in human cancer. 7

Supplementary Figure 4. Selected genotoxin-repair interactions for alkylating agents in C. elegans. 9

Supplementary Figure 5. Features of TLS polymerase mutants and analysis of deletions in C. elegans. 10

Supplementary Figure 6. Selected C. elegans genotoxin-repair interactions involving NER mutants. 11

**Supplementary Notes** 12

Supplementary Note 1. Mutational signatures of DNA repair deficiencies in C. elegans 12

Supplementary Note 2. List of significant interaction effects between genotoxins and DNA repair deficiencies in C. elegans 23

Supplementary Note 3. Frequency of biallelic DNA repair deficiency and selective pressure on DNA repair genes measured across TCGA 40

Supplementary Note 4. Tissue-specific effects of MMR deficiency 41

**Supplementary Tables** 42

Supplementary Table 1. Strains used in the study. 42

Supplementary Table 2. TCGA data sources. 44

**Supplementary Methods** 45

1. Comparison between C. elegans, iPS cell and cancer derived signatures of genotoxins 45

2. Microhomology analysis for deletion breakpoints in C. elegans 46

3. DNA repair analysis across TCGA 46

3.1 Motivation 46

3.2. Data description 47

3.3. Classification of mutations across pathways and labeling of samples: monoallelic and biallelic deficiency 47

3.4. Analysis of mutational burden and change in the mutational profile 48

3.4.1 Mutation burden 48

3.4.2 Mutational profiles 48

4. dNdS analysis across DNA repair genes in TCGA 48

4.1. Sample selection 48

4.2. Pathway-level analysis 49

4.3. Gene-level analysis 49

5. Interaction analysis in human data 49

5.1 Damage-repair interactions in cancer: motivation 49

5.2 Model specification 49
5.3. POLE proofreading domain mutations and MMR deficiency 50
5.4. Tissue-specific effects in MMR deficiency 50
5.5. Temozolomide and O-6-methylguanine-DNA methyltransferase 51
5.6. APOBEC mutagenesis and REV1/UNG defects 51
5.7. UV exposure and xeroderma pigmentosum (XP) patients 51
5.8. UV exposure and somatic defects NER 51
5.9. Smoking and NER defects 52

**Supplementary References 53**

# Supplementary Figures

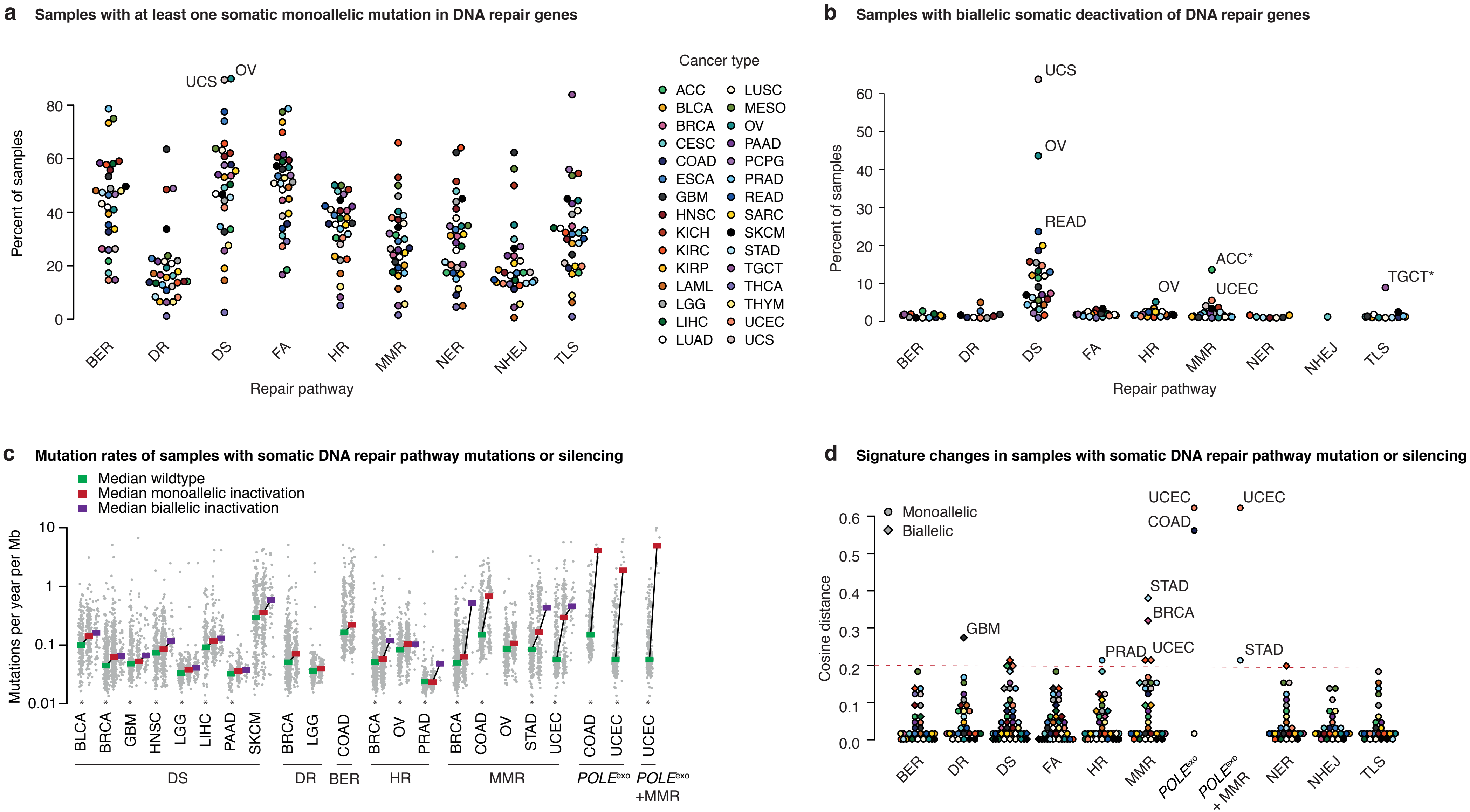


**Sup. Figure 1**. Somatic mutations and silencing of DNA repair pathway genes in human cancers

# Supplementary Figure 1. Somatic mutations and silencing of DNA repair pathway genes in human cancers.

a. Percentages of samples with mono- or biallelic defects in core components of the indicated DNA repair pathways by cancer type for 9,948 cancers from TCGA. Raw data and details on cancer types are described in **Supplementary Data 3**.
b. Percentages of samples with biallelic inactivation of core components of the indicated DNA repair pathway by cancer type. Raw data are described in **Supplementary Data 3**.
c. Age-adjusted mutation rates in samples without (green) or with mono- (red) or biallelic (purple) DNA repair pathway deficiency. Grey dots represent mutation rates per year per Megabase for individual tumour samples, coloured bars denote the medians in each group. Only DNA repair deficiencies with one-sided Wilcoxon rank sum test FDR under 5% (indicating increased mutation rates) are shown in the tumour type in which they occur. Stars denote cancer types where the presence of damaging mutations in the pathway is unlikely to be explained just by the increase in mutational burden (see **Supplementary Methods**).
d. Median mutational signature changes (measured as 1 - cosine similarity) of samples with DNA repair pathway deficiencies with respect to median wild-type signatures. Colours indicate tumour types as shown in **a.**, the red dotted line denotes the cutoff of 0.2 (corresponding to cosine similarity of 0.8) above which signatures are not considered similar.

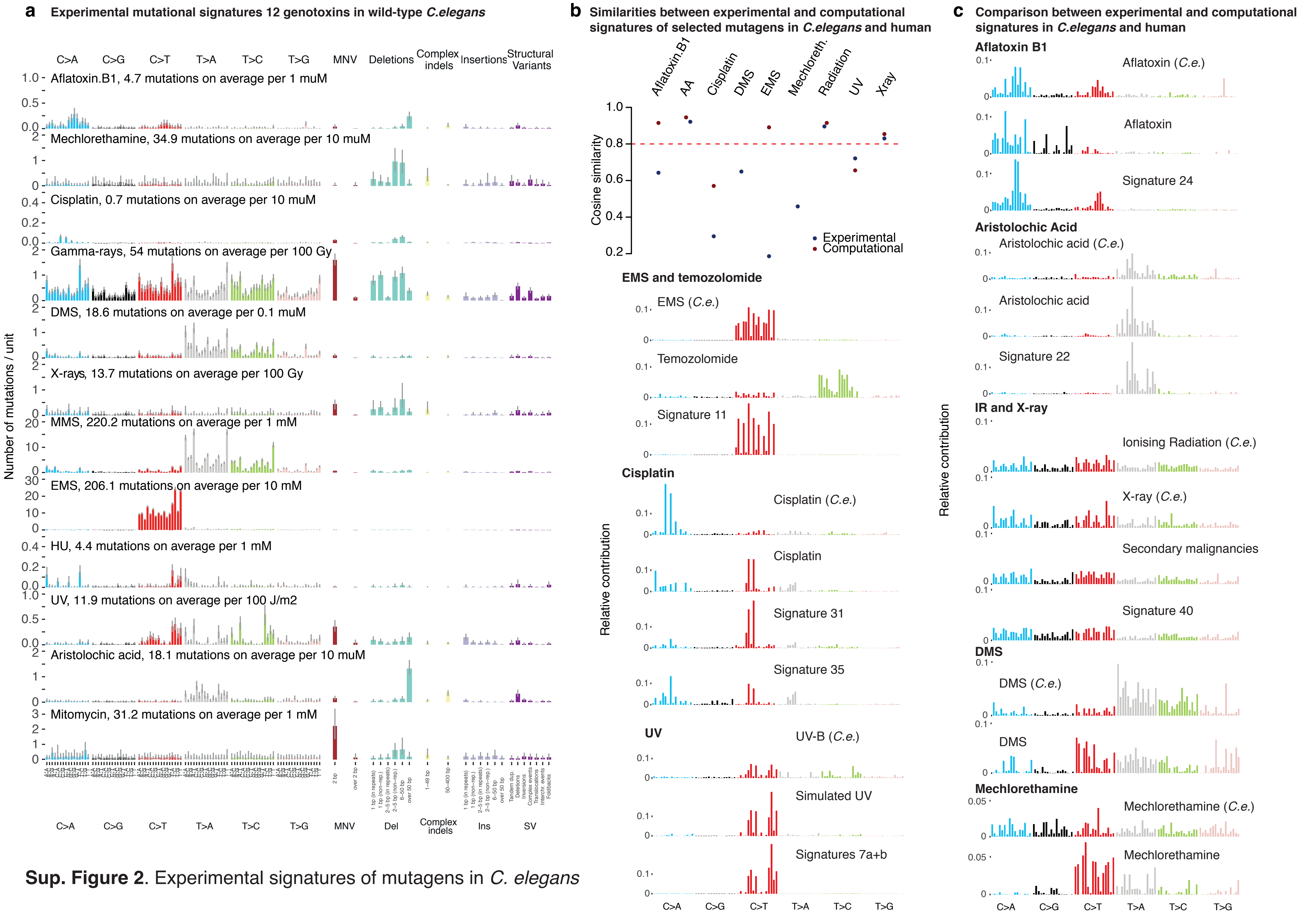


**Sup. Figure 2**. Experimental signatures of mutagens in *C. elegans*

# Supplementary Figure 2. Experimental mutagen signatures in *C. elegans* and their comparison to human signatures.

a. Mutational signatures extracted for 12 genotoxins in *C. elegans* wild-type. Each barplot reflects the mean number of mutations per average mutagen dose, headers contain the estimate for the total number of mutations per average dose, error bars represent 95% credible intervals. Layout as in **Figure 1d**. For details on doses and mutagen specification, see **Supplementary Data 1**. For precise mutation numbers per unit in each mutation class, see **Supplementary Data 2**.

b. Cosine similarities between the humanized *C. elegans* signatures and experimental[1] (blue) and computational[2] (red) signatures of the same or related mutagens in human cells or cancers. *C. elegans* signatures were adjusted to the human genome trinucleotide frequency. Experimental signature for ionizing radiation was estimated as an averaged spectrum of 12 radiotherapy-associated secondary malignancies from [3]. No counterparts from human data were found for *C. elegans* HU, Mitomycin C and MMS spectra. Red line denotes the cut-off of 0.8 above which the spectra are considered similar.

c. Comparison between experimental and computational signatures for selected mutagens in *C. elegans* and human. Mutational profiles are shown as the relative contribution of mutations across the 96 base substitution classes. *C. elegans* signatures are indicated with *C.e*.

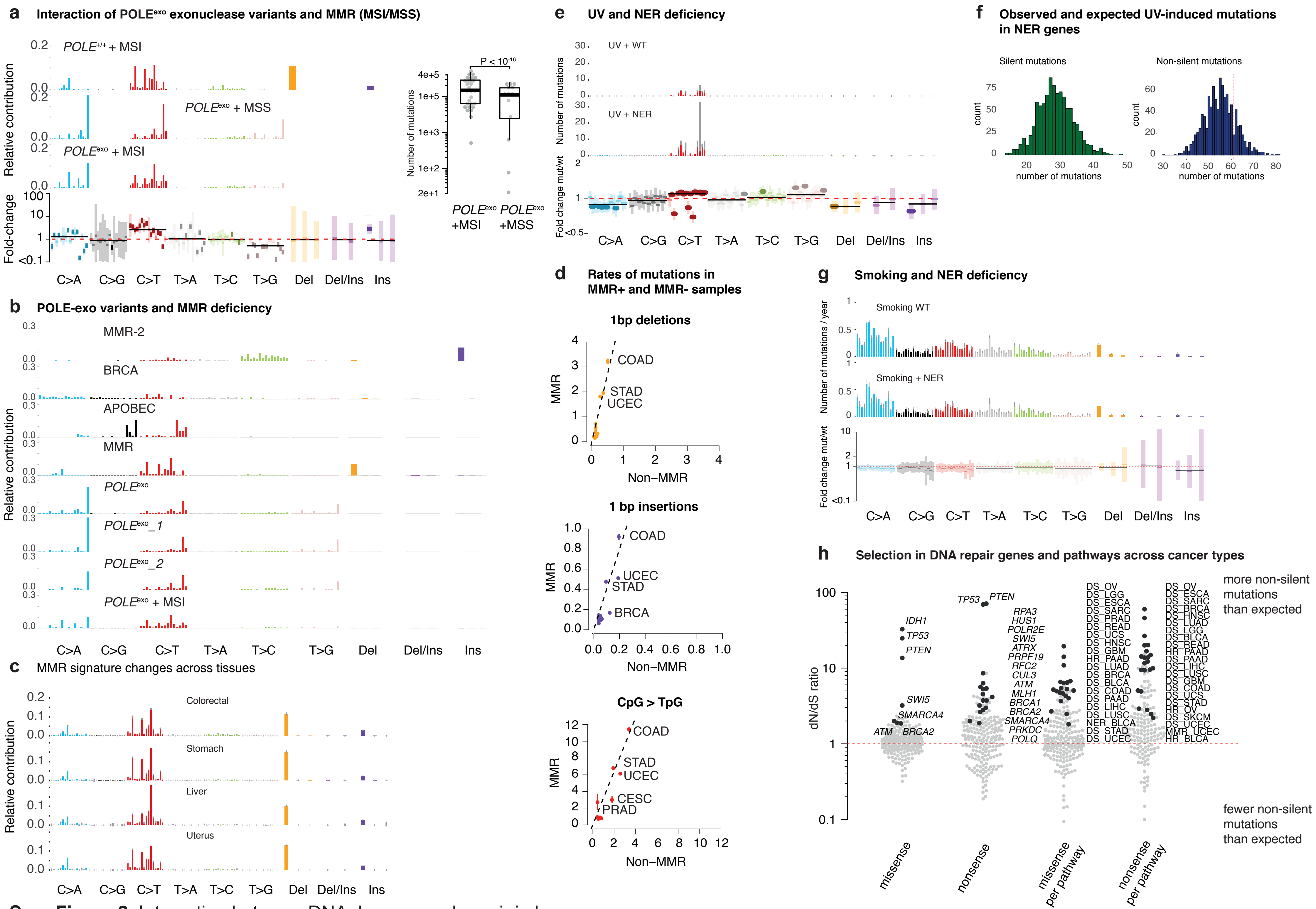


**Sup. Figure 3**. Interaction between DNA damage and repair in human cancer

# Supplementary Figure 3. Interaction between DNA damage and repair in human cancer.

a. Left: Mutational signatures of MMR deficiency as indicated by microsatellite instability (MSI), POLE deficiency, and combined POLE and MMR deficiency. Bottom: fold-changes per mutation class between the average $POLE^{exo}$+ microsatellite stable (MSS) and $POLE^{exo}$+ MSI signatures, along with 95% credible intervals. Right: Mutation burden of POLE exonuclease deficiency in MMR proficient (MSS, n=40) and deficient (MSI, n=15) uterine cancers. Boxes denote the interquartile range (IQR, 25% percentile - 75% percentile), thick lines the median, whiskers - 1.5x the IQR below the first quartile and above the third quartile. MMR deficiency is a significant predictor (p-value < $10^{-16}$) of mutation burden in $POLE^{exo}$ deficient samples in a Poisson GLM (burden ~ MMR status).

b. 5 mutational signatures extracted from uterine cancers (see **Supplementary Methods**), and the POLE exonuclease variant signature with effects of different $POLE^{exo}$ hotspot mutations (P286R and V411L) and interaction with MMR deficiency ($POLE^{exo}$ + MSI).

c. Changes of MMR deficiency signatures across different tissues. Each barplot represents the appearance of the most widespread MMR signature in the respective tissue.

d. Changes in mutation rates between MMR proficient (Non-MMR) and deficient (MMR) cancers represented by per-year rates for 1-bp deletions (top), 1-bp insertions (middle) and CpG>TpG mutations (bottom) across individual cancer types. Dotted lines represent the linear slope fitted to the rates of MMR deficient versus proficient samples.

e. Average mutational signatures of UV light for melanoma samples proficient (WT, top panel) and deficient (NER, lower panel) for NER expressed in mutations/year shown with their 95% credible intervals. The bottom panel shows the fold-change per mutation class. Layout as in **Figure 3d**.

f. Simulated and observed numbers of UV-induced silent and non-silent mutations in NER genes in skin cutaneous melanoma (TCGA SKCM) samples with high prevalence of signature 7.

g. Average mutational signatures of tobacco smoking in lung cancer samples proficient (WT, top panel) and deficient (NER, lower panel) for NER shown with 95% credible intervals. The bottom panel depicts the fold-change per mutation class. Layout as in **Figure 3d**.

h. Pan-cancer dN/dS values for missense and nonsense mutations in 248 DNA repair-associated genes, and dN/dS ratios across 30 cancer types for 9 DNA repair pathways (aggregating all genes per pathway). The dN/dS ratio measures the excess of observed non-silent mutations relative to the expected number based on observed silent mutations (1 signifies neutrality, > 1 indicates a cancer-promoting effect). Black dots reflect results which reached significance compared to the background (FDR 10% within the respective group), with respective genes or DNA repair pathways indicated (see **Supplementary Methods**). For details, see **Supplementary Data 5**.

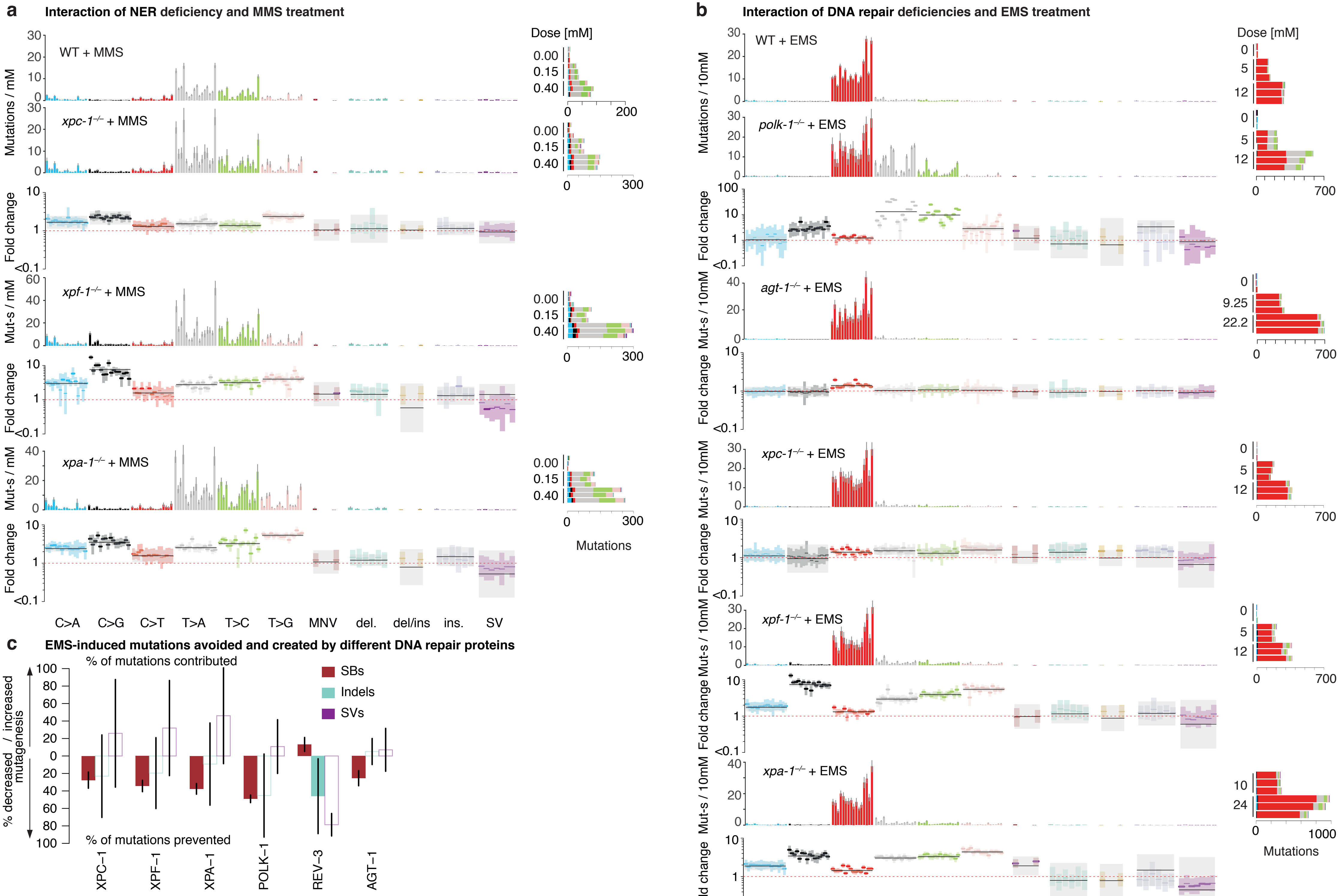


**Sup. Figure 4.** Selected genotoxin-repair interactions for alkylating agents in *C. elegans*.

# Supplementary Figure 4. Selected genotoxin-repair interactions for alkylating agents in *C. elegans*.

a. Average MMS-induced mutational signatures in wild-type (WT) and NER mutants, their fold-changes, and mutation counts per replicate for different doses of MMS. Layout as in **Figure 3b**.
b. Average EMS-induced mutational signatures in wild-type, *polk-1*$^{-/-}$, *agt-1*$^{-/-}$ and NER mutants, their fold-changes, and mutation counts per replicate for different doses of EMS. Layout as in **Figure 3b**.
c. Estimated mean fractions of mutations contributed or prevented by different DNA repair components compared to the mutations observed upon EMS exposure in wild-type *C. elegans.* Error bars (black) represent 95% confidence intervals, bars filled with colour indicate contributions significantly different from zero (chi-squared test, FDR 5% in each category), while the ones with no fill colour reflect non-significant ones. Layout as in **Figure 3c**.

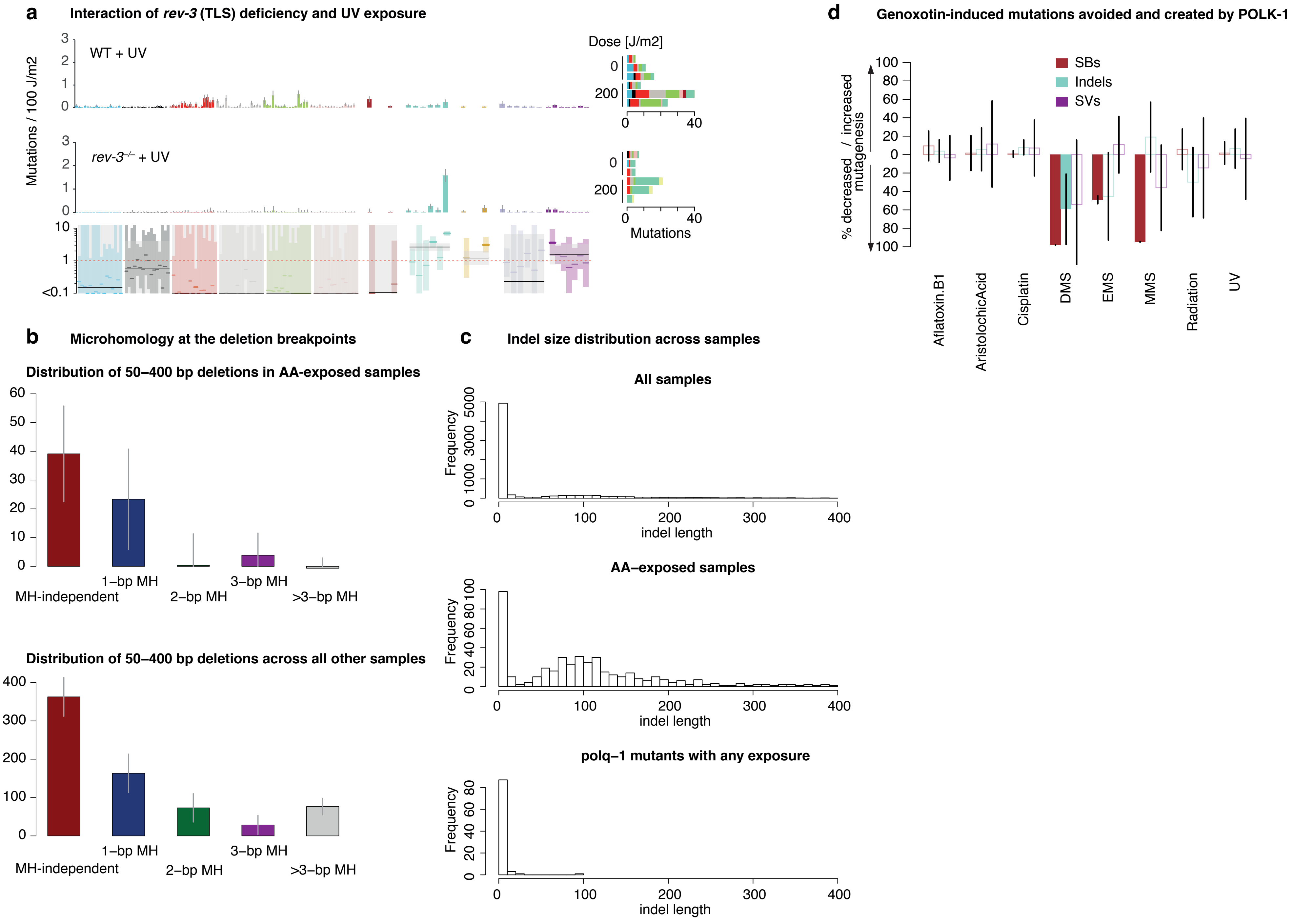


**Sup. Figure 5**. Features of TLS polymerase knock-outs and analysis of deletions in *C.elegans*

## Supplementary Figure 5. Features of TLS polymerase mutants and analysis of deletions in *C. elegans.*

a. Average UV-induced mutational signatures in wild-type and *rev-3* (TLS) deficient mutants, their fold-changes per mutation class, and the mutation counts per replicate for different doses of MMS. Layout as in **Figure 3b**.

b. Distribution of actual microhomology sizes at the breakpoints of 50-400 bp deletions estimated in AA-exposed samples (top, n=232 deletions in total across 72 samples) and across all other samples (bottom, n=381 deletions in total across 2645 samples). Each bar represents the estimated average number of 50-400 bp deletions with different microhomology sizes (MH-independent - green bar, 1-bp MH - blue, 2-bp MH - purple, and >3-bp MH - grey) given the possibility of having a deletion with a longer MH by chance. Error bars denote 95% confidence intervals. The majority of deletions likely occurred in a MH-independent manner (red bars). The contribution of deletions with 1-bp MH was substantial in both cases (blue bar), but dominated the spectrum of deletions with MHs in AA-exposed samples. A high level of short MH indicates the activity of TMEJ contributing to the generation of such deletions across all samples, with an excess of 1-bp MH occuring in AA-exposed samples.

c. Frequency of indels by indel length across all samples in the dataset (top, n=24308 indels across all 2717 samples), aristolochic acid exposed samples (center, n=426 indels across 72 samples), and *polq-1* deficient mutants (bottom, n=92 indels across 95 samples).

d. Estimated mean fractions of mutations contributed or prevented by POLK-1 compared to the mutations observed upon exposure to different genotoxins in wild-type *C. elegans*. Error bars denote 95% confidence intervals. Bars with no fill

colour reflect non-significant changes (chi-squared test FDR 5% in each category).

Layout as in **Figure 3c**.

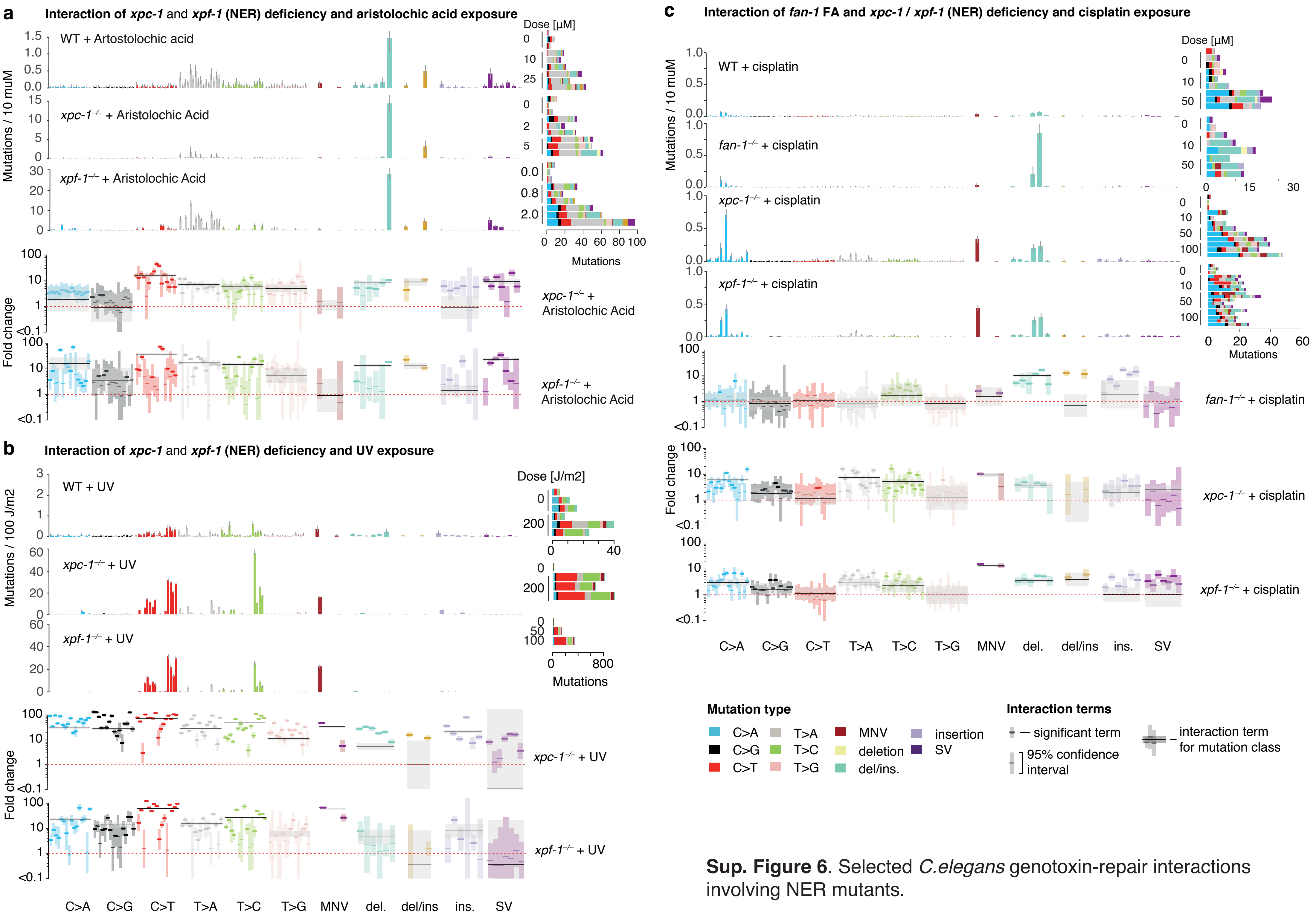


**Sup. Figure 6**. Selected *C.elegans* genotoxin-repair interactions involving NER mutants.

# Supplementary Figure 6. Selected *C. elegans* genotoxin-repair interactions involving NER mutants.

a. Average aristolochic acid (AA) induced mutational signatures in wild-type and NER (*xpc-1* and *xpf-1*) mutants with error bars depicting 95% credible intervals, their fold-changes per mutation class, and the mutation counts per replicate for different doses of AA. Layout as in **Figure 3b**. Note that scale bars are different in the center and lower panels.
b. Average UV-induced mutational signatures in wild-type and NER (*xpc-1* and *xpf-1*) mutants with error bars depicting 95% credible intervals, their fold-changes per mutation class , and the mutation counts per replicate for different doses of UV. Layout as in **Figure 3b**. Note that scale bars are different in the upper and lower panels.
c. Average cisplatin-induced mutational signatures in wild-type, FA (*fan-1*) and NER (*xpc-1* and *xpf-1*) mutants with error bars depicting 95% credible intervals, their fold-changes per mutation class, and the mutation counts per replicate for different doses of UV. Layout as in **Figure 3b**.

# Supplementary Notes

## Supplementary Note 1. Mutational signatures of DNA repair deficiencies in *C. elegans*

Statistical model described in **Methods** allowed us to extract an individual mutational signature for each genetic background, along with the average mutation rates per generation. These experimental mutational signatures extracted for wild-type and 53 DNA repair deficient *C. elegans* strains are shown below (**Supplementary Figure 7).**

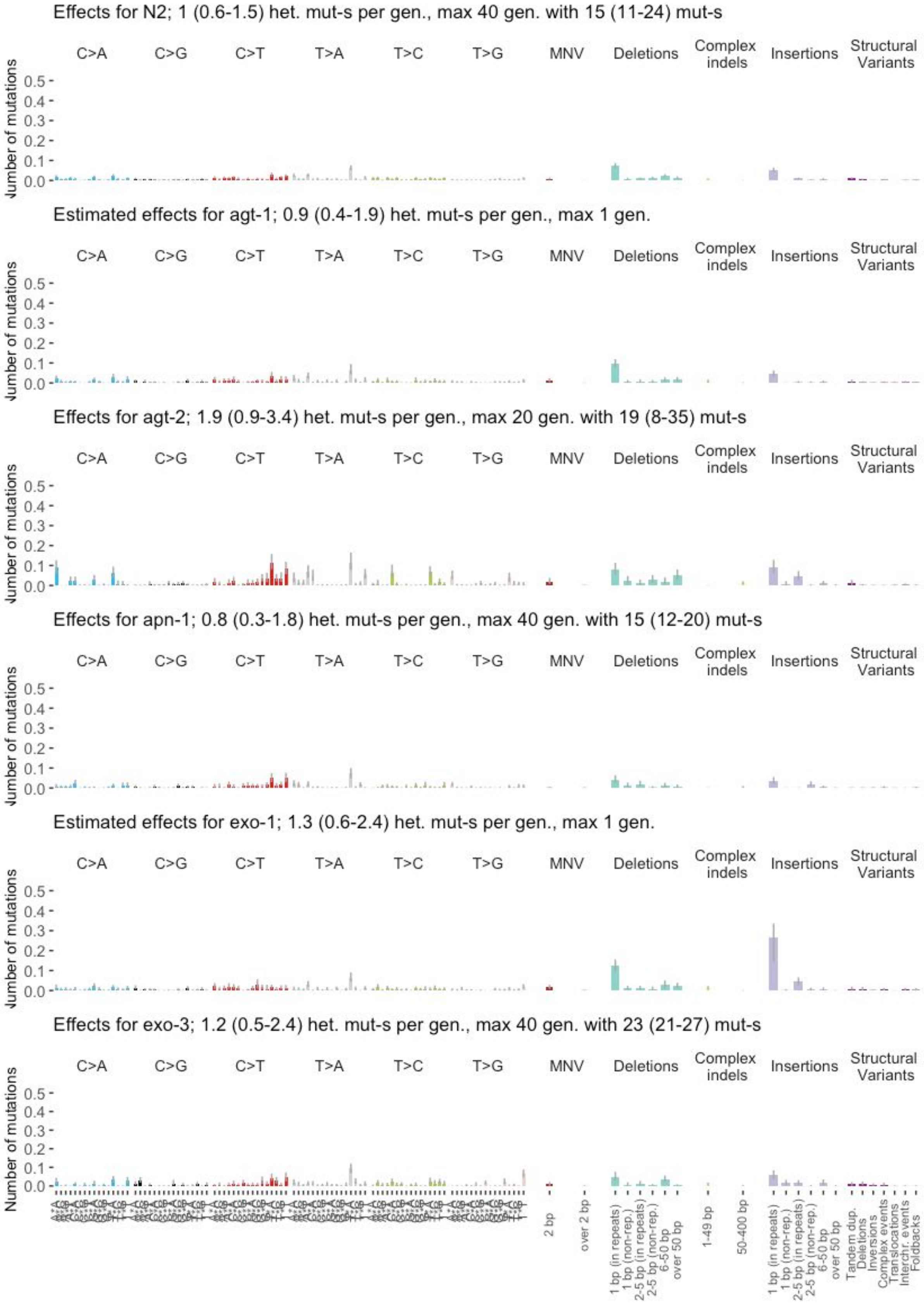

Effects for N2; 1 (0.6-1.5) het. mut-s per gen., max 40 gen. with 15 (11-24) mut-s
Estimated effects for agt-1; 0.9 (0.4-1.9) het. mut-s per gen., max 1 gen.
Effects for agt-2; 1.9 (0.9-3.4) het. mut-s per gen., max 20 gen. with 19 (8-35) mut-s
Effects for apn-1; 0.8 (0.3-1.8) het. mut-s per gen., max 40 gen. with 15 (12-20) mut-s
Estimated effects for exo-1; 1.3 (0.6-2.4) het. mut-s per gen., max 1 gen.
Effects for exo-3; 1.2 (0.5-2.4) het. mut-s per gen., max 40 gen. with 23 (21-27) mut-s
C>A
C>G
C>T
T>A
T>C
T>G
MNV
Deletions
Complex indels
Insertions
Structural Variants
Number of mutations
0.0
0.1
0.2
0.3
0.4
0.5
2 bp
over 2 bp
1 bp (in repeats)
1 bp (non-rep.)
2-5 bp (in repeats)
2-5 bp (non-rep.)
6-50 bp
over 50 bp
1-49 bp
50-400 bp
1 bp (in repeats)
1 bp (non-rep.)
2-5 bp (in repeats)
2-5 bp (non-rep.)
6-50 bp
over 50 bp
Tandem dup.
Deletions
Inversions
Complex events
Translocations
Interchr. events
Foldbacks

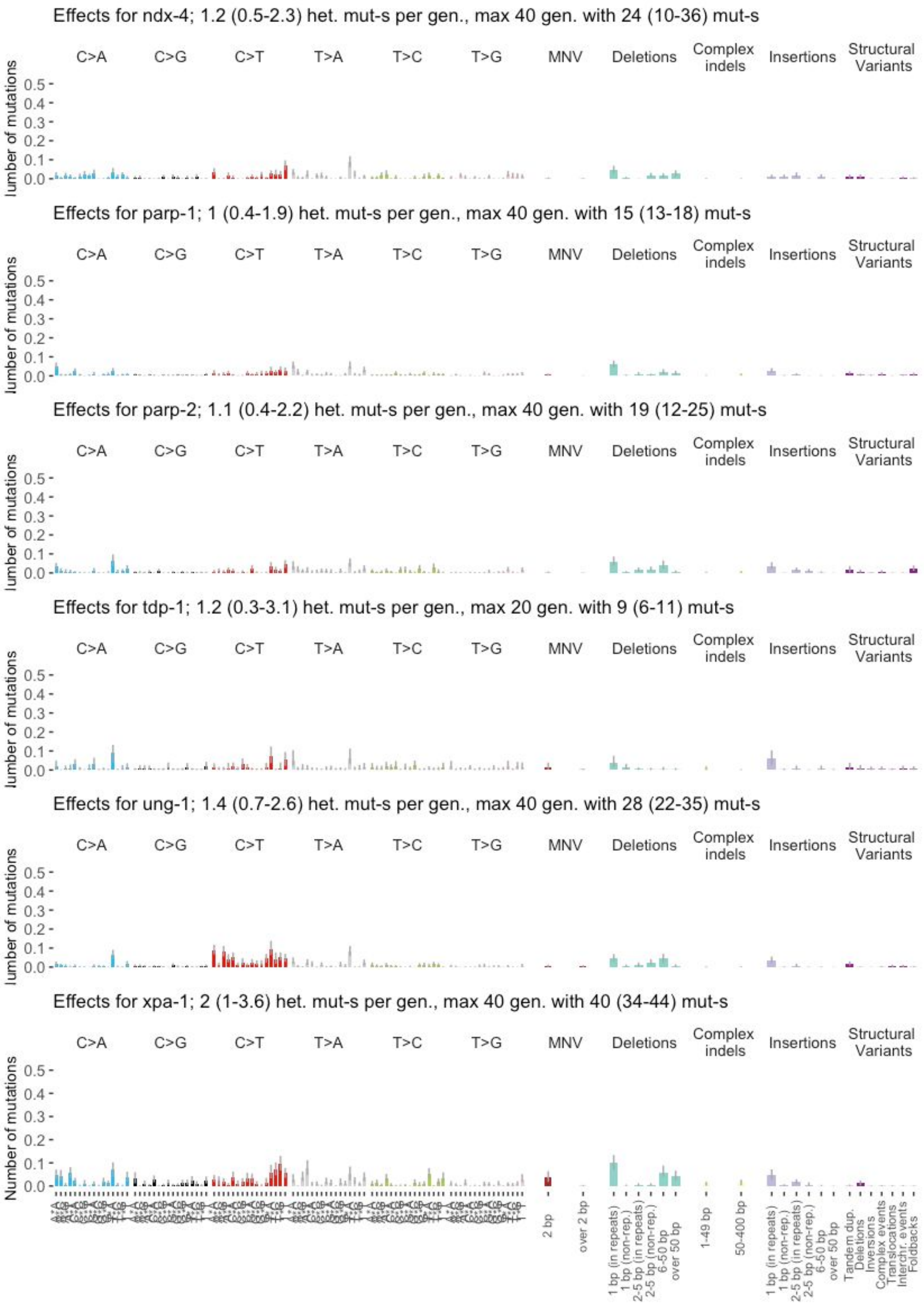
Effects for ndx-4; 1.2 (0.5-2.3) het. mut-s per gen., max 40 gen. with 24 (10-36) mut-s
Effects for parp-1; 1 (0.4-1.9) het. mut-s per gen., max 40 gen. with 15 (13-18) mut-s
Effects for parp-2; 1.1 (0.4-2.2) het. mut-s per gen., max 40 gen. with 19 (12-25) mut-s
Effects for tdp-1; 1.2 (0.3-3.1) het. mut-s per gen., max 20 gen. with 9 (6-11) mut-s
Effects for ung-1; 1.4 (0.7-2.6) het. mut-s per gen., max 40 gen. with 28 (22-35) mut-s
Effects for xpa-1; 2 (1-3.6) het. mut-s per gen., max 40 gen. with 40 (34-44) mut-s
C>A
C>G
C>T
T>A
T>C
T>G
MNV
Deletions
Complex indels
Insertions
Structural Variants
Number of mutations
0.0
0.1
0.2
0.3
0.4
0.5
2 bp
over 2 bp
1 bp (in repeats)
1 bp (non-rep.)
2-5 bp (in repeats)
2-5 bp (non-rep.)
6-50 bp
over 50 bp
1-49 bp
50-400 bp
1 bp (in repeats)
1 bp (non-rep.)
2-5 bp (in repeats)
2-5 bp (non-rep.)
6-50 bp
over 50 bp
Tandem dup.
Deletions
Inversions
Complex events
Translocations
Interchr. events
Foldbacks

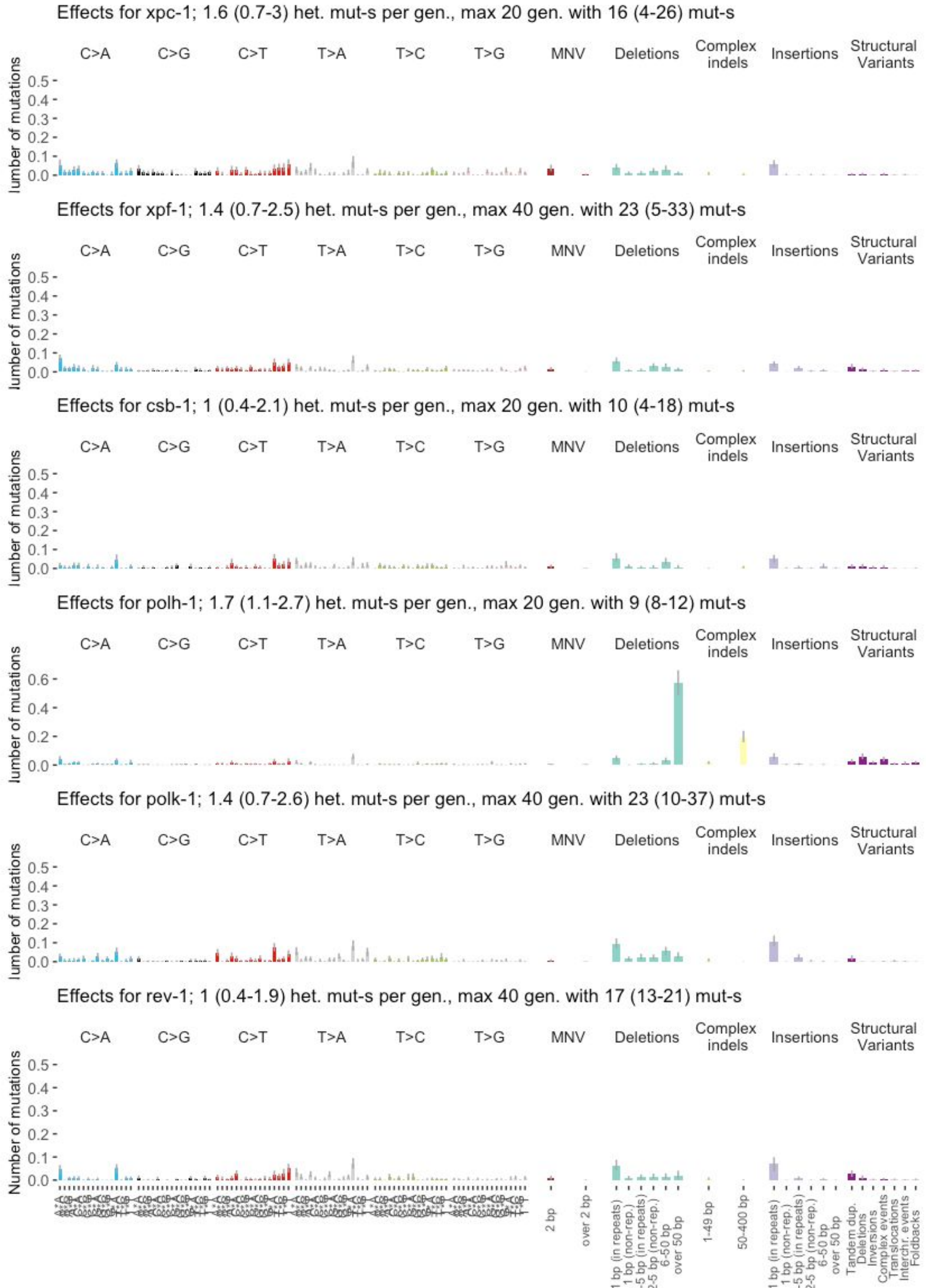
Effects for xpc-1; 1.6 (0.7-3) het. mut-s per gen., max 20 gen. with 16 (4-26) mut-s
Effects for xpf-1; 1.4 (0.7-2.5) het. mut-s per gen., max 40 gen. with 23 (5-33) mut-s
Effects for csb-1; 1 (0.4-2.1) het. mut-s per gen., max 20 gen. with 10 (4-18) mut-s
Effects for polh-1; 1.7 (1.1-2.7) het. mut-s per gen., max 20 gen. with 9 (8-12) mut-s
Effects for polk-1; 1.4 (0.7-2.6) het. mut-s per gen., max 40 gen. with 23 (10-37) mut-s
Effects for rev-1; 1 (0.4-1.9) het. mut-s per gen., max 40 gen. with 17 (13-21) mut-s
C>A
C>G
C>T
T>A
T>C
T>G
MNV
Deletions
Complex indels
Insertions
Structural Variants
Number of mutations
0.0
0.1
0.2
0.3
0.4
0.5
0.6
2 bp
over 2 bp
1 bp (in repeats)
1 bp (non-rep.)
2-5 bp (in repeats)
2-5 bp (non-rep.)
6-50 bp
over 50 bp
1-49 bp
50-400 bp
Tandem dup.
Deletions
Inversions
Complex events
Translocations
Interchr. events
Foldbacks

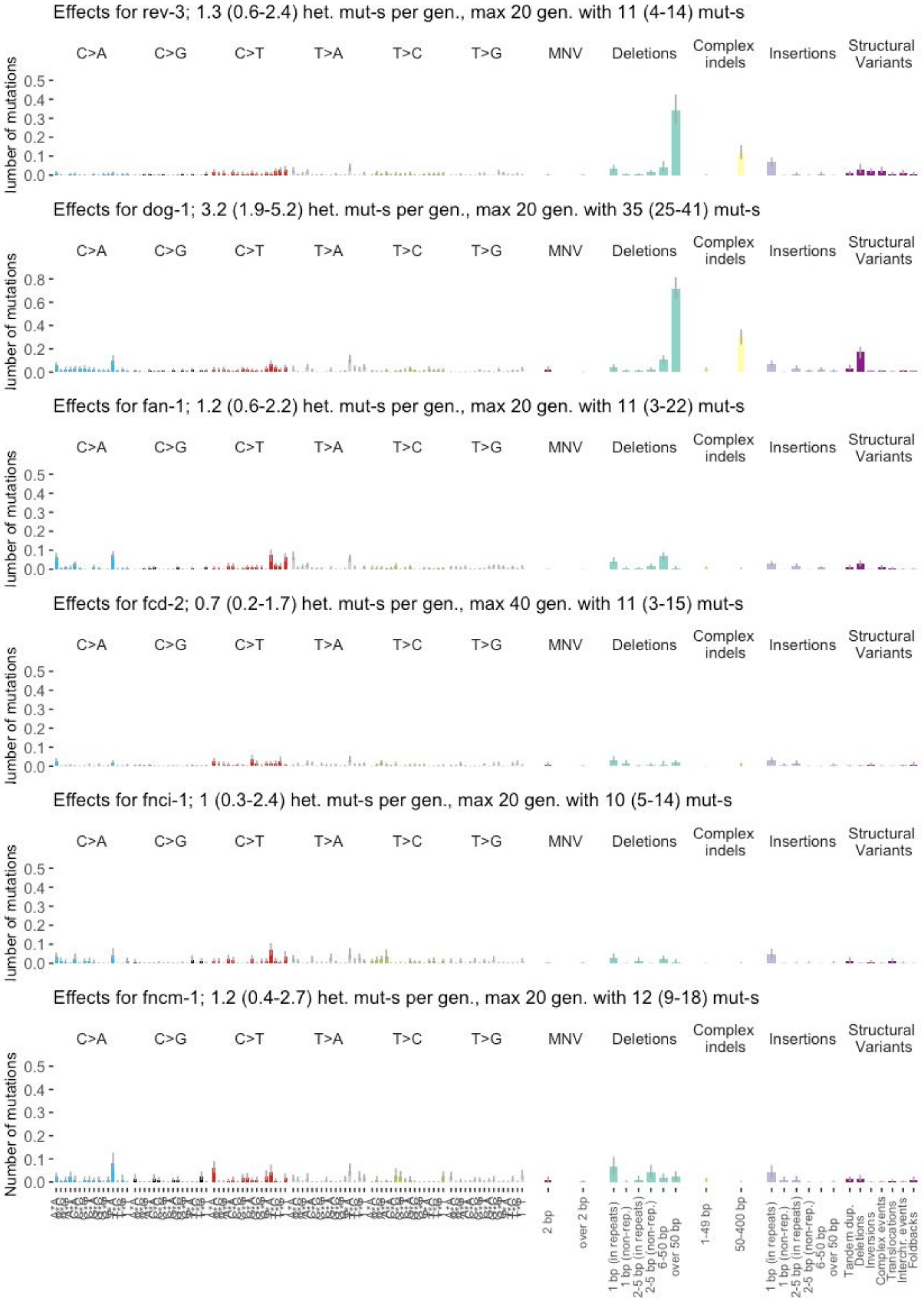
Effects for rev-3; 1.3 (0.6-2.4) het. mut-s per gen., max 20 gen. with 11 (4-14) mut-s
Effects for dog-1; 3.2 (1.9-5.2) het. mut-s per gen., max 20 gen. with 35 (25-41) mut-s
Effects for fan-1; 1.2 (0.6-2.2) het. mut-s per gen., max 20 gen. with 11 (3-22) mut-s
Effects for fcd-2; 0.7 (0.2-1.7) het. mut-s per gen., max 40 gen. with 11 (3-15) mut-s
Effects for fnci-1; 1 (0.3-2.4) het. mut-s per gen., max 20 gen. with 10 (5-14) mut-s
Effects for fncm-1; 1.2 (0.4-2.7) het. mut-s per gen., max 20 gen. with 12 (9-18) mut-s
C>A
C>G
C>T
T>A
T>C
T>G
MNV
Deletions
Complex indels
Insertions
Structural Variants
Number of mutations
2 bp
over 2 bp
1 bp (in repeats)
1 bp (non-rep.)
2-5 bp (in repeats)
2-5 bp (non-rep.)
6-50 bp
over 50 bp
1-49 bp
50-400 bp
Tandem dup.
Deletions
Inversions
Complex events
Translocations
Interchr. events
Foldbacks

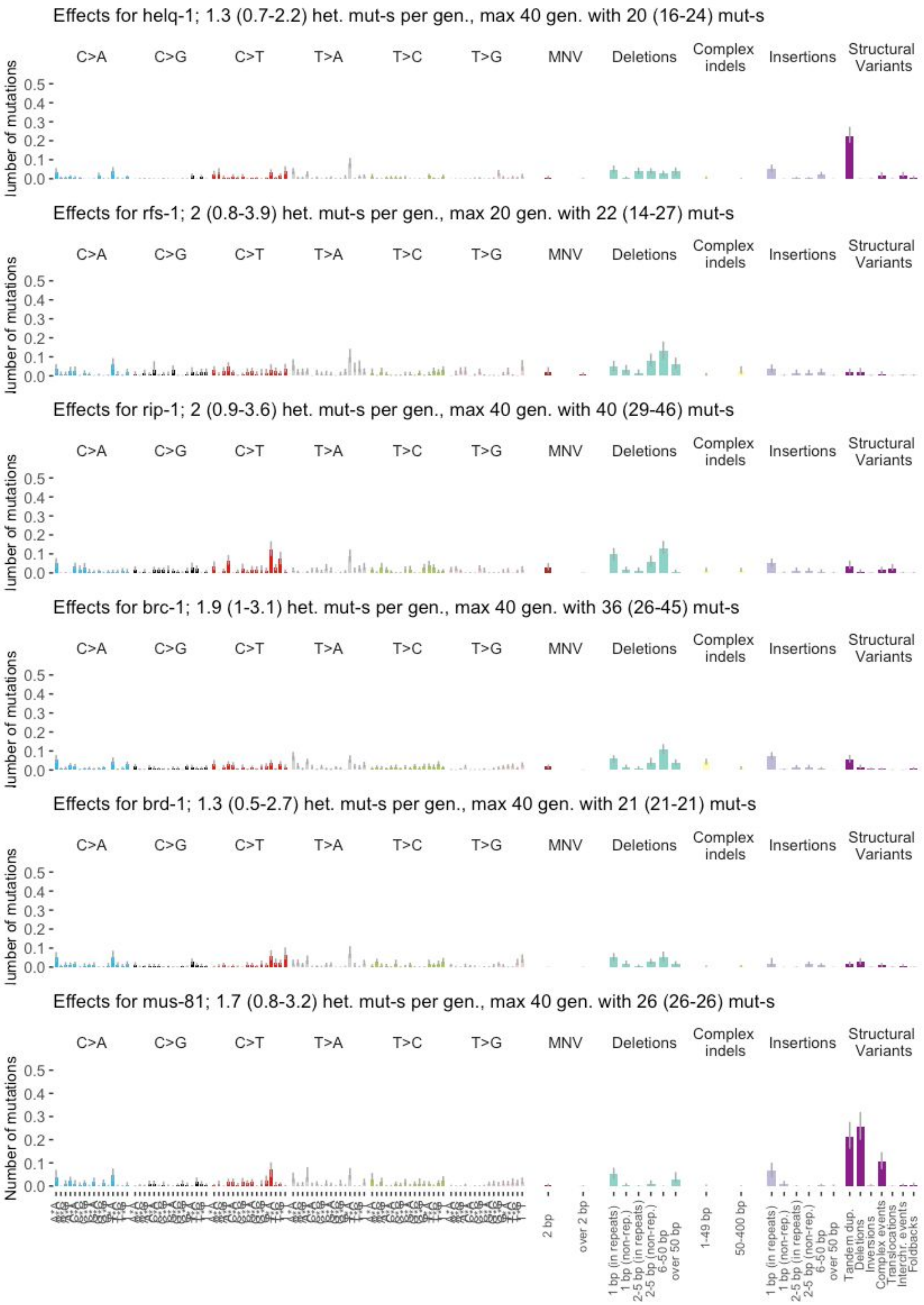
Effects for helq-1; 1.3 (0.7-2.2) het. mut-s per gen., max 40 gen. with 20 (16-24) mut-s
Effects for rfs-1; 2 (0.8-3.9) het. mut-s per gen., max 20 gen. with 22 (14-27) mut-s
Effects for rip-1; 2 (0.9-3.6) het. mut-s per gen., max 40 gen. with 40 (29-46) mut-s
Effects for brc-1; 1.9 (1-3.1) het. mut-s per gen., max 40 gen. with 36 (26-45) mut-s
Effects for brd-1; 1.3 (0.5-2.7) het. mut-s per gen., max 40 gen. with 21 (21-21) mut-s
Effects for mus-81; 1.7 (0.8-3.2) het. mut-s per gen., max 40 gen. with 26 (26-26) mut-s
C>A
C>G
C>T
T>A
T>C
T>G
MNV
Deletions
Complex indels
Insertions
Structural Variants
Number of mutations
0.0
0.1
0.2
0.3
0.4
0.5
2 bp
over 2 bp
1 bp (in repeats)
1 bp (non-rep.)
2-5 bp (in repeats)
2-5 bp (non-rep.)
6-50 bp
over 50 bp
1-49 bp
50-400 bp
1 bp (in repeats)
1 bp (non-rep.)
2-5 bp (in repeats)
2-5 bp (non-rep.)
6-50 bp
over 50 bp
Tandem dup.
Deletions
Inversions
Complex events
Translocations
Interchr. events
Foldbacks

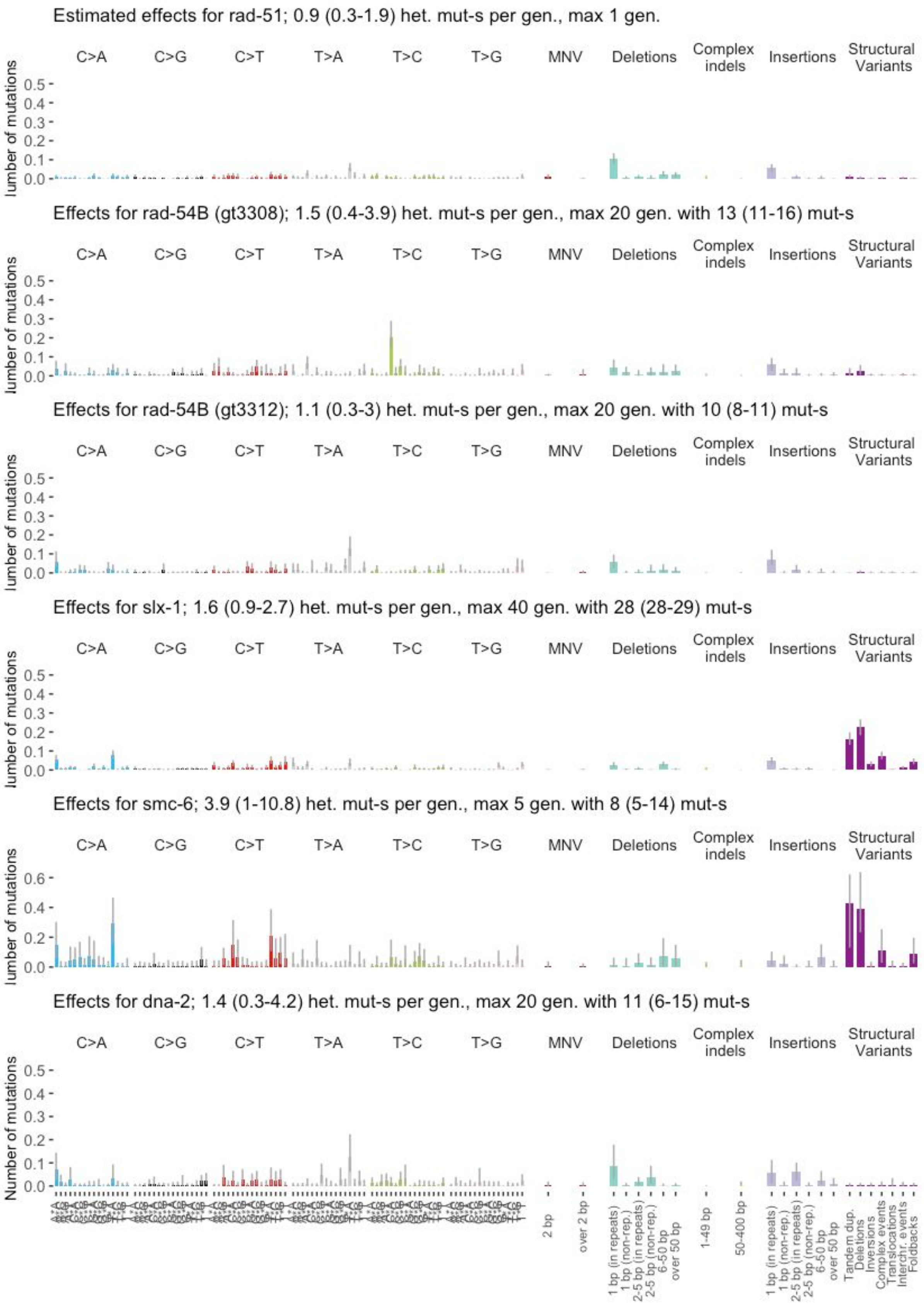
Estimated effects for rad-51; 0.9 (0.3-1.9) het. mut-s per gen., max 1 gen.
Effects for rad-54B (gt3308); 1.5 (0.4-3.9) het. mut-s per gen., max 20 gen. with 13 (11-16) mut-s
Effects for rad-54B (gt3312); 1.1 (0.3-3) het. mut-s per gen., max 20 gen. with 10 (8-11) mut-s
Effects for slx-1; 1.6 (0.9-2.7) het. mut-s per gen., max 40 gen. with 28 (28-29) mut-s
Effects for smc-6; 3.9 (1-10.8) het. mut-s per gen., max 5 gen. with 8 (5-14) mut-s
Effects for dna-2; 1.4 (0.3-4.2) het. mut-s per gen., max 20 gen. with 11 (6-15) mut-s
C>A
C>G
C>T
T>A
T>C
T>G
MNV
Deletions
Complex indels
Insertions
Structural Variants
Number of mutations
2 bp
over 2 bp
1 bp (in repeats)
1 bp (non-rep.)
2-5 bp (in repeats)
2-5 bp (non-rep.)
6-50 bp
over 50 bp
1-49 bp
50-400 bp
Tandem dup.
Deletions
Inversions
Complex events
Translocations
Interchr. events
Foldbacks

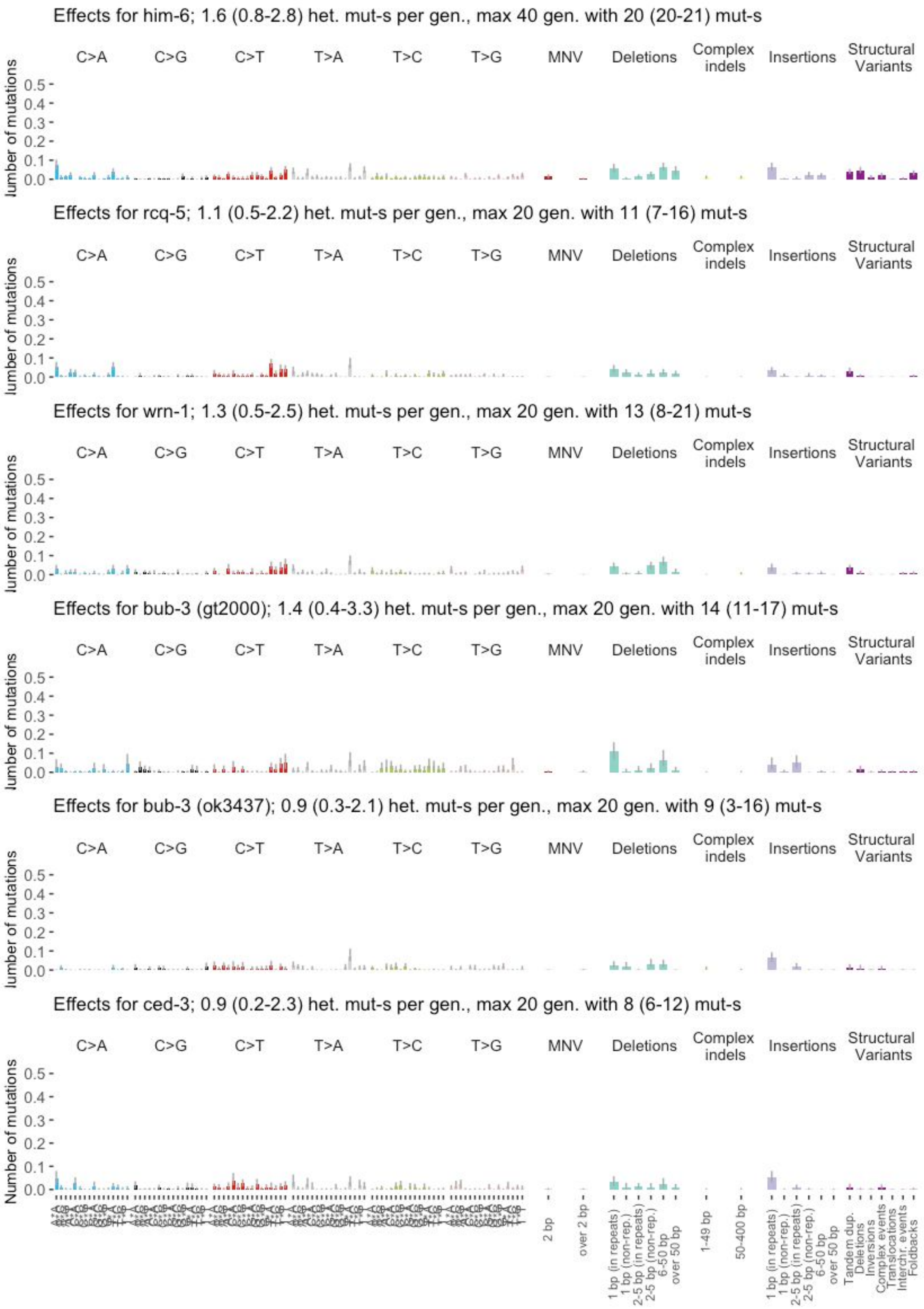
Effects for him-6; 1.6 (0.8-2.8) het. mut-s per gen., max 40 gen. with 20 (20-21) mut-s
Effects for rcq-5; 1.1 (0.5-2.2) het. mut-s per gen., max 20 gen. with 11 (7-16) mut-s
Effects for wrn-1; 1.3 (0.5-2.5) het. mut-s per gen., max 20 gen. with 13 (8-21) mut-s
Effects for bub-3 (gt2000); 1.4 (0.4-3.3) het. mut-s per gen., max 20 gen. with 14 (11-17) mut-s
Effects for bub-3 (ok3437); 0.9 (0.3-2.1) het. mut-s per gen., max 20 gen. with 9 (3-16) mut-s
Effects for ced-3; 0.9 (0.2-2.3) het. mut-s per gen., max 20 gen. with 8 (6-12) mut-s
C>A
C>G
C>T
T>A
T>C
T>G
MNV
Deletions
Complex indels
Insertions
Structural Variants
Number of mutations
0.0
0.1
0.2
0.3
0.4
0.5
2 bp
over 2 bp
1 bp (in repeats)
1 bp (non-rep.)
2-5 bp (in repeats)
2-5 bp (non-rep.)
6-50 bp
over 50 bp
1-49 bp
50-400 bp
1 bp (in repeats)
1 bp (non-rep.)
2-5 bp (in repeats)
2-5 bp (non-rep.)
6-50 bp
over 50 bp
Tandem dup.
Deletions
Inversions
Complex events
Translocations
Interchr. events
Foldbacks

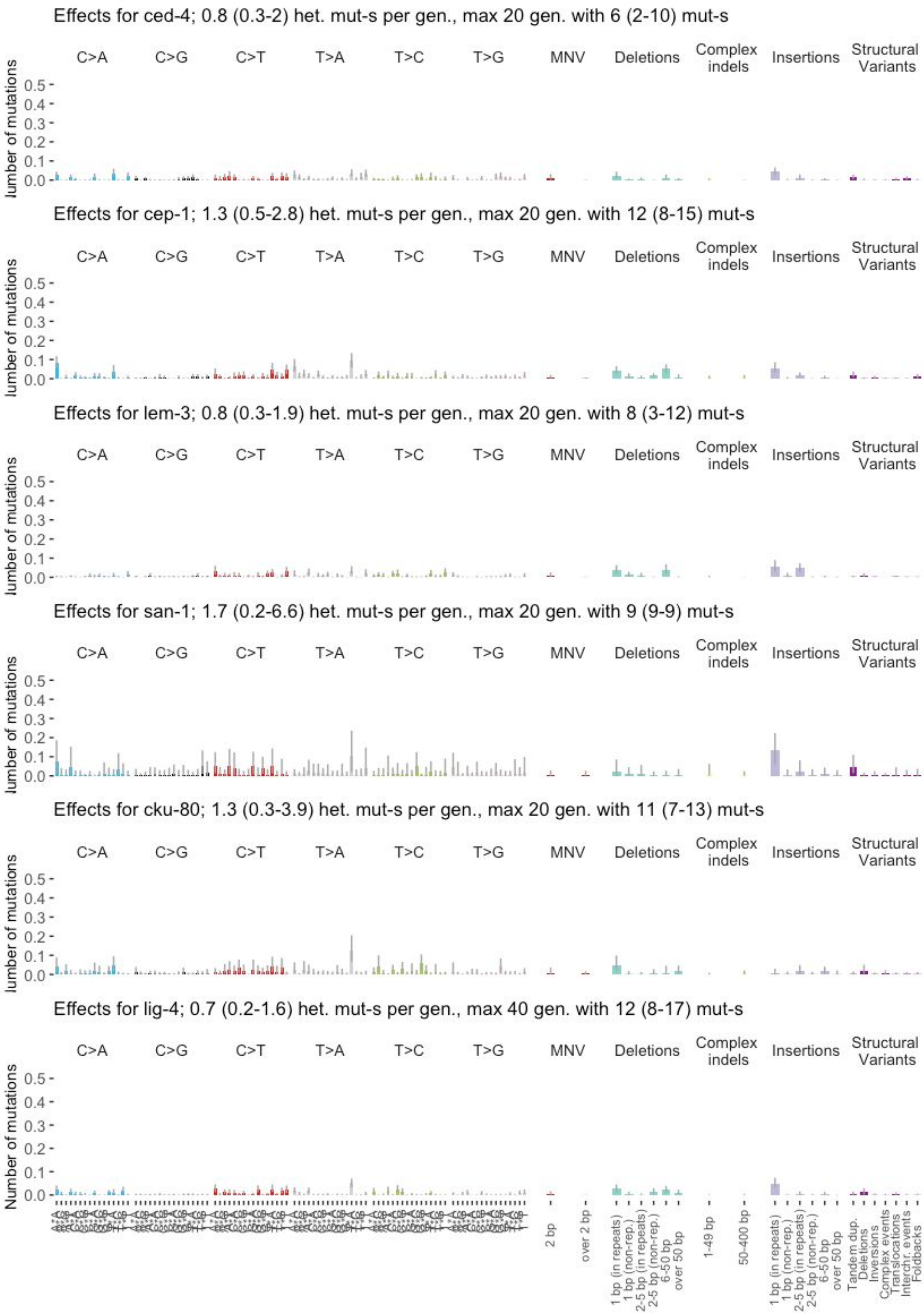

Effects for ced-4; 0.8 (0.3-2) het. mut-s per gen., max 20 gen. with 6 (2-10) mut-s
Effects for cep-1; 1.3 (0.5-2.8) het. mut-s per gen., max 20 gen. with 12 (8-15) mut-s
Effects for lem-3; 0.8 (0.3-1.9) het. mut-s per gen., max 20 gen. with 8 (3-12) mut-s
Effects for san-1; 1.7 (0.2-6.6) het. mut-s per gen., max 20 gen. with 9 (9-9) mut-s
Effects for cku-80; 1.3 (0.3-3.9) het. mut-s per gen., max 20 gen. with 11 (7-13) mut-s
Effects for lig-4; 0.7 (0.2-1.6) het. mut-s per gen., max 40 gen. with 12 (8-17) mut-s
C>A
C>G
C>T
T>A
T>C
T>G
MNV
Deletions
Complex indels
Insertions
Structural Variants
Number of mutations
0.0
0.1
0.2
0.3
0.4
0.5
2 bp
over 2 bp
1 bp (in repeats)
1 bp (non-rep.)
2-5 bp (in repeats)
2-5 bp (non-rep.)
6-50 bp
over 50 bp
1-49 bp
50-400 bp
Tandem dup.
Deletions
Inversions
Complex events
Translocations
Interchr. events
Foldbacks

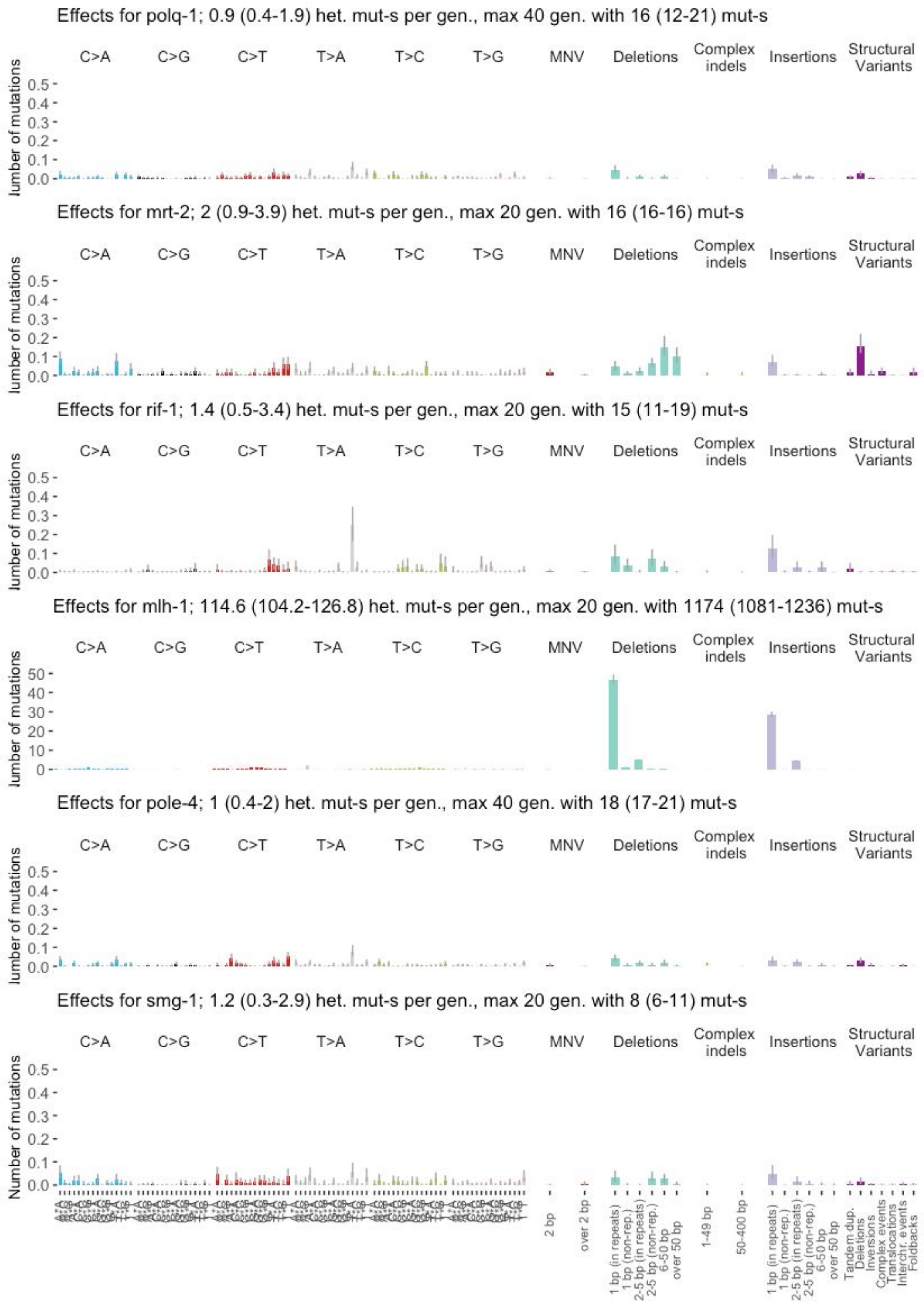
Effects for polq-1; 0.9 (0.4-1.9) het. mut-s per gen., max 40 gen. with 16 (12-21) mut-s
Effects for mrt-2; 2 (0.9-3.9) het. mut-s per gen., max 20 gen. with 16 (16-16) mut-s
Effects for rif-1; 1.4 (0.5-3.4) het. mut-s per gen., max 20 gen. with 15 (11-19) mut-s
Effects for mlh-1; 114.6 (104.2-126.8) het. mut-s per gen., max 20 gen. with 1174 (1081-1236) mut-s
Effects for pole-4; 1 (0.4-2) het. mut-s per gen., max 40 gen. with 18 (17-21) mut-s
Effects for smg-1; 1.2 (0.3-2.9) het. mut-s per gen., max 20 gen. with 8 (6-11) mut-s
C>A
C>G
C>T
T>A
T>C
T>G
MNV
Deletions
Complex indels
Insertions
Structural Variants
Number of mutations
2 bp
over 2 bp
1 bp (in repeats)
1 bp (non-rep.)
2-5 bp (in repeats)
2-5 bp (non-rep.)
6-50 bp
over 50 bp
1-49 bp
50-400 bp
Tandem dup.
Deletions
Inversions
Complex events
Translocations
Interchr. events
Foldbacks

**Supplementary Figure 7.** Experimental mutational signatures extracted for wild-type and 53 DNA repair deficient *C. elegans* strains. Each barplot depicts the average number of heterozygous (het.) mutations (mut-s) per generation (gen.) across the 119 mutation classes for the indicated genotype. Error bars denote 95% credible intervals. Headers indicate the average number of heterozygous mutations per generation, the range (minimum-maximum) of heterozygous mutations per generation, and the maximum generation with respective average observed mutation burden and observed range (in parentheses) across the samples used in the model. Strain details are provided in **Supplementary Table 1**. Genotypes are roughly ordered by DNA repair pathways (**Supplementary Table 1**). Mutation numbers of each mutation class per generation are provided in **Supplementary Data 1**.

# Supplementary Note 2. List of significant interaction effects between genotoxins and DNA repair deficiencies in *C. elegans*

We have estimated interaction effects for 196 combinations of *C. elegans* DNA repair deficiencies and genotoxin exposures (Methods). Of these, 88 combinations demonstrated significance in changing the rates of either base substitutions, indels, or structural variants (FDR 5% per category, see **Methods** for details). Fold-changes of mutation rates per individual mutation class between the signatures of respective genotoxins in a DNA repair deficient background versus in wild-type for these 88 significant combinations are listed below (**Supplementary Figure 8).**

Effects for agt.1.EMS

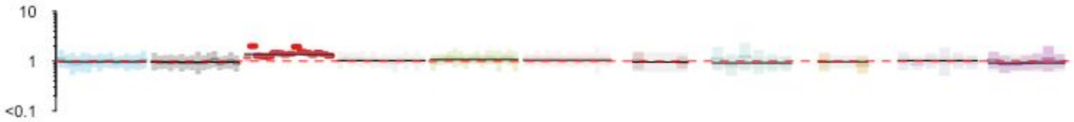


Effects for agt.1.MMS

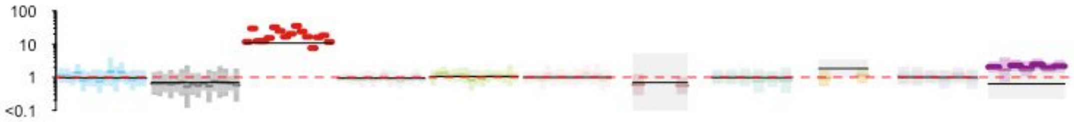


Effects for agt.1.Radiation

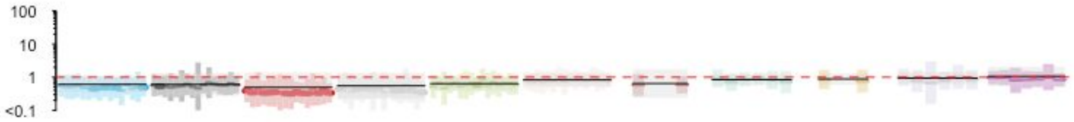


Effects for agt.2.MMS

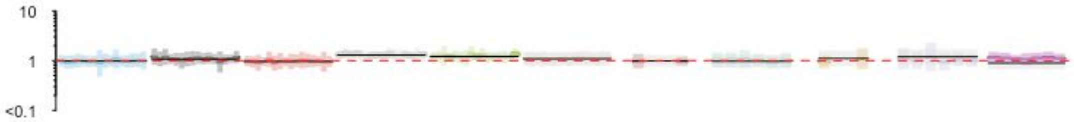


Effects for apn.1.MMS

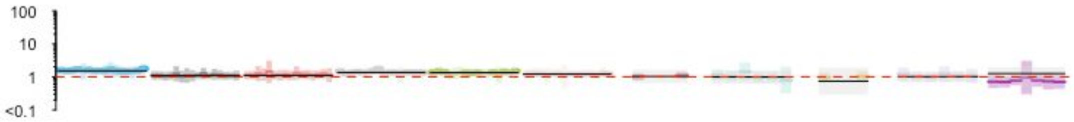


Effects for apn.1.Radiation

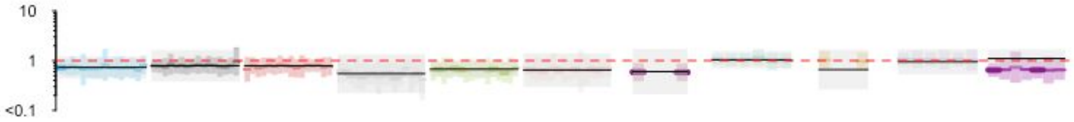

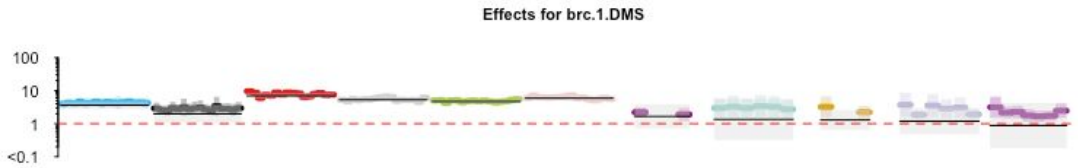
Effects for brc.1.DMS
100
10
1
<0.1

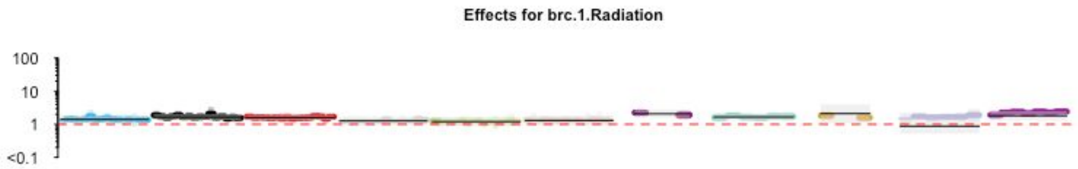
Effects for brc.1.Radiation
100
10
1
<0.1

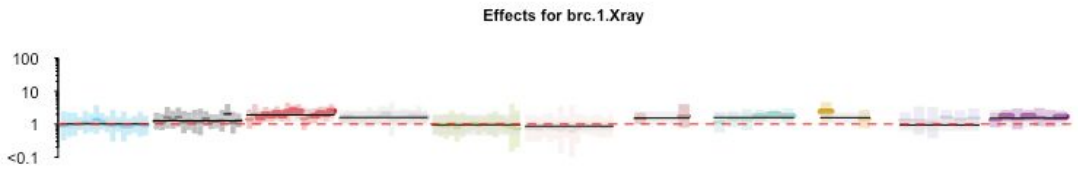
Effects for brc.1.Xray
100
10
1
<0.1

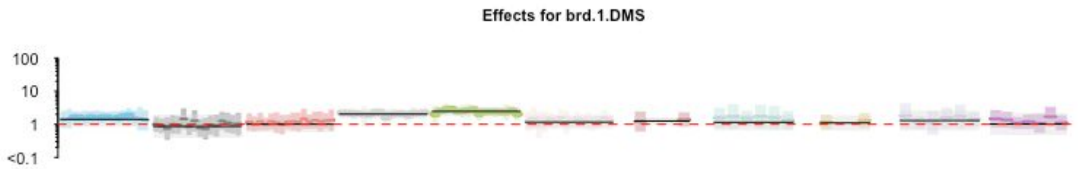
Effects for brd.1.DMS
100
10
1
<0.1

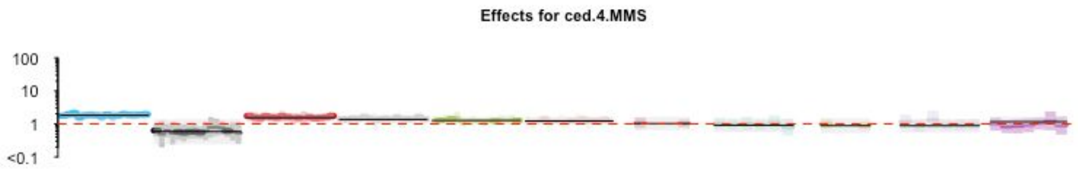
Effects for ced.4.MMS
100
10
1
<0.1

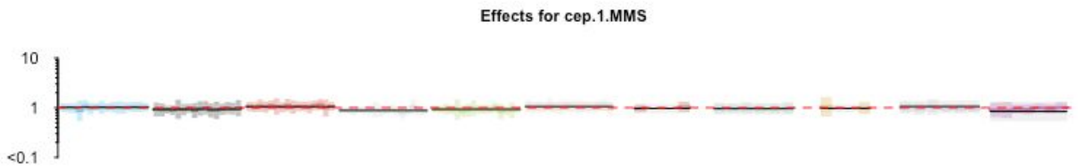
Effects for cep.1.MMS
10
1
<0.1

Effects for csb.1.Aflatoxin.B1

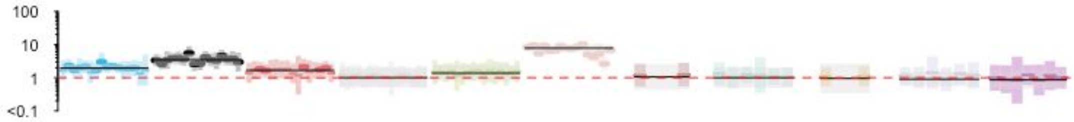


Effects for csb.1.DMS

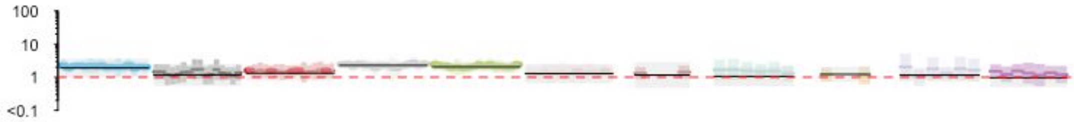


Effects for exo.3.Radiation

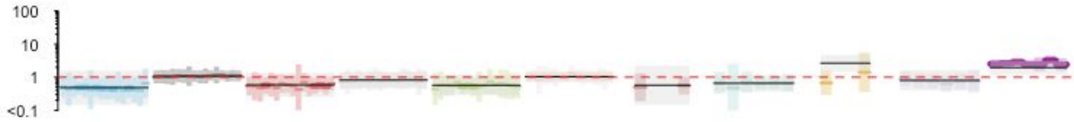


Effects for fan.1.Cisplatin

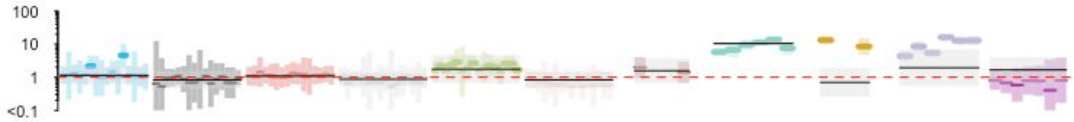


Effects for fan.1.DMS

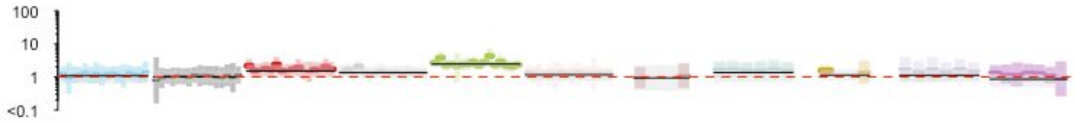


Effects for fan.1.EMS

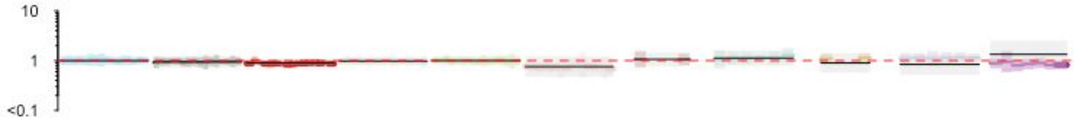

Effects for helq.1.DMS

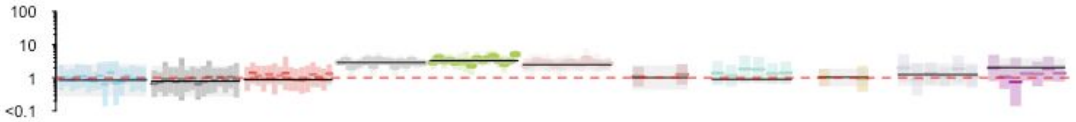


Effects for him.6.EMS

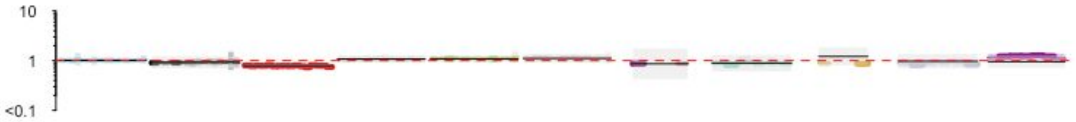


Effects for him.6.MMS

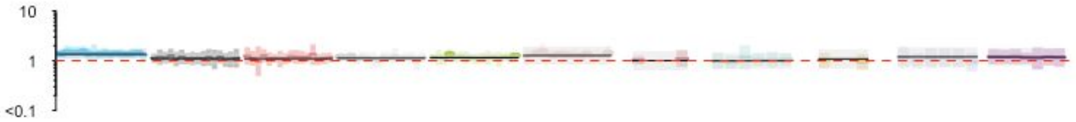


Effects for him.6.Radiation

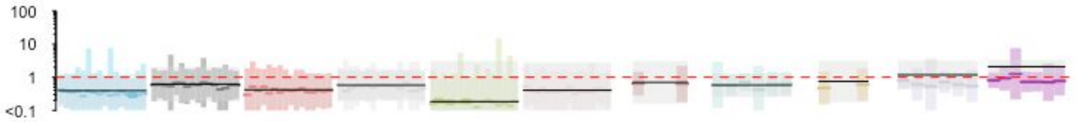


Effects for him.6.UV

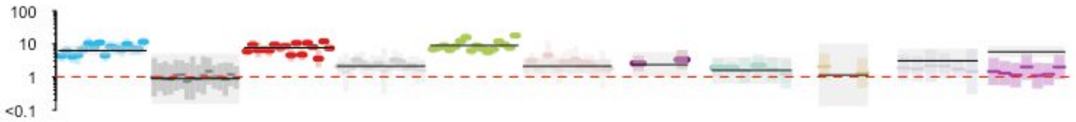


Effects for lem.3.Cisplatin

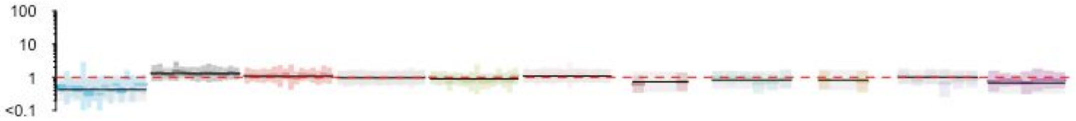

**Effects for lig.4.Cisplatin**

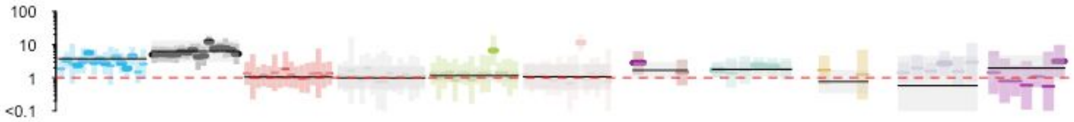


**Effects for mlh.1.DMS**

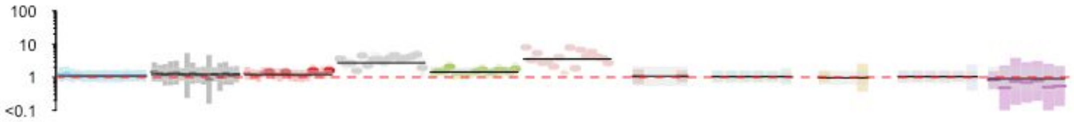


**Effects for mlh.1.EMS**

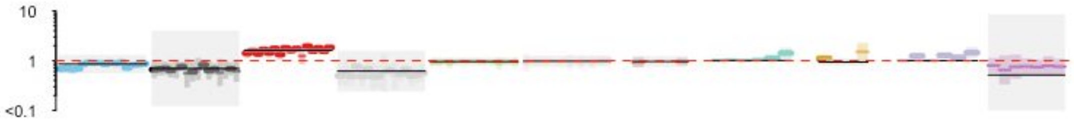


**Effects for mlh.1.MMS**

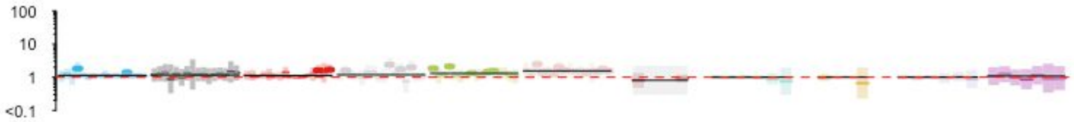


**Effects for mrt.2.MMS**

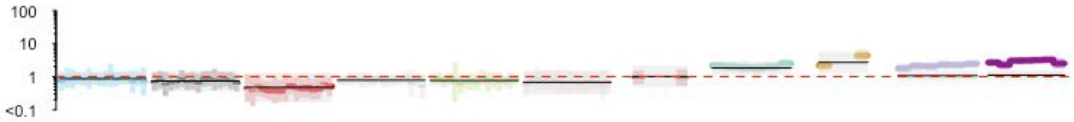


**Effects for mrt.2.Radiation**

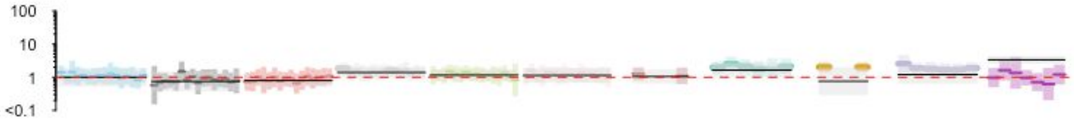

Effects for ndx.4.Radiation

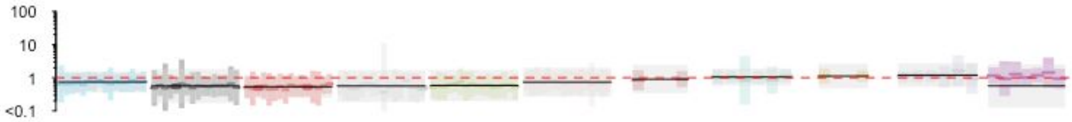


Effects for parp.1.Radiation

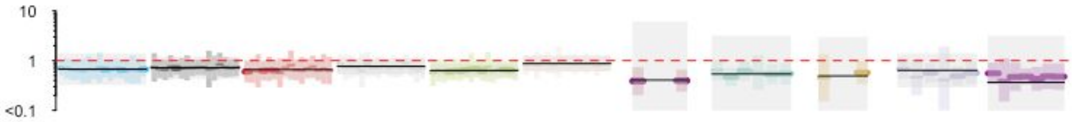


Effects for parp.2.DMS

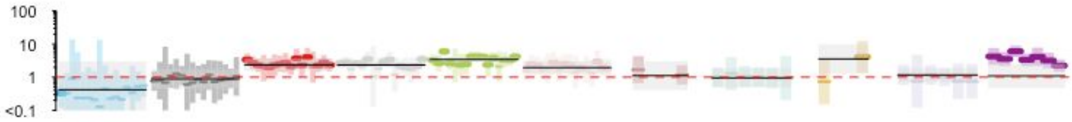


Effects for pole.4.DMS

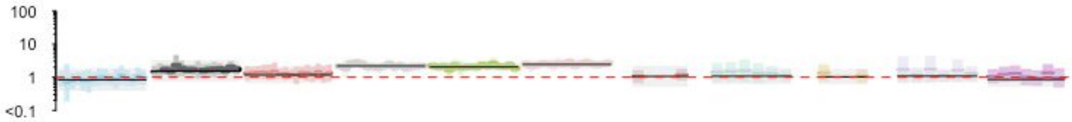


Effects for pole.4.MMS

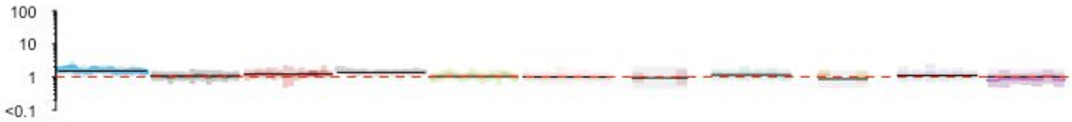


Effects for pole.4.Radiation

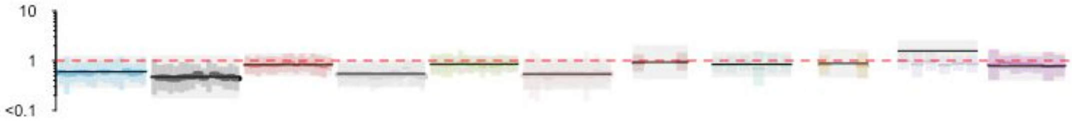

Effects for polh.1.Aflatoxin.B1

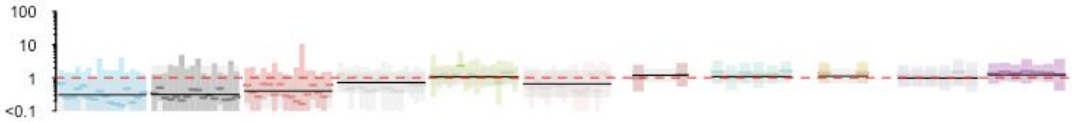


Effects for polh.1.AristolochicAcid

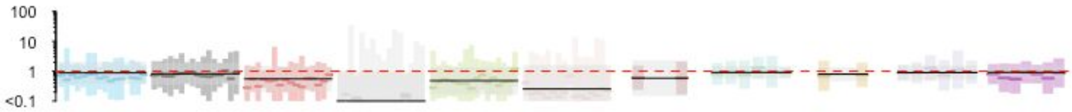


Effects for polh.1.EMS

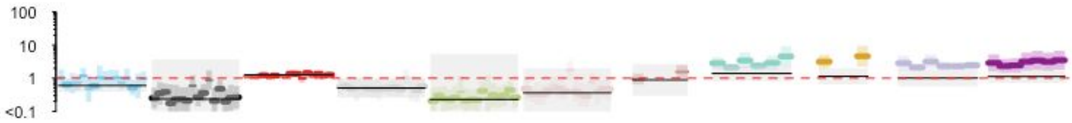


Effects for polh.1.Radiation

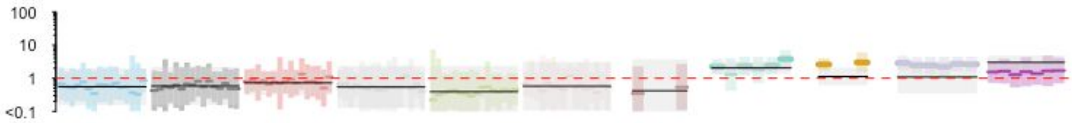


Effects for polk.1.DMS

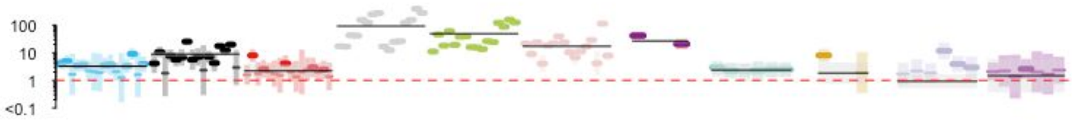


Effects for polk.1.EMS

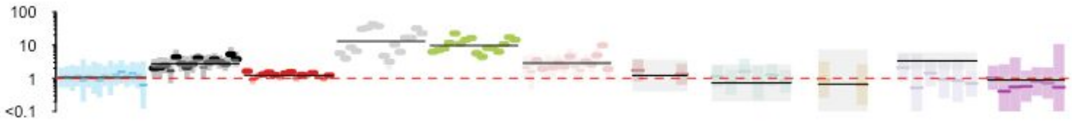

Effects for polk.1.MMS

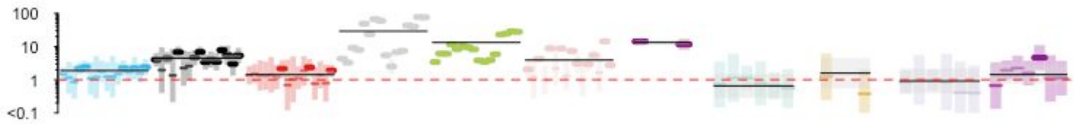


Effects for polq.1.AristolochicAcid

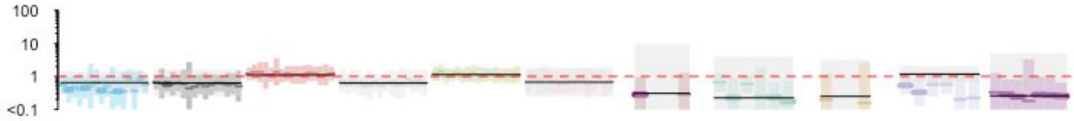


Effects for polq.1.Cisplatin

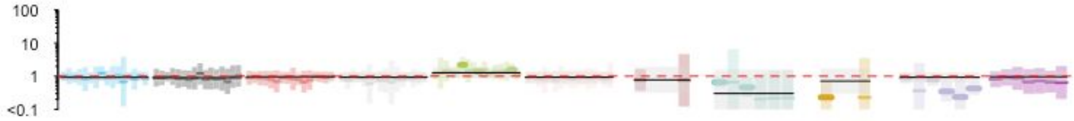


Effects for polq.1.DMS

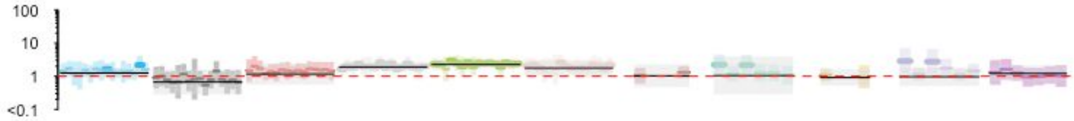


Effects for polq.1.EMS

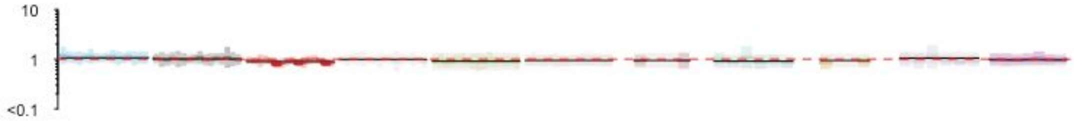


Effects for polq.1.UV

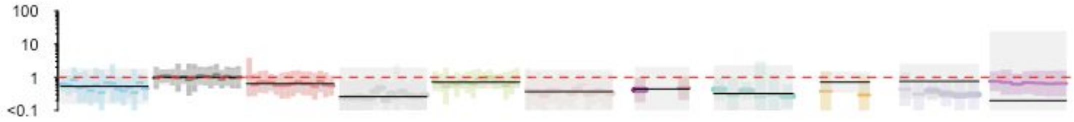

Effects for rad.54B..gt3308..Radiation

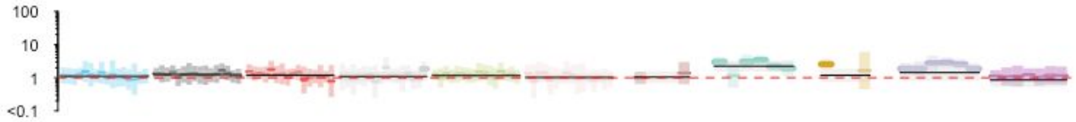


Effects for rcq.5.Radiation

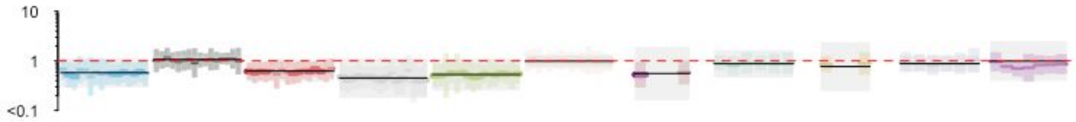


Effects for rev.1.AristolochicAcid

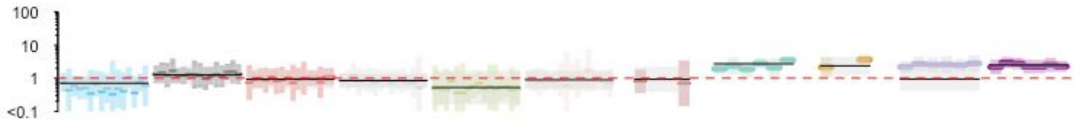


Effects for rev.1.DMS

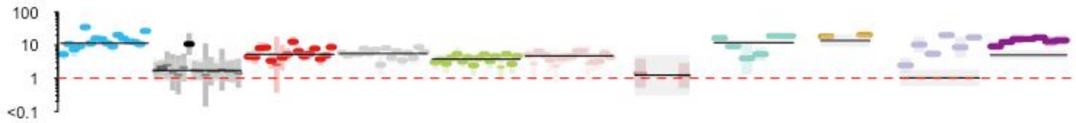


Effects for rev.3.Aflatoxin.B1

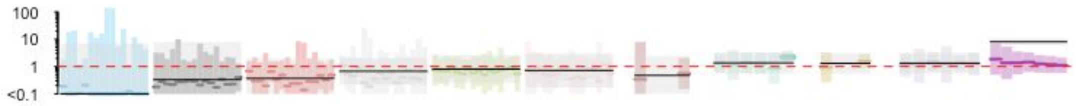


Effects for rev.3.AristolochicAcid

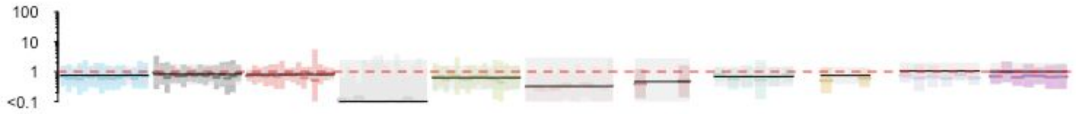

**Effects for rev.3.Cisplatin**

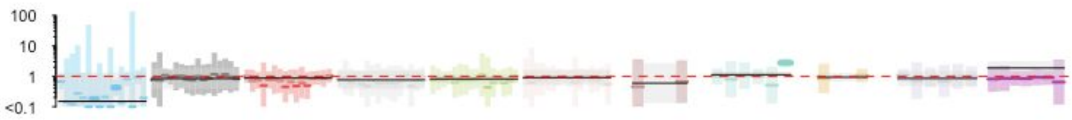


**Effects for rev.3.DMS**

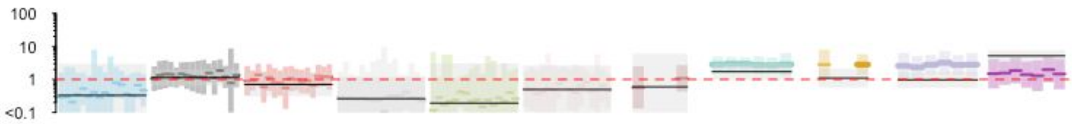


**Effects for rev.3.EMS**

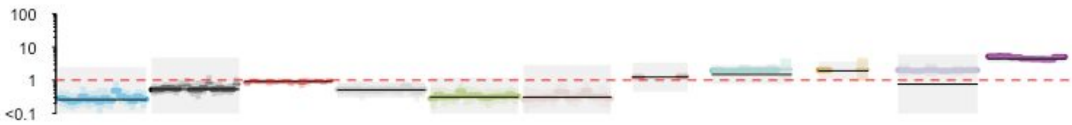


**Effects for rev.3.MMS**

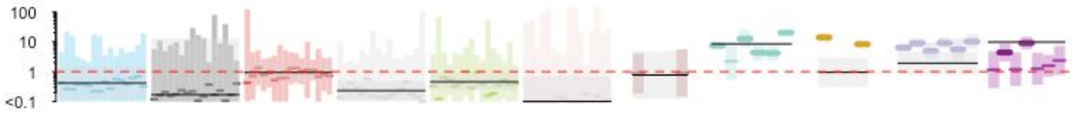


**Effects for rev.3.UV**

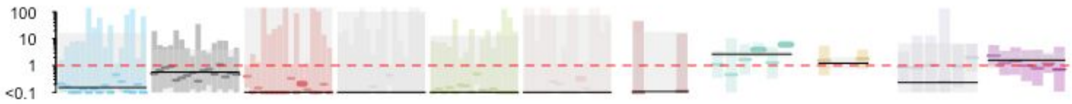


**Effects for rip.1.Radiation**

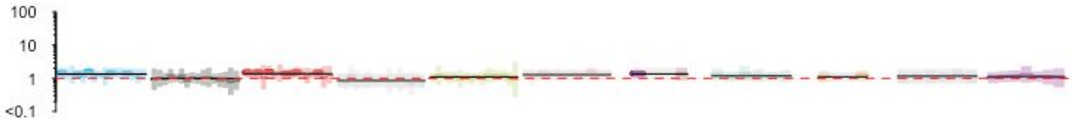

Effects for slx.1.DMS

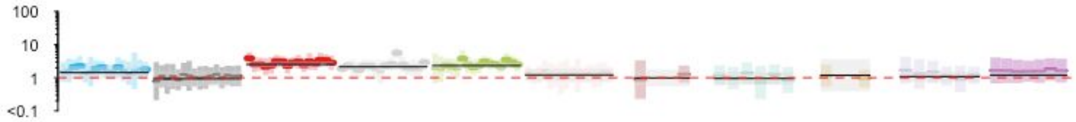


Effects for slx.1.MMS

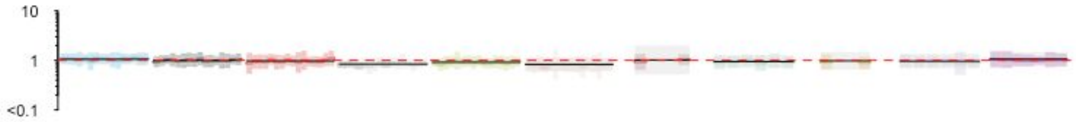


Effects for slx.1.Radiation

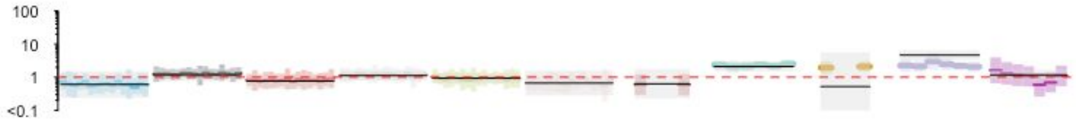


Effects for tdp.1.Radiation

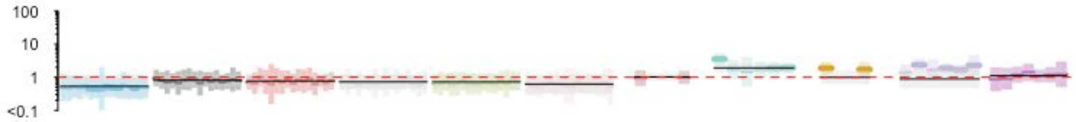


Effects for ung.1.DMS

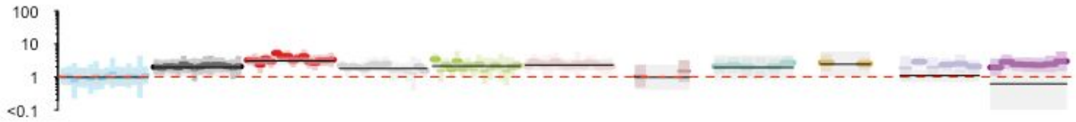


Effects for ung.1.EMS

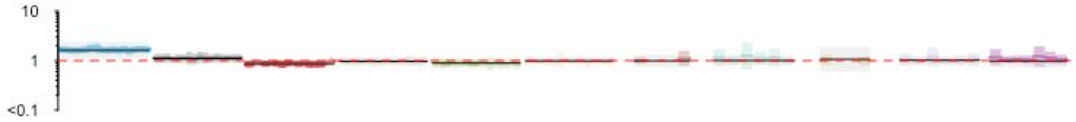

Effects for ung.1.Radiation

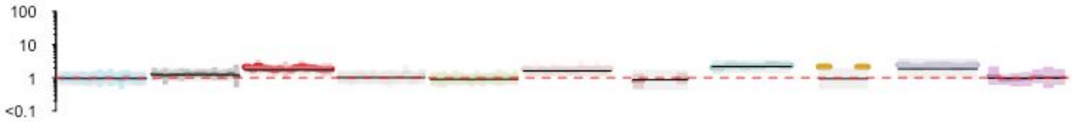


Effects for wrn.1.DMS

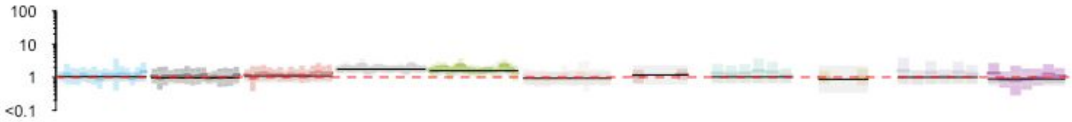


Effects for wrn.1.EMS

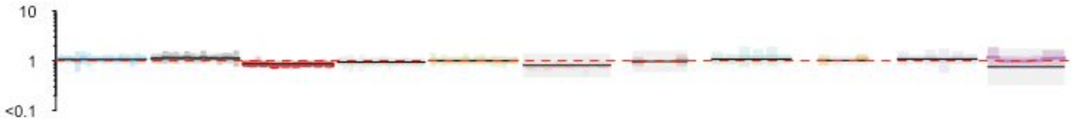


Effects for xpa.1.AristolochicAcid

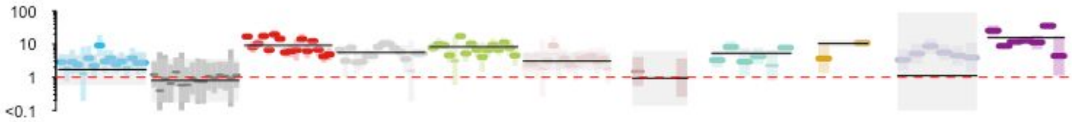


Effects for xpa.1.EMS

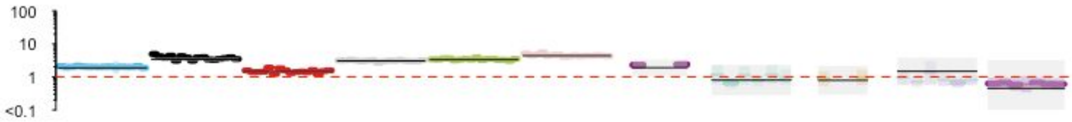


Effects for xpa.1.MMS

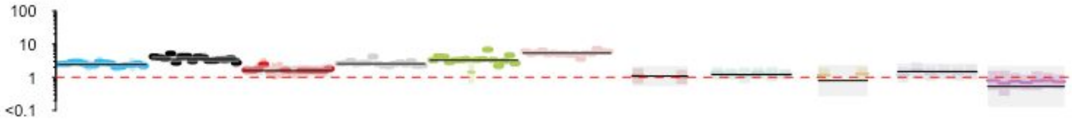

Effects for xpa.1.Radiation

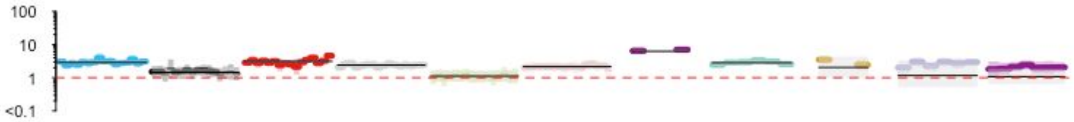


Effects for xpc.1.AristolochicAcid

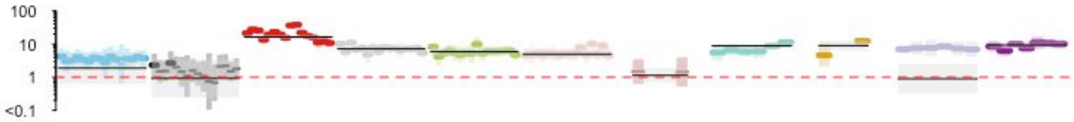


Effects for xpc.1.Cisplatin

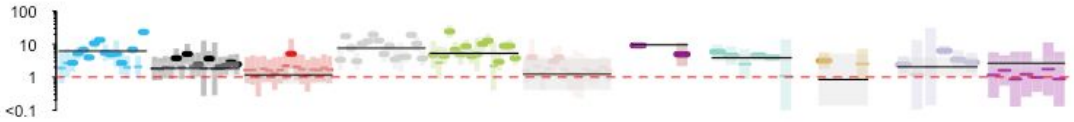


Effects for xpc.1.DMS

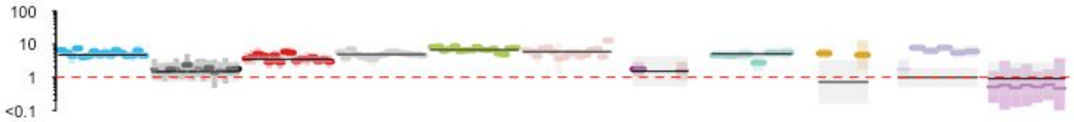


Effects for xpc.1.EMS

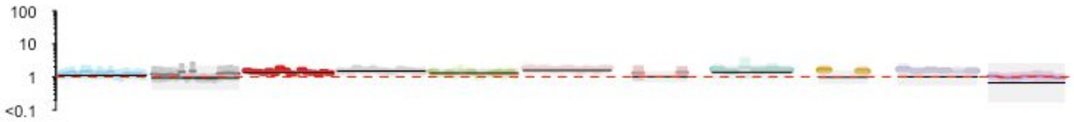


Effects for xpc.1.MMS

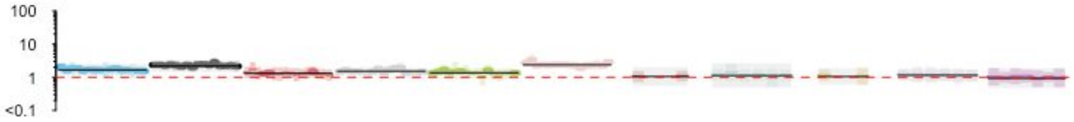

Effects for xpc.1.Radiation

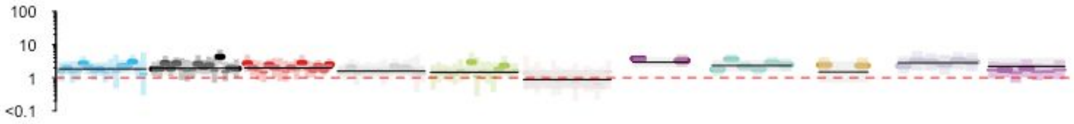


Effects for xpc.1.UV

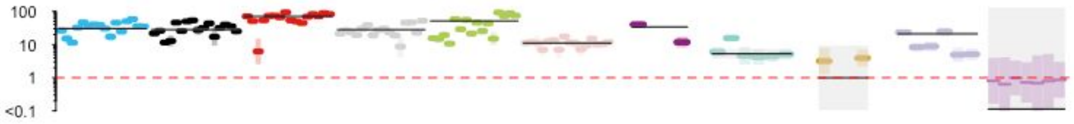


Effects for xpf.1.Aflatoxin.B1

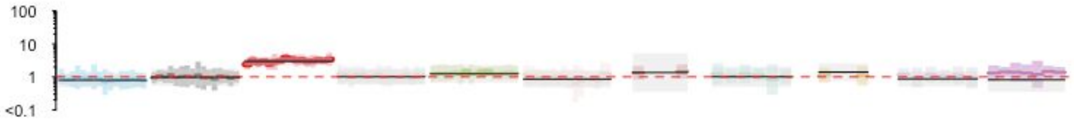


Effects for xpf.1.AristolochicAcid

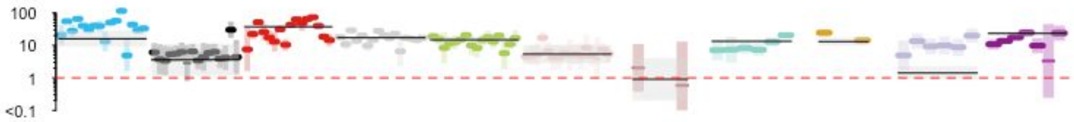


Effects for xpf.1.Cisplatin

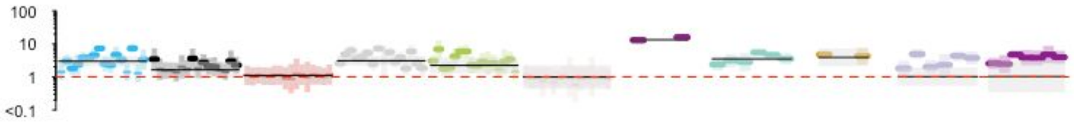


Effects for xpf.1.DMS

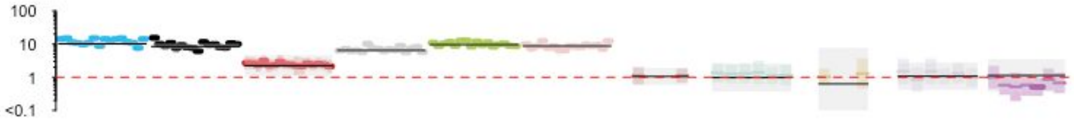

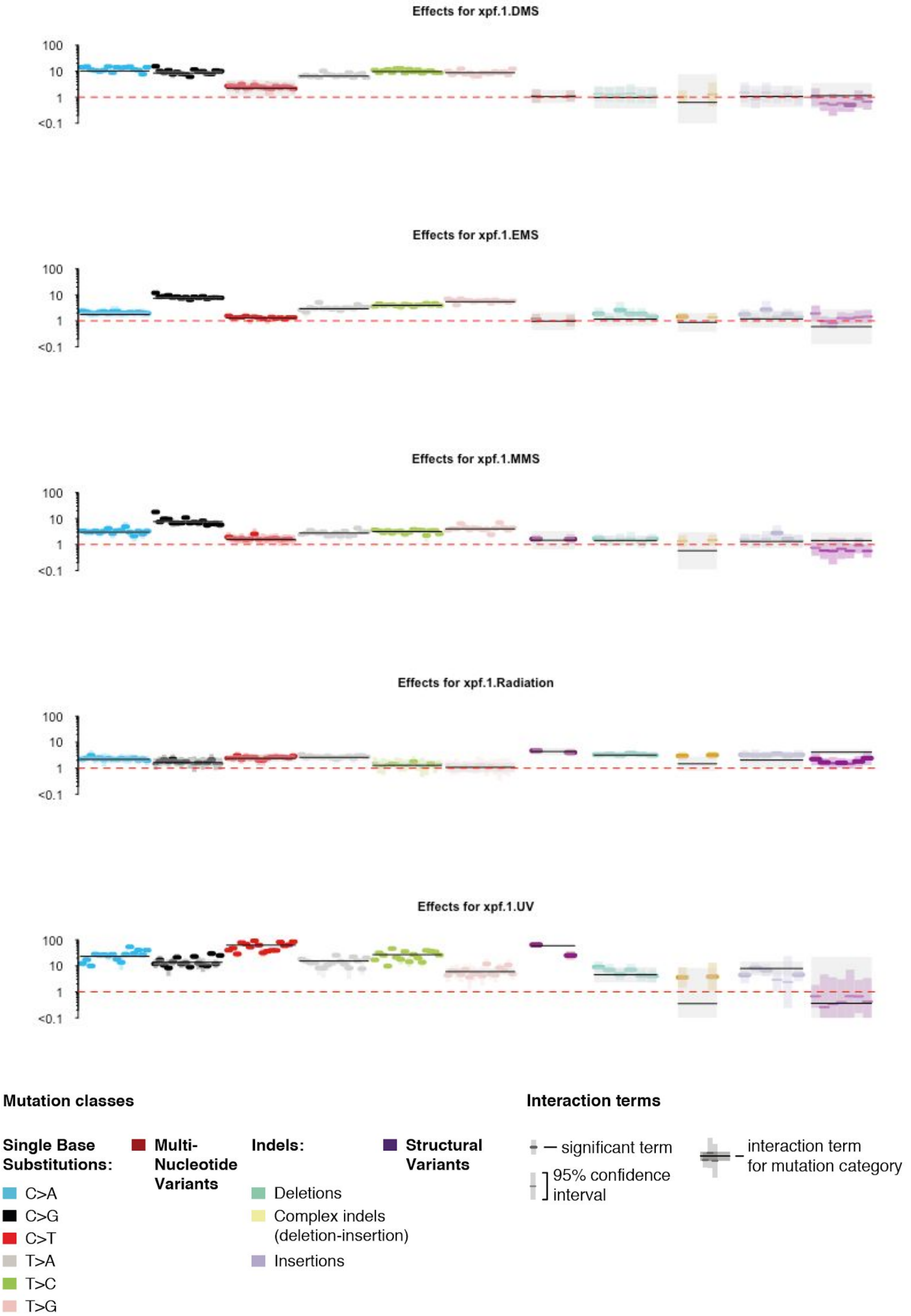
Effects for xpf.1.DMS
Effects for xpf.1.EMS
Effects for xpf.1.MMS
Effects for xpf.1.Radiation
Effects for xpf.1.UV
100
10
1
<0.1
Mutation classes
Single Base Substitutions:
C>A
C>G
C>T
T>A
T>C
T>G
Multi-Nucleotide Variants
Indels:
Deletions
Complex indels (deletion-insertion)
Insertions
Structural Variants
Interaction terms
significant term
95% confidence interval
interaction term for mutation category

**Supplementary Figure 8.** Estimated interaction effects for 88 combinations of *C. elegans* DNA repair deficiencies and genotoxin exposures which showed a significant effect either on the total mutation burden or the mutational profile. Each barplot reflects the fold-change in the number of mutations across the 119 mutation classes for the indicated genotype and mutagen per average dose. Point estimates for mutation classes with significant fold-changes (credible intervals do not intersect with 1, denoted by a red dotted line) are shown in darker colour. Strain details, mutagen doses and specifications are provided in **Supplementary Table 1** and **Supplementary Data 1**, numbers for interaction effects are shown in **Supplementary Data 4.**

# Supplementary Note 3. Frequency of biallelic DNA repair deficiency and selective pressure on DNA repair genes measured across TCGA

Tumours with somatic heterozygous mutations in genes required for DNA repair and DNA damage signaling are very common, reaching nearly 100% for the DNA damage sensing (DS) pathway in ovarian cancers and uterine carcinosarcomas due to very high rates of TP53 mutations [4]. In contrast, we found that somatic biallelic DNA repair gene deficiency is generally rare (**Supplementary Figure 1b, Supplementary Data 3**), suggesting that single DNA repair defects in cancers are rarely associated with a change in mutation rate or spectrum. Exceptions were damage sensing defects (mostly well-studied *TP53* mutations), biallelic MMR defects, HR deficiency, and  monoallelic mutations of the polymerase epsilon (POLE) exonuclease domain (**Supplementary Figure 1c,d**).

In order to infer interactions between suspected genotoxin exposure and DNA repair deficiency in cancer genomes, we adapted the Negative Binomial model used for the *C. elegans* experimental system to cancer genomes (see main **Methods**). Applying this approach to a range of cancers with suspected genotoxic exposures and stratifying based on DNA repair status, we were able to detect cases of DNA repair and damage interaction leading to increased mutagenesis and/or altered signature. We found that such interactions were rare and typically only revealed moderate effects. Nevertheless, a number of noteworthy examples exist, illustrating the action of different DNA repair pathways, including DR, MMR, and TLS, upon exposure to distinct types of DNA damage conferred by APOBEC enzymes, polymerase proofreading deficiency, UV exposure and temozolomide treatment (**Figure 2d**).

Given that the association between DNA repair pathway defects and mutation phenotypes in cancer genomes appears weaker than expected, we took an evolutionary approach to investigate if changes in DNA repair status nevertheless affect oncogenesis. Using the rate ratio of non-silent (N) to silent (S) mutations dN/dS in a given gene as an established method for quantifying positive selection in cancer (**Supplementary Methods**) [5], we found that overall 7/248 genes involved in DNA repair displayed significant signs of positive selection for missense mutations, and 17/248 DNA repair genes were enriched for truncating mutations (FDR<10%; **Supplementary Figure 3h**, **Supplementary Data 5**). Among these the majority were known cancer genes such as TP53, IDH1, PTEN, ATM, ATRX, SMARCA4, BRCA1/2, MLH1, but we also detected new genes with mild signs of positive selection, namely the DNA damage sensing protein kinase PRKDC (dN/dS = 2 for nonsense variants; q-value = 0.01), the pre-MRNA processing factor PRPF19, involved in TCR, (dN/dS = 4.6 for nonsense variants; q-value = 0.005) and the homologous recombination repair gene SWI5 (dN/dS = 3.2 for missense and 5.3 for nonsense variants; q-value = 0.05 and 0.07, respectively).

## Supplementary Note 4. Tissue-specific effects of MMR deficiency

Among other susceptible interactions between MMR deficiency and DNA damaging processes, previous studies also suggested a role for MMR in the repair of deaminated 5-methylcytosines [6]. Using the same approach (**Supplementary Methods**), we assessed the variability of MMR deficiency signature in cancer types originating from different tissues and different MMR gene mutations.

We first looked at the effects of each of the MMR genes (MLH1, MLH3, PMS2, MSH2, MSH3, and MSH6) being mutated (using 6 covariates, one for each gene), and did not find any significant effect. Next, we looked at the tissue effects (comparing 7 tissues - breast, cervix, head and neck, lung, stomach, uterus, liver - to colorectal), where we observed changes in the rates of mononucleotide variants, insertions and deletions in homopolymeric stretches, and variation in C>T mutations in a CpG context across different tissues (**Supplementary Figure 3c,d**). Compared to MMR proficient tumours, MMR deficient tumours showed on average a 5-fold higher rate of single base deletions per year following the same trend across different tissues. Moreover, the single base insertion rate was increased on average 4-fold, and the rate of C>T at CpG sites was 3-fold higher in the samples with MMR defects compared to the tissue baseline. These data square well with the observation that MMR deficiency is associated with a number of additional mutational signatures of unknown aetiology [6], suggesting that these may reflect distinct mutagenic processes which are exacerbated by MMR deficiency.

# Supplementary Tables

## Supplementary Table 1. Strains used in the study.

| Gene | Allele | Genomic location (Wormbase v.WS268, # this study) | Mutation | Mutation type | Human ortholog | DNA damage response pathway | Additional information |
|---|---|---|---|---|---|---|---|
| *agt-1* | gk563117 | IV:13369361..13369361 | C>T | point mutation | *MGMT* | DR | |
| *agt-2* | tm6462 | II:5372033..5372401 | 369 bp | deletion | (alkylguanine DNA alkyltransferase) | DR? | |
| *apn-1* | cxTi10435 | II:8048817..8048817 (#) | insertion | Mos transposon insertion | (apurinic/apyrimidinic endonuclease) | BER | |
| *brc-1** | tm1145* | III:3861888..3862558 | 669 bp | deletion | *BRCA1* | DSBR - HR | * contains additional deletion in brd-1 |
| *brd-1* | gk297 | III:6888360..6888743 | 384 bp | deletion | *BARD1* | DSBR - HR | |
| | | | 1800 bp | deletion | | | *brd-1 allele in brc-1 mutants* |
| *bub-3* | gt2000 | II:13727714..13727714 (#) | C>T | point mutation | *BUB3* | SAC | |
| | ok3437 | II:13727485..13727819 | 335 bp | deletion | | | |
| *ced-3* | n717 | IV:13200009..13200009 | C>T | point mutation | *CASP3* | DS | |
| *ced-4* | n1162 | III:4852562..4852562 | C>T | point mutation | *ARAF1* | DS | |
| *cep-1* | lg12501 | I:8327197..8328410 (#) | 1213 bp | deletion | *p53* | DS | |
| *cku-80* | ok861 | III:4420642..4422287 | 1646 bp + 315 bp | deletion + insertion | *KU80* | DSBR - NHEJ | |
| *csb-1* | ok2335 | X:15862559..15864178 | 1620 bp | deletion | *ERCC6* | NER | |
| *dna-2* | gk659160 | II:11779180..11779180 | C>T | point mutation | *DNA2* | Helicase | |
| *dog-1* | gk10 | I:15020324..15022353 | 2030 bp | deletion | *FANCJ* | CLR | |
| *exo-1* | tm1842 | III:13423988..13424546 | 559 bp | deletion | *EXO1* | BER | |
| *exo-3* | ok3539 | I:11908374..11908970 (#) | 596 bp | deletion | *APEX1* | BER | |
| *fan-1* | tm423 | IV:6550140..6550550 | 411 bp | deletion | *FAN1* | CLR | |
| *fcd-2* | tm1298 | IV:15032211..15032447 | 237 bp | deletion | *FANCD2* | CLR | |
| *fnci-1* | tm3081 | I:6750926..6751802 | 879 bp | deletion | *FANCI* | CLR | |
| *fncm-1* | tm3148 | I:5190288..5190797 | 510 bp | deletion | *FANCM* | CLR | |
| *helq-1* | tm2134 | III:584726..585071 | 346 bp deletion | deletion | *HELQ* | CLR | |
| *him-6* | ok412 | IV:12491122..12492807 | 1686 bp | deletion (del within duplication (#)) | *BLM* | Helicase / RecQ family | |
| *lem-3* | mn155 | I:9580597..9580597 | G>A | point mutation | *ANKLE1* | DS | |
| *lig-4* | ok716 | III:7522721..7524262 | 1542 bp | deletion | *LIG4* | DSBR - NHEJ | |
| *mlh-1* | ok1917 | III:13509535..13510634 | 1100 bp | deletion | *MLH1* | MMR | |
| *mrt-2* | e2663 | III:11747433..11747433 | G>A | point mutation | *RAD1* | TR | |
| *mus-81* | tm1937 | I:4264898..4266089 | 1192 bp | deletion | *MUS81* | DSBR + CLR | |

| | | | | | | | |
|---|---|---|---|---|---|---|---|
| *ndx-4* | ok1003 | I:13792626..13793410 | 785 bp + AAAA | deletion + insertion | *NUDT2* | BER | |
| *parp-1* | ok988 | I:2923535..2924541 | 1007 bp + 40 bp | deletion + insertion | *PARP1* | BER | |
| *parp-2* | ok344 | II:9593413..9594988 | 1576 bp | deletion (del within duplication (#)) | *PARP2* | BER | |
| *pole-4* | tm4613 | II:14968615..14969321 | 707 bp + 20 bp | deletion + insertion | *POLE4* | Replicative polymerase | |
| *polh-1* | ok3317 | III:1949310..1949865 (#) | 556 bp | deletion | *POLH* | TLS | |
| *polk-1* | lf29 | III:8627544..8627544 | G>A | point mutation | *POLK* | TLS | |
| *polq-1* | tm2026 | III:5796555..5797466 | 912 bp | deletion | *POLQ, some POLN homology* | DSBR - MMEJ | |
| *rad-51* | gt3306 | IV:10282426..10282426 (#) | C>T | point mutation | *RAD51* | DSBR - HR | |
| *rad-54B* | gt3308 | IV:16956998..16956998 (#) | G>A | point mutation | *RAD54B* | DSBR - HR | |
| | gt3312 | IV:16953960..16953960 (#) | C>T | point mutation | | | |
| *rcq-5* | ok660 | III:4055071..4056369 | 1299 bp | deletion | *REQ5* | Helicase / ReQ family | |
| *rev-1* | gk924750 | II:7903081..7903081 | C>T | point mutation (P600L) | *REV1* | TLS | |
| *rev-3* | gk919715 | III:5778506..5778506 | G>A | point mutation, Q to ochre stop | *REV3L* | TLS | |
| *rfs-1* | ok1372 | III:8226553..8227823 (#) | 1270 bp | deletion (del within duplication (#)) | *RAD51D (RAD51 paralog)* | DSBR - HR | |
| *rip-1* | tm2948 | III:10257746..10258413 | 668 bp | deletion | (RFS-1 interacting protein) | DSBR - HR | |
| *san-1* | ok1580 | I:6407947..6408938 | 992 bp del | deletion | *BUB1B* | SAC | |
| *slx-1* | tm2644 | I:5185146..5185352 | 207 bp | deletion | *SLX1* | DSBR, CLR | |
| *smc-6* | ok3294 | II:11579498..11580328 (#) | 800 bp deletion | deletion | *SMC6* | DSBR | |
| *smg-1* | gk761853 | I:6912211..6912211 | C>T | point mutation | *SMG1* | DS | |
| *tdpo-1* | gk889420 | IV:1960798..1960798 | T>A | point mutation, Y to ochre stop | *TDP1* | BER | |
| *ung-1* | tm2862 | III:11973279..11973571 | 293 bp | deletion | *UNG* | BER | |
| *wrn-1* | gk99 | II:6554817..6555012 | 196 bp | deletion | *WRN* | Helicase / ReQ family | |
| *xpa-1* | ok698 | I:7160088..7161000 | 913 bp | deletion | *XPA* | NER | |
| *xpc-1* | tm3886 | IV:1994043..1994516 | 474 bp + 24 bp | deletion + insertion | *XPC* | NER | |
| *xpf-1* | tm2842 | II:11696824..11697166 | 343 bp | deletion | *XPF* | NER | |

**Supplementary Table 1.** Strains used in the study. List of DNA repair mutants used including gene names, alleles, genomic location and type of variant, human orthologs, function and additional information.

Abbreviations: BER - base excision repair, CLR - crosslink repair, DR - direct damage reversal, DS - damage sensing/checkpoint, DSBR - double-strand break repair, HR - homologous recombination, NHEJ - non-homologous end-joining, MMEJ - microhomology-mediated end-joining, MMR - mismatch repair, NER - nucleotide excision repair, TLS - translesion synthesis, TR - telomere replication, SAC - spindle assembly checkpoint.

# Supplementary Table 2. TCGA data sources.

| TCGA project | Intended use | Link to the data/study |
|---|---|---|
| Glioblastoma multiformae - GBM | Analysing the interaction between temozolomide signature and *MGMT* status | Kim et al. 2015 [7] |
| ACC, BLCA, BRCA, CESC, COAD, ESCA, GBM, NHSC, KICH, KIRC, KIRP, LAML, LGG, LIHC, LUAD, LUSC, MESO, OV, PAAD, PCPG, PRAD, READ, SARC, SKCM, STAD, TGCT, THCA, THYM, UCEC, UCS | Checking the presence and frequency of high-impact mutations in DNA repair genes<br>Testing interactions between REV1/UNG defects and APOBEC signature appearance, NER defects and UV exposure, NER defects and tobacco exposure, POLE exonuclease domain defects and MMR deficiency, as well as MMR deficiency defects across tissues. | Raw data available from https://portal.gdc.cancer.gov/<br>Filtered variant calls were obtained from [5]<br>Variant interpretation was cross-checked with the available analyses from the original studies: SKCM [8], LUSC [9], LUAD [10], UCEC [11], BLCA [12] |
| Same as above | Assessing the status of DNA repair genes via checking their copy number, expression, and methylation | https://portal.gdc.cancer.gov/repository (transcriptome profile, copy number variation, dna methylation) |

**Supplementary Table 2**. TCGA data sources, with the links and the description of their intended use in the manuscript. Full names of TCGA cancer projects can be found at the GDC website

(https://gdc.cancer.gov/resources-tcga-users/tcga-code-tables/tcga-study-abbreviations) or in **Supplementary Data 3**.

# Supplementary Methods

## 1. Comparison between *C. elegans*, iPS cell and cancer derived signatures of genotoxins

We wished to compare the experimental mutational signatures of genotoxin exposure between *C. elegans* and human iPS cells [13], and explore their similarity to mutational signatures computationally extracted from tumour mutation spectra[14]. With a mean cosine similarity of 0.63 (range 0.20-0.84), the *C. elegans* experimental signatures (adjusted to the human trinucleotide frequencies (**Methods**)) generally display a good level of similarity with their human experimental counterparts, and at times even better concordance with computationally derived cancer signatures with the same suspected origin (**Supplementary Figure 2b**).

In particular, the *C. elegans* signature of the DNA ethylating agent EMS was strikingly similar (0.90) to the cancer-derived computational signature SBS11 associated with temozolomide treatment, yet different from the temozolomide signature derived from iPS cell lines (**Supplementary Figure 2c**). This may be due to iPSC-specific metabolic activation of temozolomide, or altered use of DNA repair pathways in these cells. The reported mutation spectrum observed in *Salmonella typhimurium* upon EMS exposure is nearly identical to the one observed in *C. elegans* [15,16]. Similarly, the iPSC-derived experimental signature of the methylating agent DMS demonstrated a preference for C>T changes, a base substitution almost absent in the *C. elegans* DMS signature (**Supplementary Figure 2c**).

Cisplatin treatment yielded a signature which only resembled the C>A part of the spectrum observed iPSCs and cancers [1,14,17], similar to that reported in chicken fibroblasts which also consisted primarily of C>A changes [18]. *C. elegans* exposure to UV-B resulted in a mutation spectrum dominated by C>T, similar to that in cell lines and human cancer samples, albeit with an additional fraction of T>C mutations (**Supplementary Figure 2c**). This discrepancy might be due to the difference in UV sources used. Our experiments were performed using a UV-B source, whereas[19] used a mixture of 90% UV-A and 10% UV-B to mimic the actual UV spectrum contained in the sunlight.

Importantly, *C. elegans* spectra resulting from the exposure to ionizing radiation, generally associated with the equal mutagenesis of all bases, share significant similarity with the mutation spectrum observed in secondary tumours from tissues previously exposed to radiotherapy [3] (**Supplementary Figure 2c**).

Signatures of aflatoxin B1 and aristolochic acid exposure were similar in all systems.
This similarity reflects the fact that the majority of DNA repair pathways are highly conserved among eukaryotes, but also that DNA repair capacity and genotoxin metabolism may differ moderately between nematodes, human cell lines, and cancer cells. Indeed, some of the experimental signatures derived from human cells, such as signatures of aflatoxin and

temozolomide (**Supplementary Figure 2c**), were less similar to cancer signatures than *C. elegans* signatures.

# 2. Microhomology analysis for deletion breakpoints in *C. elegans*

Microhomology search was conducted for all medium-size deletions observed in the *C. elegans* dataset. We looked for precise homology at the breakpoints using ce11 *C. elegans* genome build. The expected distribution of microhomologies across deletions was calculated based on the probability of encountering the same k-mer around the breakpoints as described in [20]. We then fitted an additive Poisson GLM to describe the number of deletions with MH of a certain size as a combination of random contribution, 1-bp MH with their associated distribution of higher order MH occurring by chance ( $P(MH_{obs} = 2|MH_{real} = 1) = \frac{P(MH_{obs}=2)}{P(MH_{real}=1)}$ for *j = 2, 3, ...* ), similar terms for 2-bp and 3-bp MH, and for MH longer than 3 as the numbers of deletions with longer MH were too low to permit confident estimation. For the deletions across non-aristolochic acid (AA) exposed samples, the model predicted that 52% (SD = 3.5%) of all deletions occurred in an MH-independent manner, 24% (SD = 3.4%) were assigned to 1-bp MH, 10% (SD = 2.5%) to 2-bp MH, 4% (SD = 1.7%) to 3-bp MH, and 10% (SD = 1.4%) were predicted as dependent on longer MH. For deletions in AA-exposed samples, 40% (SD = 13%) of all deletions were predicted as non-random, with a higher bias towards 1-bp MH: 35% (SD=13%) of deletions were predicted as dependent on 1-bp MH, with no deletions assigned to higher-order MH (**Supplementary Figure 5b**). According to the overall distribution of indels across all samples, AA-exposed samples, and *polq-1* deficient mutants (**Supplementary Figure 5c**), AA-exposed samples displayed more indels in the range between 50 and 300 bp than an average sample, whereas *polq-1* mutants had almost no indels longer than 10 bp.

# 3. DNA repair analysis across TCGA

## 3.1 Motivation

DNA repair deficiencies have a crucial role in cancer development. However, only a few of them have been assigned an associated mutational signature. We established a catalogue of potential DNA repair pathway deficiencies in human cancers by annotating heterozygous and homozygous somatic missense and loss-of-function mutations, as well as epigenetic gene silencing in 81 core genes of the 9 consensus DNA repair pathways [4,21] across 9,946 patients and 30 cancer types available from TCGA (**Supplementary Data 3, Supplementary Figure 1a,b**). Tumours with somatic heterozygous mutations in genes required for DNA repair and DNA damage signaling are very common, reaching nearly 100% for the DNA damage sensing (DS) pathway in ovarian cancers and uterine carcinosarcomas due to very high rates of TP53 mutations [4]. Somatic biallelic DNA repair gene deficiency, in contrast, was generally rare (**Supplementary Figure 1b**), suggesting that single DNA repair defects in cancers are rarely associated with a change in mutation rate or spectrum. Exceptions were damage sensing defects (mostly well-studied *TP53* mutations), biallelic MMR defects, HR deficiency, and  monoallelic mutations of the polymerase epsilon (POLE) exonuclease domain (**Supplementary Figure 1c,d**).

## 3.2. Data description

Unless not stated otherwise, data was obtained from GDC (https://cancergenome.nih.gov) and filtered according to [5]. Somatic and germline mutations were acquired using cgpCaVEMan (http://cancerit.github.io/CaVEMan/) and cgpPindel (https://github.com/cancerit/cgpPindel) variant callers. Copy number profiles, purities and ploidies of TCGA samples were estimated using ASCAT [22].

## 3.3. Classification of mutations across pathways and labeling of samples: monoallelic and biallelic deficiency

DNA repair genes across 9 pathways were selected as core or accessory components based on the combined classifications from [4,21] excluding genes indicated as consistently undercovered in exome sequencing [23] (**Supplementary Data 3**). We investigated point mutations, indels and copy number states for each gene. Samples were classified as having monoallelic deficiency of a given DNA repair pathway if at least one of the core components of this pathway carried a high-impact mutation of allele frequency higher than 0.4 (damaging mutation is defined as being a missense, nonsense, frameshift, essential splice site, 'stop gained', 'stop lost' or 'start lost' mutation), or is located in a region with major copy number 1, or is methylated with average methylation score between 0.2 and 0.75 (see below).

Methylation analysis was based on the respective methodology from [4]. For each cancer type where methylation and expression data were available, we selected the CpG sites relevant to the gene of interest by proximity (using the annotation from "IlluminaHumanMethylation450kanno.ilmn12.hg19" R package, v. 0.6.0, https://bioconductor.org/packages/IlluminaHumanMethylation450kanno.ilmn12.hg19/ ) and by correlation with the expression of this gene (with a cutoff of -0.5). Then we assessed the median methylation of these CpG sites across the samples within the cancer type. Assuming that the gene of interest with median beta value lower than 0.1 can be considered unmethylated, we calculated z-scores of expression values within the group of samples with unmethylated gene of interest. Then we iteratively adjusted the group of samples with "methylated" gene of interest by considering first all samples with median beta value of at least 0.2 in the gene of interest, and then removing samples with lowest beta values until the "methylated" group contained less than 3 samples, its mean expression z-score reached 5% quantile of the distribution of expression z-scores in unmethylated group, or until the difference in expression values between the groups fell below 2-fold. Then, if the resulting group of samples with presumably methylated gene of interest is meaningful (larger than 2 samples, with at least one sample with a median beta value >0.5, with average expression value at least 50% lower than that in unmethylated group, and with mean expression z-score below 5% quantile of normal distribution), we additionally confirm the difference using one-tailed Student t-test on z-scores between "methylated" and unmethylated groups. If it results in a p-value lower than 0.05, the samples from "methylated" group are further labeled as such. Using this approach, we identified significant groups of methylated samples for MLH1, MLH3, MGMT, REV3L and TP53.

Cancer samples were further labelled as carrying biallelic deactivation of a certain pathway if a sample had a damaging mutation of variant allele frequency >0.8 in any core component of

this pathway, or if this core gene was subject to deep deletion (belonged to a region with major copy number 0), or if it was methylated with a median beta value >0.8.

Monoallelic deficiency in proofreading ability of polymerase epsilon was assessed by screening for damaging mutations in the proofreading domain of POLE (residues 268 to 471) with VAF > 0.2.

## 3.4. Analysis of mutational burden and change in the mutational profile

### 3.4.1 Mutation burden

The change in mutation burden between samples with functional and defective DNA repair pathways was performed per cancer type. In total, we analyzed 8797 samples which had more than 50 but less than 20000 mutation per exome. We further looked at the mutation rates per year per Megabase in the samples without any defects in a certain pathway, those with only monoallelic defects, and those with biallelic. Each group was only considered if it contained more than 4 samples (samples with mutations in POLE proofreading domain mutations were removed from all other analyses but POLE and POLE+MMR). The increase in average mutation rate between the wild-type and heterozygous (and, when available, wild-type and homozygous) groups was tested using one-tailed Wilcoxon test. P-values were further adjusted for multiple testing correction using Benjamini-Hochberg procedure (**Supplementary Figure 1c**).

In addition to the Wilcoxon test, for the cases with significant change in the mutation rates we also tested how likely it was for the mutation in the respective pathway to be a consequence of elevated mutational burden. For that, we compared the odds ratios between mutation rates within the respective pathway to the ploidy-adjusted global mutation rates in the non-mutated and mutated groups. It allowed for discriminating the cases such as damage sensing pathway mutations in melanomas (SKCM), which is according to this test very likely to be a consequence of elevated mutation burden, whereas MMR deficient uterine cancers (UCEC) have an odds ratio of having mutations in the pathway which is much higher than that for MMR proficient samples.

### 3.4.2 Mutational profiles

In order to identify a change in the mutation spectrum between DNA repair proficient and deficient samples, we regressed out several endogenous processes (signatures SBS1 - deamination of methylated cytosines at CpG sites, SBS2 and SBS13 - APOBEC mutations, SBS5 and SBS30 - flat clock-like signatures associated with patient age) using the NMF procedure minimizing KL divergence with the signature matrix being fixed at values from [24]. We then selected only the samples where at least 100 mutations were left in order to get a reliable profile of mutations. Then we calculated cosine distances between the groups of non-mutated samples within certain cancer type, samples with monoallelic defects and samples with biallelic defects in a given pathway (**Supplementary Figure 1d**).

# 4. dNdS analysis across DNA repair genes in TCGA

## 4.1. Sample selection

dN/dS analysis of the ratios of numbers of expected vs observed non-synonymous and synonymous variants were performed using the trinucleotide model according to [5]. Background mutation rates were estimated using all the genes except for consistently undercovered genes [23]. The dN/dS ratios of interest were compared to 1 (the dN/dS ratio under the neutral evolution model), and the significance of selection was assessed by comparing the relevant $\chi 2$ statistic of the dN/dS ratio to $\chi^2$ distribution with *df* = 1.

## 4.2. Pathway-level analysis

For pathway-level analyses, we ran dNdSCV analysis separately within each cancer type using the samples with less than 1000 coding mutations. For each combination of pathway and cancer type, we estimated the global dNdS ratio over the list of core genes within that pathway, and compared them to 1 using $\chi^2$-statistic of the squared z-score of $\log(dN/dS_{pathway/cancer})$, with FDR control performed using Benjamini-Hochberg procedure. The resulting values are depicted in **Supplementary Figure 3h** and provided in **Supplementary Data 5**.

## 4.3. Gene-level analysis

For gene-level analyses, we estimated the global and per-gene dNdS values across all cancer types using all samples with less than 1000 coding mutations. dNdSCV analysis was performed for the extended set of 248 DNA repair associated genes (**Supplementary Data 3**) across all cancer types. For each gene, we estimated the dNdS ratio for missense and nonsense mutations, and compared them to 1 using $\chi^2$-statistic of the squared z-score of $\log(dN/dS_{gene})$, with FDR control performed using Benjamini-Hochberg procedure. The resulting values are depicted in **Supplementary Figure 3h** and provided in **Supplementary Data 5**.

# 5. Interaction analysis in human data

## 5.1 Damage-repair interactions in cancer: motivation

Following up on previous work on uterine cancers that revealed distinct mutational signatures associated with MMR or POLE exonuclease activity deficiency, and the combination of both [25], we developed a model to quantify non-additive signature changes between different groups of samples assuming that it is the spectrum associated with POLE proofreading deficiency that is altered in MMR+POLE deficient samples (**Methods**). Using this approach, we confirmed the increased C>A mutagenesis in a NpCpT context in samples with concurrent POLE mutations and MMR defects (**Supplementary Figure 3a,b**).

## 5.2 Model specification

The sampling model for human data was set up as described in Methods using MCMC sampling via R package "greta", v. 0.2.3 (http://CRAN.R-project.org/package=greta). All models were run in 4 chains up to 1000 or 2000 warm-up and 1000 post warm-up samples to ensure convergence. The codes for all the analyses are available on GitHub: http://github.com/gerstung-lab/signature-interactions .

We ran 4 chains of sampling, and claimed an effect being real if it was consistently assigned to the same signature. The final number of signatures was selected based on the convergence, similarity between signatures and feasibility of effect assignment, as the model tends to fluctuate or duplicate most variable signals when the dimensions chosen is too high.

## 5.3. POLE proofreading domain mutations and MMR deficiency

The effects of interaction between POLE and MMR defects were investigated using the uterine cancer cohort from TCGA (UCEC TCGA project) as indicated in [25]. Microsatellite instability (MSI) is used as a read-out for MMR deficiency: samples classified as MSI-H by Bethesda protocol (available in TCGA Clinical Explorer [26]) were considered to be MMR deficient, samples classified as MSI-L (low) or MSS (microsatellite stable) were considered to be MMR proficient. Samples with missense mutations in POLE proofreading domain (amino acids 267-472) were considered to have compromised proofreading activity. Overall, we analyzed 546 samples, 167 of which were labeled as MMR deficient, 55 - as having $POLE^{exo}$ mutations, and 15 - as having both deficiencies. We identified 5 signatures, with a signature similar to COSMIC SBS10 signature (the one associated with POLE proofreading domain defects) being subject to interactions with different $POLE^{exo}$ mutations and $POLE^{exo}$+MSI factor. In line with associations suggested previously, the transformation of POLE signature upon MMR deficiency closely resembled COSMIC signature SBS14, and signatures C1-2 from [25] (**Supplementary Figure 3a**).

In line with the initial study, we further looked into differences in $POLE^{exo}$ signature across samples with different mutations in POLE proofreading domain, and found the two hotspot mutations P286R and V411L producing different profiles compared to each other and the rest of $POLE^{exo}$ mutations. With our approach, we were able to better refine the modulation related to each mutation (**Supplementary Figure 3b**). Our findings confirm the concept of different mutations across POLE proofreading domain introducing different changes to the activity of the polymerase through variability in structural changes of the protein.

## 5.4. Tissue-specific effects in MMR deficiency

To test the effect of tissue type on the appearance of MMR deficiency signature, we screened all TCGA samples for the defects in MMR genes MLH1, PMS2, MSH2, MSH3 and MSH6 (we excluded MLH3 for the lack of samples with just the MLH3 mutation and visible MMR deficiency phenotype). Samples were labeled as having MMR defects if they had an impactful somatic mutation in MMR pathway (**Supplementary Data 3**) or were clinically determined to have MSI-H status as per TCGA Clinical Explorer [26].

The samples were further filtered to exclude ones with less than 100 mutations or more than 20000 mutations, and the ones whose mutational profile was dominated by a strong mutagenic process: APOBEC, POLE, UV or tobacco (i.e. we excluded those with cosine similarity higher than 0.7 to the respective signatures: SBS2, SBS13, SBS10a+SBS10b, SBS7 combined, SBS4). From the remaining samples, we selected data from 8 cancer types: breast (BRCA), cervical (CESC), head and neck (HNSC), stomach (STAD), colorectal (COAD and READ), liver (LIHC), lung (LUAD and LUSC), prostate (PRAD), and uterine cancers (UCEC). In total, we analysed a dataset of 782 samples. For testing the effects of each of the MMR genes being mutated, we used 6 covariates, one for each gene. To test tissue effects, we used colorectal as the default tissue, and fitted 7 interaction effects for other tissues - breast, cervix, head and neck, lung, stomach, uterus, liver.

## 5.5. Temozolomide and O-6-methylguanine-DNA methyltransferase

O-6-methylguanine-DNA methyltransferase (MGMT) methylation data as well as the somatic mutations from exomes of glioblastoma multiformae (GBM) samples treated with temozolomide (TMZ) were taken from previous studies [7]. The effect estimation model was run on 96 substitution types considering 17 samples treated with TMZ: 11 with wild-type MGMT and 6 with MGMT hypermethylation. The model showed best results for 2 signatures, one similar to COSMIC signature 1 and another one flat (**Figure 3d**), and suggested that the estimated change of the second signature is higher than 100-fold.

## 5.6. APOBEC mutagenesis and REV1/UNG defects

In order to estimate the modulation of APOBEC related signature in response to defects in REV1/UNG, we analyzed 842 TCGA tumour samples with significant APOBEC contribution (with cosine similarity of at least 0.8 to a combination of COSMIC signatures SBS2 and SBS13) which had between 50 and 15000 mutations per exome. We further stratified these samples into REV1/UNG wild-type (794 samples) and mutated (48 samples with at least monoallelic defects in *REV1* or *UNG*). Analysis of APOBEC-specific T[C>A/C/T]N mutations showed that defects in REV1 and/or UNG lead to an 8% decrease in the ratio between C>G and C>T mutations. Signature extraction with one signature showed a slight relative increase of C>T as part of the signature (**Figure 4c**).

## 5.7. UV exposure and xeroderma pigmentosum (XP) patients

The UV exposure signature was assessed in NER-proficient and deficient skin tumours using whole genome data from cutaneous squamous cells carcinomas of 5 XP (all XPC-deficient) patients between 2 and 10 years of age, and 8 patients without XP between 61 and 87 years [27] (**Figure 5d**). Variant calls were obtained from [28]. Signature extraction with one signature demonstrated a shift in the signature and a more than 100-fold change in the age-adjusted mutation rate (**Figure 5d**).

## 5.8. UV exposure and somatic defects NER

NER effects in UV-associated melanomas were estimated using samples from TCGA SKCM project where the similarity to a combination of COSMIC signatures SBS7a and SBS7b (previously associated with UV) was higher than 0.8. 397 patients were tested for having either expression of any of the NER genes in the tumour sample below 20% of the median level in the dataset, or a somatic biallelic deactivation of a core NER gene in the tumour

sample as described above in section 4.2.1. As the numbers were low, we also included samples with a high impact germline variant (homozygous or heterozygous) as predicted using Ensembl VEP [29].
The final set contained 9 samples labeled as NER defective.
We estimated the effects of having defects in the NER pathway using a single signature. NER did not show a dramatic change in the profile of UV signature, only produced a 5% increase in C>T mutations, but led to an overall 2-fold increase in the mutation burden per year (**Supplementary Figure 3e**).

In order to check if this is an effect of NER defects, or simply a consequence of increased mutational burden, we simulated UV-induced mutations in NER genes according to each sample's total burden of synonymous mutations, and tested if the observed number of mutations in these genes across the dataset is in line with expected. The observed number of nonsynonymous mutations across NER genes followed the distribution we generated via simulations, which indicated that most of the signal was coming from the difference in mutational burden (**Supplementary Figure 3f**). Together with the high variability of this fold change effect, it led to the conclusion that there is no detectable effect of mutations in NER genes on the appearance of UV exposure signature.

We have also tested 59 samples with defects in TLS machinery but did not see any effect on either exposure coefficient or appearance of UV signature.

## 5.9. Smoking and NER defects

Effects of DNA repair machinery defects on the signature of tobacco smoke were estimated using samples from LUAD and LUSC projects [10,29]. Out of 905 samples across the two datasets, we selected 219 samples with high presence of COSMIC signature SBS4 (cosine similarity over 0.8), associated with tobacco smoking. Of these, 82 samples were labeled as being NER deficient, and 92 were labeled as having TLS defects based on somatic and germline damaging mutations, both homozygous and heterozygous. Having extracted two signatures most similar to smoking and APOBEC associated signatures, we did not find a significant effect of either NER or TLS on the exposure coefficient or mutational distribution of the signature associated with smoking (**Supplementary Figure 3g**).

# Supplementary References


1. Kucab, J. E. *et al.* A Compendium of Mutational Signatures of Environmental Agents. *Cell* (2019) doi:10.1016/j.cell.2019.03.001.
2. Alexandrov, L. B. *et al.* The repertoire of mutational signatures in human cancer. *Nature* **578**, 94–101 (2020).
3. Behjati, S. *et al.* Mutational signatures of ionizing radiation in second malignancies. *Nat. Commun.* **7**, 12605 (2016).
4. Knijnenburg, T. A. *et al.* Genomic and Molecular Landscape of DNA Damage Repair Deficiency across The Cancer Genome Atlas. *Cell Rep.* **23**, 239–254.e6 (2018).
5. Martincorena, I. *et al.* Universal Patterns of Selection in Cancer and Somatic Tissues. *Cell* **171**, 1029–1041.e21 (2017).
6. Meier, B. *et al.* Mutational signatures of DNA mismatch repair deficiency in C. elegans and human cancers. *Genome Res.* **28**, 666–675 (2018).
7. Kim, H. *et al.* Whole-genome and multisector exome sequencing of primary and post-treatment glioblastoma reveals patterns of tumor evolution. *Genome Res.* **25**, 316–327 (2015).
8. Cancer Genome Atlas Network. Genomic Classification of Cutaneous Melanoma. *Cell* **161**, 1681–1696 (2015).
9. Cancer Genome Atlas Research Network. Comprehensive genomic characterization of squamous cell lung cancers. *Nature* **489**, 519–525 (2012).
10. Cancer Genome Atlas Research Network. Comprehensive molecular profiling of lung adenocarcinoma. *Nature* **511**, 543–550 (2014).
11. Cancer Genome Atlas Research Network *et al.* Integrated genomic characterization of endometrial carcinoma. *Nature* **497**, 67–73 (2013).
12. Cancer Genome Atlas Research Network. Comprehensive molecular characterization of urothelial bladder carcinoma. *Nature* **507**, 315–322 (2014).

13. Kucab, J. E. *et al.* A Compendium of Mutational Signatures of Environmental Agents. *Cell* (2019) doi:10.1016/j.cell.2019.03.001.
14. Alexandrov, L. *et al.* The Repertoire of Mutational Signatures in Human Cancer. *bioRxiv* (2018) doi:10.1101/322859.
15. Matsumura, S., Fujita, Y., Yamane, M., Morita, O. & Honda, H. A genome-wide mutation analysis method enabling high-throughput identification of chemical mutagen signatures. *Scientific Reports* vol. 8 (2018).
16. Flibotte, S. *et al.* Whole-genome profiling of mutagenesis in Caenorhabditis elegans. *Genetics* **185**, 431–441 (2010).
17. Boot, A. *et al.* In-depth characterization of the cisplatin mutational signature in human cell lines and in esophageal and liver tumors. *Genome Res.* **28**, 654–665 (2018).
18. Szikriszt, B. *et al.* A comprehensive survey of the mutagenic impact of common cancer cytotoxics. *Genome Biol.* **17**, 99 (2016).
19. Kucab, J. E. *et al.* A Compendium of Mutational Signatures of Environmental Agents. *Cell* (2019) doi:10.1016/j.cell.2019.03.001.
20. Roth, D. B., Porter, T. N. & Wilson, J. H. Mechanisms of nonhomologous recombination in mammalian cells. *Mol. Cell. Biol.* **5**, 2599–2607 (1985).
21. Pearl, L. H., Schierz, A. C., Ward, S. E., Al-Lazikani, B. & Pearl, F. M. G. Therapeutic opportunities within the DNA damage response. *Nature Reviews Cancer* vol. 15 166–180 (2015).
22. Van Loo, P. *et al.* Allele-specific copy number analysis of tumors. *Proc. Natl. Acad. Sci. U. S. A.* **107**, 16910–16915 (2010).
23. Wang, V. G., Kim, H. & Chuang, J. H. Whole-exome sequencing capture kit biases yield false negative mutation calls in TCGA cohorts. *PLoS One* **13**, e0204912 (2018).
24. Alexandrov, L. *et al.* The Repertoire of Mutational Signatures in Human Cancer. *bioRxiv* 322859 (2018) doi:10.1101/322859.
25. Haradhvala, N. J. *et al.* Distinct mutational signatures characterize concurrent loss of

polymerase proofreading and mismatch repair. *Nat. Commun.* **9**, 1746 (2018).

26. Lee, H., Palm, J., Grimes, S. M. & Ji, H. P. The Cancer Genome Atlas Clinical Explorer: a web and mobile interface for identifying clinical--genomic driver associations. *Genome Med.* **7**, 112 (2015).

27. Bradford, P. T. *et al.* Cancer and neurologic degeneration in xeroderma pigmentosum: long term follow-up characterises the role of DNA repair. *J. Med. Genet.* **48**, 168–176 (2011).

28. Sabarinathan, R., Mularoni, L., Deu-Pons, J., Gonzalez-Perez, A. & López-Bigas, N. Nucleotide excision repair is impaired by binding of transcription factors to DNA. *Nature* **532**, 264–267 (2016).

29. McLaren, W. *et al.* The Ensembl Variant Effect Predictor. *Genome Biol.* **17**, 122 (2016).
