# Supplementary material for: Mutational signatures are jointly shaped by DNA damage and repair
Source: Nat Commun. 2020 May 1;11:2169. doi: 10.1038/s41467-020-15912-7 (PMC7195458; doi:10.1038/s41467-020-15912-7)
Supplement: Supplementary file 3 — Description of Additional Supplementary Files [file 41467_2020_15912_MOESM3_ESM.pdf]

Description of Additional Supplementary Files

File Name: Supplementary Data 1

Description: Tables with annotations for each sample (genotype, exposure, dose) and mutation counts for all *C. elegans* samples.

File Name: Supplementary Data 2

Description: Experimental mutational signatures for genetic (including wild-type and DNA repair deficiencies) and mutagenic (12 genotoxins used in the study) factors in *C. elegans* with their 95% credible intervals.

File Name: Supplementary Data 3

Description: Map of DNA repair genes with their corresponding pathways, TCGA cancer project abbreviations, and TCGA samples with mutations in DNA pathways. Total deactivation stands for biallelic loss or epigenetic silencing as described in Supplementary Methods.

File Name: Supplementary Data 4

Description: Genotoxin-repair interaction effects in *C. elegans* mutagen exposure experiments: log fold-changes of mutagen signature per mutation class, and dose-dependent genotype amplification factors along with their 95% credible intervals.

File Name: Supplementary Data 5

Description: Maximum likelihood estimates of dN/dS values (column “mis_mle” for missense mutations, “non_mle” for nonsense mutations), their 95% confidence intervals, p-values and q-values for DNA repair associated genes across all cancer types and for DNA repair pathways in different cancer types.

File Name: Supplementary Data 6

Description: VCF files with filtered variants (base substitutions, indels and structural variants) for each sample.
