# Supplementary material for: Mutational signatures are jointly shaped by DNA damage and repair
Source: Nat Commun. 2020 May 1;11:2169. doi: 10.1038/s41467-020-15912-7 (PMC7195458; doi:10.1038/s41467-020-15912-7)
Supplement: Supplementary file 10 — Reporting Summary [file 41467_2020_15912_MOESM10_ESM.pdf]

nature research

Corresponding author(s): Gerstung

Last updated by author(s): Dec 20, 2019

Reporting Summary

Nature Research wishes to improve the reproducibility of the work that we publish. This form provides structure for consistency and transparency in reporting. For further information on Nature Research policies, see Authors & Referees and the Editorial Policy Checklist .

Statistics

For all statistical analyses, confirm that the following items are present in the figure legend, table legend, main text, or Methods section.

n/a	Confirmed	
☐	☒	The exact sample size (*n*) for each experimental group/condition, given as a discrete number and unit of measurement
☐	☒	A statement on whether measurements were taken from distinct samples or whether the same sample was measured repeatedly
☐	☒	The statistical test(s) used AND whether they are one- or two-sided<br>*Only common tests should be described solely by name; describe more complex techniques in the Methods section.*
☐	☒	A description of all covariates tested
☐	☒	A description of any assumptions or corrections, such as tests of normality and adjustment for multiple comparisons
☐	☒	A full description of the statistical parameters including central tendency (e.g. means) or other basic estimates (e.g. regression coefficient) AND variation (e.g. standard deviation) or associated estimates of uncertainty (e.g. confidence intervals)
☐	☒	For null hypothesis testing, the test statistic (e.g. *F*, *t*, *r*) with confidence intervals, effect sizes, degrees of freedom and *P* value noted<br>*Give P values as exact values whenever suitable.*
☐	☒	For Bayesian analysis, information on the choice of priors and Markov chain Monte Carlo settings
☒	☐	For hierarchical and complex designs, identification of the appropriate level for tests and full reporting of outcomes
☒	☐	Estimates of effect sizes (e.g. Cohen's *d*, Pearson's *r*), indicating how they were calculated

Our web collection on statistics for biologists contains articles on many of the points above.

Software and code

Policy information about availability of computer code

Data collection	Raw sequencing reads from C. elegans were aligned using bwa 0.5.9. Variant calling was performed using Caveman 1.11.2, Pindel 2.2.5, DELLY 0.7.8.
Data analysis	Data analysis was performed using R 3.5.1 and Bioconductor 3.8, including VariantAnnotation 1.28.10 and greta 0.2.3. Codes and full package versions available at github.com/gerstung-lab/signature-interactions.

For manuscripts utilizing custom algorithms or software that are central to the research but not yet described in published literature, software must be made available to editors/reviewers. We strongly encourage code deposition in a community repository (e.g. GitHub). See the Nature Research guidelines for submitting code & software for further information.

Data

Policy information about availability of data

All manuscripts must include a data availability statement. This statement should provide the following information, where applicable:

- Accession codes, unique identifiers, or web links for publicly available datasets
- A list of figures that have associated raw data
- A description of any restrictions on data availability

C. elegans sequencing data are available under ENA Study Accession Numbers ERP000975 and ERP004086. TCGA mutation, gene expression and methylation data are available at GDC and ICGC websites, http://gdc.cancer.gov/ and http://icgc.org .

Field-specific reporting

Please select the one below that is the best fit for your research. If you are not sure, read the appropriate sections before making your selection.

Life sciences study design

All studies must disclose on these points even when the disclosure is negative.

Sample size	No sample size calculations were performed for this exploratory screen. Each experiment was run in triplicates in order to estimate the experimental variance within each set of replicates. Number of lines for mutation accumulation experiments was defined based on strain availability and experimental handling capacity with inclusion of wild-type controls in each experiment. Number of genotoxin exposure experiments as well as the set of genotoxin doses tested were defined by handling capabilities, research question asked, and progeny viability observed following genotoxin dose exposure (no more than 50% reduction in the viability of embryos).
Data exclusions	Samples with insufficient DNA quality were excluded (16 samples removed because of failed sequencing, 31 samples failed the quality control step, and 71 were removed because of mislabeling). Variants were filtered as outlined in Supplementary Methods, Section 2
Replication	Biological experiments were run in triplicates until reaching F20 or F40 for mutation accumulation experiments, and for each dose of genotoxin in mutagen exposure experiments in order to estimate the variance within each experiment. For 7/54 mutation accumulation experiments and 15/196 damage-repair interaction experiments, DNA extraction or sequencing failed in at least one set of replicates. For 2/54 MA experiments and 14/196 interaction experiments, two our of three samples failed at DNA extraction or sequencing step at least one set of replicates (Supplementary Data 1).
Randomization	No randomisation was applied as the C. elegans test population was homogeneous in each of the experiments
Blinding	No blinding was applied as the experimental design and quantitative analysis did not allow for any subject- or observer-based biases

Reporting for specific materials, systems and methods

We require information from authors about some types of materials, experimental systems and methods used in many studies. Here, indicate whether each material, system or method listed is relevant to your study. If you are not sure if a list item applies to your research, read the appropriate section before selecting a response.

Materials & experimental systems

n/a	Involved in the study
☒	☐ Antibodies
☒	☐ Eukaryotic cell lines
☒	☐ Palaeontology
☐	☒ Animals and other organisms
☒	☐ Human research participants
☒	☐ Clinical data

Methods

n/a	Involved in the study
☒	☐ ChIP-seq
☒	☐ Flow cytometry
☒	☐ MRI-based neuroimaging

Animals and other organisms

Policy information about studies involving animals; ARRIVE guidelines recommended for reporting animal research

Laboratory animals	C. elegans N2 Bristol reference strain TG1813 hermaphrodites; all mutant lines were back crossed 6 times to TG1813. Genotoxin treatment was applied at late L4 and early adult stages, and their F1 progeny were picked at L4 stage and clonally expanded for sequencing.
Wild animals	No wild animals were involved
Field-collected samples	No field-collected samples were used
Ethics oversight	No ethical approval was required for work involving C. elegans.

Note that full information on the approval of the study protocol must also be provided in the manuscript.
